# Designing sensitive viral diagnostics with machine learning

**DOI:** 10.1038/s41587-022-01213-5

**Published:** 2022-03-03

**Authors:** Hayden C. Metsky, Nicole L. Welch, Priya P. Pillai, Nicholas J. Haradhvala, Laurie Rumker, Sreekar Mantena, Yibin B. Zhang, David K. Yang, Cheri M. Ackerman, Juliane Weller, Paul C. Blainey, Cameron Myhrvold, Michael Mitzenmacher, Pardis C. Sabeti

**Affiliations:** 1 Broad Institute of MIT and Harvard, Cambridge, MA USA; 2 Department of Electrical Engineering and Computer Science, MIT, Cambridge, MA USA; 3 Virology Program, Division of Medical Sciences, Harvard Medical School, Boston, MA USA; 4 Biophysics Program, Harvard Medical School, Boston, MA USA; 5 Bioinformatics and Integrative Genomics Program, Department of Biomedical Informatics, Harvard Medical School, Boston, MA USA; 6 Department of Organismic and Evolutionary Biology, Harvard University, Cambridge, MA USA; 7 Department of Biological Engineering, MIT, Cambridge, MA USA; 8 Koch Institute for Integrative Cancer Research at MIT, Cambridge, MA USA; 9 Department of Molecular Biology, Princeton University, Princeton, NJ USA; 10 School of Engineering and Applied Sciences, Harvard University, Cambridge, MA USA; 11 Department of Immunology and Infectious Diseases, Harvard T.H. Chan School of Public Health, Harvard University, Boston, MA USA; 12 Howard Hughes Medical Institute, Chevy Chase, MD USA

**Keywords:** Computational biology and bioinformatics, Infectious-disease diagnostics, Viral evolution

## Abstract

Design of nucleic acid-based viral diagnostics typically follows heuristic rules and, to contend with viral variation, focuses on a genome’s conserved regions. A design process could, instead, directly optimize diagnostic effectiveness using a learned model of sensitivity for targets and their variants. Toward that goal, we screen 19,209 diagnostic–target pairs, concentrated on CRISPR-based diagnostics, and train a deep neural network to accurately predict diagnostic readout. We join this model with combinatorial optimization to maximize sensitivity over the full spectrum of a virus’s genomic variation. We introduce Activity-informed Design with All-inclusive Patrolling of Targets (ADAPT), a system for automated design, and use it to design diagnostics for 1,933 vertebrate-infecting viral species within 2 hours for most species and within 24 hours for all but three. We experimentally show that ADAPT’s designs are sensitive and specific to the lineage level and permit lower limits of detection, across a virus’s variation, than the outputs of standard design techniques. Our strategy could facilitate a proactive resource of assays for detecting pathogens.

## Abstract (web summary)

Viral diagnostics with maximum sensitivity are designed using machine learning and combinatorial optimization.

## Main

Recent advances in nucleic acid detection have enriched infectious disease diagnostics and surveillance^1–8^. Yet there has been limited progress in enriching diagnostics and surveillance through computational design. That is surprising in light of machine learning and optimization capabilities, and the explosion of viral genomic data. Designing viral assays from genomic data is done largely by hand, without well-defined objectives.

Machine learning and optimization methods would benefit viral detection by designing assays that are more sensitive than existing ones. These methods could also enable a proactive resource of assays that are broadly effective across viral variation and provide rapid design of new assays. Here, we demonstrate these capabilities by developing and experimentally validating an approach that combines a deep learning model with combinatorial optimization.

We provide advances in three areas: (1) predicting the enzymatic activity of a diagnostic; (2) integrating a virus’s variation optimally into the design of a diagnostic; and (3) designing diagnostics rapidly at scale.

The first challenge we address is predicting a diagnostic’s activity when detecting a nucleic acid target. The most advanced methods, which focus on quantitative PCR (qPCR)^9–15^, usually make binary predictions—an assay will detect a viral target or will not—according to thermodynamic criteria and heuristics. Heuristics include constraining the number and positions of assay–target mismatches. Yet binary predictions are rudimentary because they still call for experimental assay comparisons and probably miss the optimum. Quantitative predictions of an enzyme’s activity when detecting a target could enhance sensitivity. In contrast to current paradigms, our approach uses experimental data and machine learning to predict enzymatic activity from nucleotide sequences. We form the largest dataset on diagnostic performance to our knowledge, concentrated on CRISPR-based diagnostics. We train a neural network to predict a CRISPR enzyme’s activity during detection, corresponding to diagnostic sensitivity.

Machine learning models have been built for CRISPR systems^16–18^ to predict a guide’s *cis* cleavage activity (for example, knockdown efficacy). Several^18,19^ models focus on CRISPR–Cas13 using handcrafted features, including one model^18^ applied^20,21^ to Cas13d guides for antiviral RNA knockdown. Cas13a, by contrast, has diagnostic applications and is our focus in this paper. Its collateral (*trans*) cleavage activity, triggered by target recognition, enables diagnostics yet that activity is more challenging to screen in high-throughput than *cis* cleavage, which can be measured by sequencing. Also, to the best of our knowledge, no previous study has applied deep learning to predict Cas13 guide activity. While we concentrate on CRISPR-based viral diagnostics, our approach applies to other nucleic acid technologies and to non-viral targets.

The second challenge we confront is viral variation. Influenza A virus (FLUAV) quantitative PCR with reverse transcription (RT–qPCR) tests often have false-negative rates over 10% (nearly 100% on some strains) owing to variation^22–24^, and the issue besets other viruses^25–29^. Diagnostic design methods that account for variation generally follow one of two paradigms. One^10,12,13^ identifies conserved genomic regions and designs an assay targeting them, usually matching one reference sequence: this is inadequate because conserved regions are rarely free of variation, and targeting them may not provide optimal sensitivity and antagonizes specificity among viruses. The second paradigm^9,11,14,15^ minimizes an assay’s complexity, constrained to detecting a sufficient extent of variation: by handling variation through a constraint, it does not expressly optimize sensitivity. We integrate a virus’s variation into an objective function and, using our predictive model, maximize sensitivity across that variation.

**Extended Data Fig. 1:**
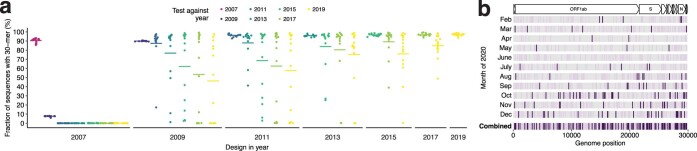
Emerging viral variation over time and the effect on diagnostic performance. **a**, Diagnostic performance for influenza A virus subtyping may degrade over time, even considering conserved sites. At each year, we select the 15 most conserved 30-mers from recent sequences for segment 6 (N) for all N1 subtypes; each point is a 30-mer. Plotted value is the fraction of sequences in subsequent years (colored) that contain the 30-mer; bars are the mean. To aid visualization, only odd years are shown. 2007 N1 30-mers are absent following 2007 owing to antigenic shift during the 2009 H1N1 pandemic. **b**, Variation along the SARS-CoV-2 genome emerging over time during 2020. Bottom row (‘Combined’) shows all 1,131 single nucleotide polymorphisms, among 361,460 genomes, that crossed 0.1% or 1% frequency between February 1, 2020 and the end of 2020—i.e., (i) at <0.1% frequency in genomes collected before February 1 and 0.1–1% frequency by December 31 (light purple); or (ii) at <1% frequency before February 1 and at ≥1% frequency by December 31 (dark). Other rows show the month in which each polymorphism crosses the frequency threshold.

The third challenge we tackle is scalability. The number of viral genome sequences is growing exponentially^30,31^, reflecting viral evolution and emergence (Supplementary Fig. 1). This growth impels periodic assay redesign. FLUAV subtyping assays lose sensitivity over time (Extended Data Fig. 1a and Supplementary Fig. 2). In the case of COVID-19, mutations accumulated on early genome sequences (Extended Data Fig. 1b) and some created failures in widely used diagnostic targets^32,33^. Yet current design paradigms, which require curating input data, are laborious. To overcome this obstacle, we introduce ADAPT (https://adapt.run), a system that implements our approach using the latest viral genomes from public databases. ADAPT is fully automated and operates at scale.

We applied ADAPT to design maximally sensitive, species-specific diagnostics for the 1,933 viral species known to infect vertebrates. We experimentally test ADAPT’s designs on several viruses using synthetic targets designed to encompass known variation. The results demonstrate that ADAPT provides designs with comprehensive detection and lineage-level specificity. ADAPT outperforms standard techniques that are based on conventional CRISPR diagnostic design heuristics and sequence conservation.

## Results

### Predicting activity of a CRISPR-based diagnostic

We first aim to predict a diagnostic’s enzymatic activity when detecting a viral target, which corresponds to its sensitivity, using a measurement-driven approach. We generated a dataset of fluorescence readout during detection reactions. From this dataset, we trained a machine learning model to predict enzymatic activity.

We focus on CRISPR–Cas13a^1,2^, in which Cas13a enzymes use guide RNAs to locate a target and subsequently exhibit collateral activity that cleaves fluorescent reporters, leading to a diagnostic readout. Earlier studies characterized reporter sequence requirements^2,34^ and established Cas13a guide design principles—such as the importance of the protospacer flanking site (PFS) and the mismatch-sensitive ‘seed’ region^35–37^—but have not measured collateral activity in high-throughput nor modeled it.

**Fig. 1:**
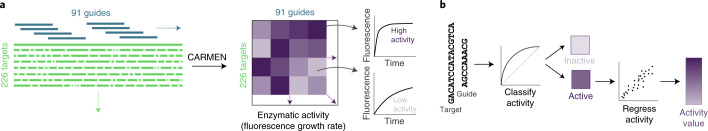
Measuring and modeling CRISPR–Cas13a detection activity. **a**, The library consists of an 865-nt-long wild-type target sequence and 91 guide RNAs complementary to it, along with 225 unique targets containing mismatches and varying PFS alleles relative to the wild type (19,209 unique guide–target pairs). We measure fluorescence every ~20 min for each pair and use the growth rate to quantify activity. **b**, We model activity for a guide–target pair in two parts: a classifier on all pairs and a regression model on the active pairs.

**Extended Data Fig. 2:**
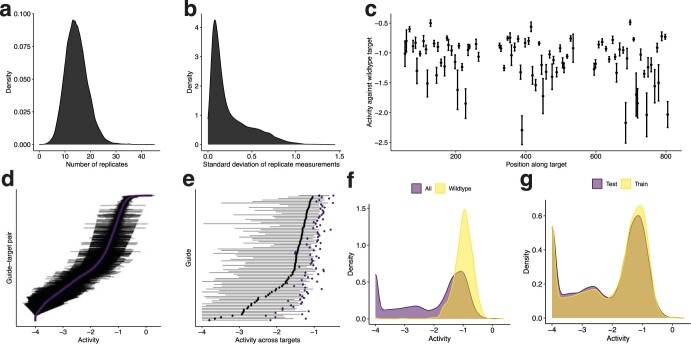
Dataset of CRISPR-Cas13a guide-target pairs. Measurements from the unique guide-target pairs in the dataset used for model training and testing. Activity is defined in Methods. **a**, Distribution of number of replicate activity measurements for each pair (including negative control pairs). **b**, Distribution of standard deviation across replicate activity measurements for each pair (including negative control pairs). **c**, Activity of each guide against the wild-type target (matching exactly), shown by their position along the target. Dot indicates the mean activity across n ≥ 40 wild-type target replicate measurements, shown with a 95% confidence interval. **d**, Variation in activity across guide-target pairs and among replicate measurements. Each row represents a guide-target pair. Purple dot indicates the mean activity across replicate measurements; pairs are sorted vertically by this value. Bars indicate the 95% confidence interval for the mean. **e**, Variation in activity between guides and across targets for each guide. Each row represents a guide. Black dot indicates the median activity across all targets and bars span the 20^th^ and 80^th^ percentiles of activity across all targets. Purple dot indicates the mean activity across the wild-type targets (matching the guide exactly). **f**, Distribution of activity across all guide-target pairs and only pairs with the wild-type target. **g**, Distribution of activity across all guide-target pairs in the training data and the pairs in the hold-out test data (the two sets do not overlap along the target or contain the same guides; Methods). In **d–g**, there are 10 resampled replicate activity measurements for each guide-target pair. We set a lower threshold of −4 on the activity owing to measurement limitations (see Supplementary Fig. 4b and Methods for details), so density shown at −4 includes guide-target pairs with true activity below this threshold. **a**, **b**, **f**, and **g** show kernel density estimates.

We designed a library of 19,209 unique LwaCas13a guide–target pairs (Fig. 1a and Supplementary Fig. 3a) to be tested using CARMEN^8^ (Combinatorial Arrayed Reactions for Multiplexed Evaluation of Nucleic acids), a droplet-based platform that performs parallel detection reactions. The library has a sequence composition representative of viral diversity, an average of 2.9 mismatches between each guide and target, and a variety of PFS alleles (Methods and Supplementary Fig. 3b,c). During each pair’s reaction, the intact reporter decays exponentially owing to Cas13a cleavage, and thus we use the negative of the decay to model fluorescence over time and determine its growth rate (Fig. 1a and Methods). The fluorescence growth rate is proportional to the enzymatic efficiency and concentration of a guide–target–Cas13a complex, so we evaluate the efficiency by holding the complex concentration constant (Supplementary Fig. 4). We define activity as the logarithm of the fluorescence growth rate. We measured the fluorescence arising from the library’s guide–target pairs every ~20 min and, from these measurements, calculated each pair’s activity (Extended Data Fig. 2).

**Fig. 2:**
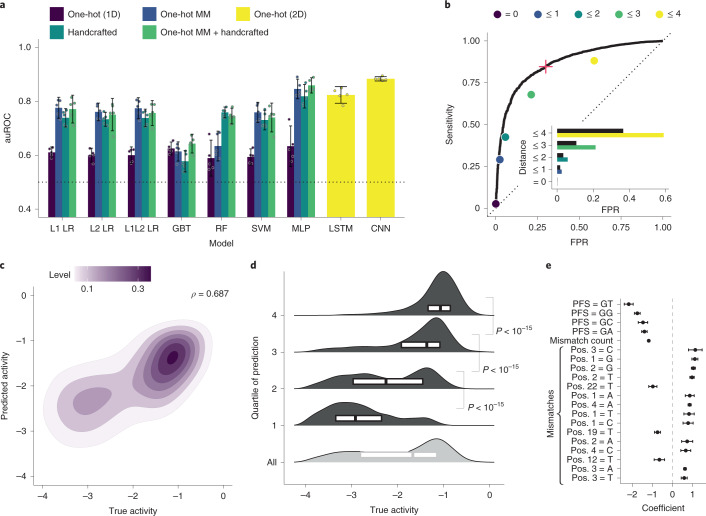
Predicting CRISPR–Cas13a detection activity. **a**, Model selection, for classification, with nested cross-validation. For each model and input type (color) on five outer folds, we performed a fivefold cross-validated hyperparameter search. The bar is the mean auROC over the *n* = 5 outer folds (each is a point) and the error bar is the 95% confidence interval. L1 LR and L2 LR, logistic regression; L1L2 LR, elastic net; GBT, gradient-boosted classification tree; RF, random forest; SVM, support vector machine; MLP, multilayer perceptron; LSTM, long short-term memory recurrent neural network; CNN, neural network with parallel convolutional filters and a locally connected layer. One-hot (1D), one-hot encoding of target and guide sequence independently, that is, without pairing of nucleotides; One-hot MM, one-hot encoding of target sequence nucleotides and mismatches in guide relative to the target; Handcrafted, curated features (Methods); One-hot (2D), one-hot encoding of target and guide sequence with encoded guide–target pairing. **b**, ROC curve, on a hold-out test set, of CNN classifying pairs as inactive or active. Points indicate sensitivity and false-positive rate (FPR) for baseline heuristic classifiers: a guide–target pair is active if it has a non-G PFS and the Hamming distance is within the specified threshold (color). Inset; comparison of the FPR between CNN (black) and baseline classifiers at equivalent sensitivity. The red plus indicates selected decision threshold. **c**, Results, on the hold-out set, of CNN predicting activities of active guide–target pairs. Contour color, point density. *ρ*, Spearman correlation. Extended Data Fig. 4 shows regression including inactive pairs. **d**, Same data as **c**. Each row contains one quartile of pairs divided by predicted activity (top row is predicted most active; light gray row combines all active pairs). Smoothed density estimates and interquartile ranges show the true activities. *P* values are from Mann–Whitney *U* tests (one-sided). **e**, Top 20 feature coefficients in L1 logistic regression model classifying activity with ‘One-hot MM + Handcrafted’ features. The dot is the mean over training on *n* = 5 splits and the error bar is the 95% confidence interval. Mismatch features indicate a mismatch with the indicated base being the complement of the spacer nucleotide; positions (Pos.) are relative to the target (28 is 5′ end of spacer).

Using our dataset, we developed a model to predict Cas13a activity from a guide–target pair. We use a two-step hurdle model: classifying a pair as inactive or active, and then regressing activity for active pairs (Fig. 1b and Supplementary Fig. 4b; 86.8% of the full dataset is labeled active). For classification, we performed nested cross-validation—fitting models multiple times on separate splits of the training data—to evaluate our fitting procedure and compare nine models using different inputs, including one-hot encodings (representing sequences with binary vectors) and handcrafted features. A deep convolutional neural network (CNN) classifier, using nucleotide sequences alone, outperforms the other models (Fig. 2a and Supplementary Fig. 5a). For regression, a CNN also outperforms other models, albeit with less improvement over simpler models (Supplementary Fig. 5b,c). The convolutional layers probably detect sequence motifs and mismatch patterns. In all model training, we accounted for measurement error (Methods). Our strategy for dividing data ensured that validation folds contained sets of cognate guide–target pairs, unrelated to data in the training folds, which mirrors real-world usage (Methods).

Our space of CNN models allows for both convolutional and locally connected filters of different widths (Supplementary Fig. 6). The latter learn distinct filters for different regions of the guide–target complex and our model search preferred to incorporate them together with convolutional filters (Supplementary Figs. 7 and 8). They may help the model uncover fixed spatial dependencies, such as mismatch-sensitive regions.

**Extended Data Fig. 3:**
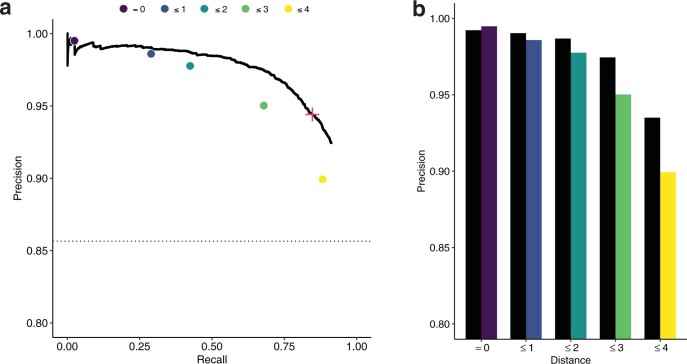
Precision-recall curve of classifier. **a**, Precision-recall (PR) curve of CNN model, which is used in ADAPT, classifying pairs as inactive or active on a hold-out test set. ROC curve is in Fig. 2b. Points indicate precision and recall for baseline heuristic classifiers, defined as choosing a guide-target pair to be active if and only if it has an active (non-G) PFS and the Hamming distance between the guide and target is within the specified threshold (color). Red ‘+’ indicates the decision threshold in ADAPT. Dashed line is precision of a random classifier (equivalently, the fraction of guide-target pairs that are active). **b**, Comparison of precision between CNN (black) and baseline classifiers (color as in **a**) at equivalent recall.

**Extended Data Fig. 4:**
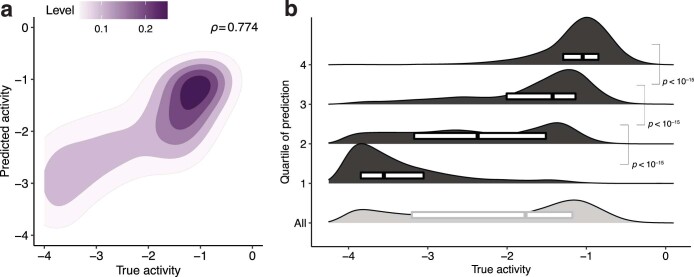
Regression results using all guide-target pairs. Results, on the hold-out test set, of a CNN trained to regress activity using all guide-target pairs (other regression data are trained and tested only on active pairs). We set a lower threshold of −4 on the activity owing to measurement limitations (see Supplementary Fig. 4b and Methods for details), so activities are bounded below at −4. **a**, Contour color, point density. *ρ*, Spearman correlation. **b**, Same data as **a**. Each row contains one quartile of pairs divided by predicted activity (top row is predicted most active), with the bottom row showing all guide-target pairs. Smoothed density estimates and interquartile ranges show the distribution of true activity for the pairs from each quartile. *P*-values are calculated from Mann-Whitney *U* tests (one-sided). The excess of inactive guide-target pairs in our data distorts the performance of this model and we do not use this model in ADAPT. We instead use the two-step hurdle model (Figs. 2c and 2d), as described in Methods, owing to the data’s distribution and the process we aim to model.

We evaluated our models’ performance on a hold-out test set of guide–target pairs (Methods) and against a standard Cas13a design heuristic. Our classifier performs well (area under the receiver operating characteristic curve (auROC) = 0.866; area under the precision–recall curve (auPR) = 0.972 with 85.6% of the test set being true positive; Fig. 2b and Extended Data Fig. 3a). When the guide and target are not identical, it yields a lower false-positive rate and higher precision than a heuristic classifying activity according to the PFS and guide–target divergence (Fig. 2b and Extended Data Fig. 3b). Our regression predicts the activity of active guide–target pairs (Spearman’s *ρ* = 0.687; Fig. 2c), accurately binning pairs into quartiles (Fig. 2d). Regression on both active and inactive pairs performs well (Spearman’s *ρ* = 0.774; Extended Data Fig. 4), but is less suited here than a hurdle model.

Further exploring our models, we considered that two features, the PFS and number of mismatches, could explain much of the performance. Yet the classification and regression CNNs retain accuracy when evaluated on individual PFS alleles and mismatch counts (Supplementary Figs. 9 and 10), albeit sometimes with lower performance than on the full dataset. Additional data similar to our current dataset would not be expected to improve performance (Supplementary Fig. 11).

**Extended Data Fig. 5:**
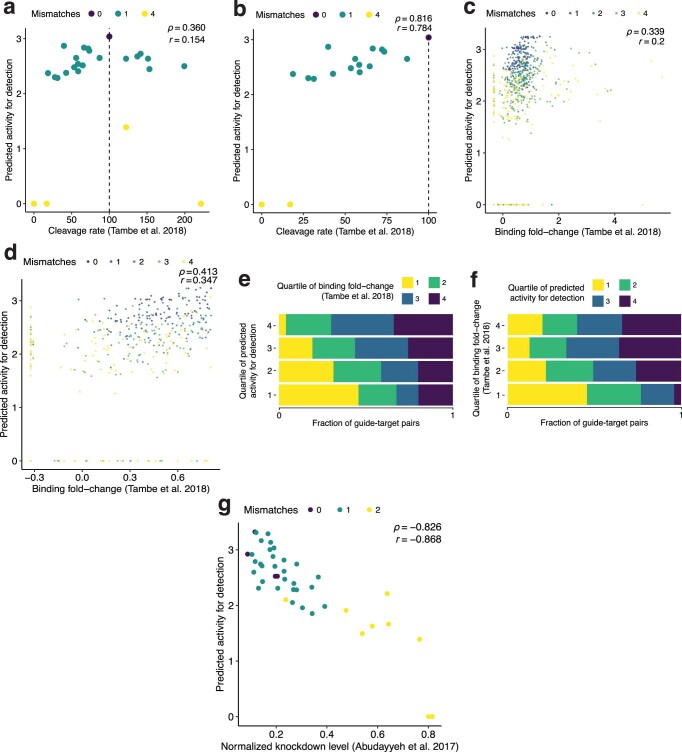
Comparisons with independent CRISPR-Cas13a datasets. **a**, Each point is a guide-target pair from experiments in ref. ^37^ measuring LbuCas13a nuclease activity. Horizontal axis is the measured, normalized percent cleavage rate relative to no mismatches and dashed line at 100 shows the value for no mismatches. Vertical axis is our model’s predicted LwaCas13a collateral cleavage activity. Colors indicate the number of mismatches in the guide-target pair. *ρ*, Spearman correlation; *r*, Pearson correlation coefficient. **b**, Same as **a**, but only for points where mismatches decrease the LbuCas13a cleavage rate—that is, points to the left of the dashed line. Considering this subset helps to mitigate differences arising from LbuCas13a’s higher overall collateral activity compared to LwaCas13a. **c**, Each point is a guide-target pair from experiments in ref. ^37^ measuring LbuCas13a–RNA binding affinity. Horizontal axis is the measured, regularized fold-change enrichment for binding to a target. Vertical axis is our model’s predicted LwaCas13a collateral cleavage activity. **d**, Same as **c**, but only for points where mismatches decrease the binding affinity compared to no mismatches. **e**, Same data as **c**. Each row contains one quartile of pairs divided by our model’s predicted activity (top row, 4, is predicted most active). Colors in each bar indicate the fraction of pairs belonging to each quartile of the binding affinity measurements (4 is highest binding). **f**, Same data as **c**. Each row contains one quartile of pairs divided by binding affinity measurements (top row, 4, is highest binding). Colors in each bar indicate the fraction of pairs belonging to each quartile of the predicted activities (4 is predicted most active). We do not expect a high correlation in **c–f** owing to differences between the variables being compared and between LbuCas13a and LwaCas13a; nevertheless, the relationship is consistent with binding being necessary, though not sufficient, to achieve collateral activity. **g**, Each point is a guide-target pair from experiments in ref. ^36^ measuring knockdown levels from LwaCas13a on-target *cis* cleavage. Horizontal axis is the measured knockdown level. In the normalized measurements, the non-targeting guide was set to a knockdown level of 1. Vertical axis is our model’s predicted LwaCas13a activity.

We tested our model on two independent datasets^36,37^. The comparisons provide independent validation of its accuracy (Spearman’s |*ρ*| = 0.816 and 0.826) and demonstrate its generalizability to other uses, such as predicting RNA knockdown (Supplementary Note 1 and Extended Data Fig. 5).

Precision matters greatly because we want confidence that designs predicted to be active are indeed active. In our design process, we set the classifier’s decision threshold to yield a precision of 0.975 (Methods, Figs. 1b and 2b, and Extended Data Fig. 3a).

**Extended Data Fig. 6:**
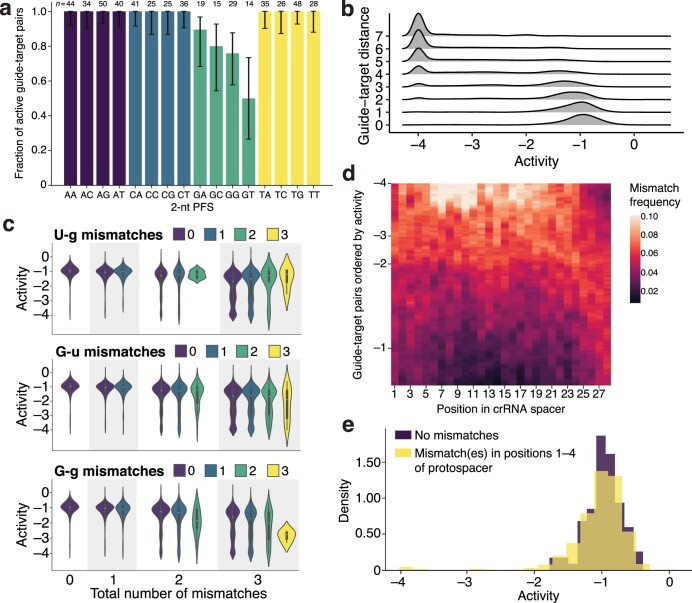
CRISPR-Cas13a guide-target activity. **a**, Fraction of guide-target pairs that are active for each 2-nt protospacer flanking site allele (PFS; i.e., the canonical Cas13a PFS together with the nucleotide adjacent on the 3′ side of the protospacer). For a pair to be active here, the median log(*k*) value across its replicates is > −2. Error bars represent 95% exact binomial confidence intervals. This analysis considers only matching guide-target pairs (i.e., 0 mismatches) and, when determining unique data points, includes 20-nt of flanking sequence context on each side of the protospacer; there are *n* = 509 such data points in total and value on top of each bar is the number (*n*) with each allele. **b**, Density of activity for different numbers of mismatches between guides and targets. Here, the number of mismatches is equivalent to Hamming distance. **c**, Effect of G-U pairing on activity. Top panel highlights U in the target and G in the crRNA spacer (U-g). Horizontal groupings (0, 1, 2, 3) are the total number of mismatches in guide-target pairs and the distributions in each grouping separate the pairs by the number of U-g mismatches, showing the density and interquartile ranges of activity; the yellow distribution shows pairs with 3 mismatches, all of which are U-g. Middle panel highlights G-u mismatches and bottom panel, as a benchmark, shows G-g. **d**, Profile of mismatches among guide-target pairs with similar activity. Each row in the heatmap represents a guide-target pair, ordered by activity, with those having the lowest activity on top; values on the left indicate activity. For each row *y*, we consider the 1,000 guide-target pairs with activity closest to the pair represented by *y*. Then, at each position *x* in the crRNA spacer sequence, we consider all mismatches at *x* across our dataset and calculate the fraction of them to which the 1,000 guide-target pairs, centered at *y*, contribute. We plot this fraction; higher values at a row indicate a preponderance of mismatches among the guide-target pairs with the activity represented by that row. **e**, Density of guide-target pairs that have no mismatches (purple) compared to those that have at least one mismatch in the first four positions of the protospacer and no mismatch elsewhere (yellow). As in **b**, here a G-U pair is counted as a mismatch. Positions in **d** are relative to the crRNA spacer sequence, while positions in **e** (and elsewhere) are relative to the target. In our analyses we set a lower threshold of −4 on the activity owing to measurement limitations (see Supplementary Fig. 4b and Methods for details), so in **b–e** guide-target pairs shown at −4 include pairs with true activity below this threshold; in **b** and **c**, densities drop slightly below −4 owing to smoothing.

Beyond modeling activity, we examined our dataset to understand LwaCas13a preferences. Previous studies identified weaker LwaCas13a activity when the PFS is G^1^ and characterized the preference in other orthologs^35–37^. In our data, G also reduces activity and, extending a position, GA, GC and GG provide higher activity than GT (Extended Data Fig. 6a), suggesting a more subtle PFS preference. Mechanistically, GT may reduce activity by preventing guide–target duplex separation^38^. Mismatches are another important consideration given viral variation, and increasing numbers generally reduce activity (Extended Data Fig. 6b). U-g mismatches (U in target, G in spacer) rescue activity in our data, though G-u mismatches do not (Extended Data Fig. 6c); while RNA binding might tolerate both wobble pairings, the asymmetry could stem from how they affect nuclease activation. Our dataset also clarifies, for LwaCas13a, the mismatch-sensitive region previously identified for LbuCas13a^37^ and LshCas13a^35^. Weak guide–target pairs are relatively likely to contain mismatches in positions 6–11 of the spacer, concordant with the known region, and there is high tolerance for mismatches on the 3′ end of the spacer (Extended Data Fig. 6d,e). Coefficients from linear models are consistent with these findings (Fig. 2e and Supplementary Fig. 12).

While our dataset and model focus on CRISPR–Cas13a, a similar measurement-driven approach could be applied to other viral nucleic acid detection technologies. The remainder of our work is model-agnostic.

### Designing maximally active assays across variation

Our model provides quantitative predictions that can be used within an optimization framework. We sought to design assays that are maximally active in detecting a virus’s variation. This formulation more explicitly optimizes sensitivity than design approaches^9–15^ that target conserved regions or handle sequence variation through a constraint.

**Fig. 3:**
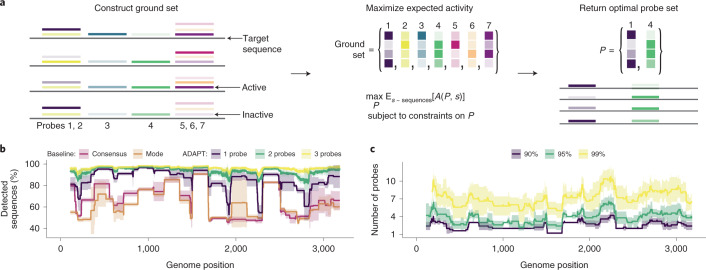
Maximizing sensitivity across genomic variation. **a**, Our approach for designing maximally active probe sets in a genomic region. We (1) determine a ground set of candidate probes (colored), which are representative subsequences; (2) compute an activity (shaded) between each probe and each target sequence *s*; (3) find a probe set *P*, a subset of the ground set, maximizing the expected activity *A*(*P*, *s*) between *P* and *s*, subject to soft and hard constraints on *P* (including on |*P*|, the number of probes). **b**, Fraction of Lassa virus (LASV; segment S) genomes detected, with different design strategies in a 200-nt sliding window, using a model in which 30-nt probes detect a target if they are within 1 mismatch, counting G–U pairs as matches. Consensus, probe-length consensus subsequence that detects the greatest number of genomes in the window; Mode, most abundant probe-length subsequence within the window. Our approach (ADAPT) uses hard constraints of 1–3 probes and maximizes activity. **c**, Number of probes when solving a different objective: minimizing the number of probes to detect *>*90%, *>*95% and *>*99% of LASV genomes using the model in **b**. In **b** and **c**, lines show the mean and shaded regions around them are 95% pointwise confidence bands, across sampled LASV genomes, calculated by bootstrapping, that is, randomly sampling genomes to be input to the design process.

We first formulate the problem of designing probe sequences across variation. We rely on all known sequences (*S*) within a genomic region (for example, amplicon) and our model that predicts activity between a probe and a targeted sequence. In the case of CRISPR-based diagnostics, probe sequences are guide RNAs; later, we address how to identify regions. We initially construct a ground set of possible probes, which are representative subsequences in *S*, using locality-sensitive hashing^39^. Our objective is to find the set *P* of probes, a subset of the ground set, that maximizes the expected activity when *P* detects *S* (Fig. 3a and Supplementary Note 2a). The expectation is over the sequences in *S*. Larger numbers of probes would require more detection reactions or, if they are multiplexed in one reaction, may interfere^40^, with the kinetic impact harming sensitivity^41^; thus, we impose a penalty and a hard constraint on the number of probes.

Having formulated an objective function, we developed an approach to maximize it using combinatorial optimization. We apply a fast randomized combinatorial algorithm^42^ for maximizing a non-negative and non-monotone submodular function under a hard constraint on the number of probes, which provides a probe set having an objective value near the optimal (Supplementary Note 2a). A more simple, canonical greedy algorithm^43^ for submodular maximization returns similar results in practice (Supplementary Fig. 13), though does not offer provable guarantees in our case.

We benchmarked our approach’s comprehensiveness across sequence variation. To make this benchmarking interpretable and independent from the predictive model, we chose an activity function that equals 1 if a probe is within 1 mismatch of a target (detected) and 0 otherwise (not detected); expected activity is equivalent to the fraction of genomes detected. Two simple but common strategies for constructing probes—using the consensus or most abundant sequence in a region—fail to capture diversity for Lassa virus and other diverse viruses (Fig. 3b and Supplementary Fig. 14a).

Our approach yields greater comprehensiveness than the simple strategies. Its designs detect more variation—even with one probe—across the genome, and the extent detected increases as we permit more probes (Fig. 3b and Supplementary Fig. 14a). If we compare against a combination of the most abundant subsequences—generalizing the baseline strategy that selects the single most abundant, to now use multiple probes—our approach still detects more variation (Supplementary Fig. 14b). That is expected because our approach explicitly maximizes detection over the sequences. A different objective function can minimize the number of probes subject to comprehensiveness constraints (Supplementary Note 2b, Fig. 3c and Supplementary Fig. 14c). On species with less diversity, simple strategies perform well (Supplementary Fig. 14a,b), suggesting that our approach is not always necessary. Nevertheless, options to target many regions of a genome facilitate genuine activity predictions and taxon-specificity, which constrain designs.

**Extended Data Fig. 7:**
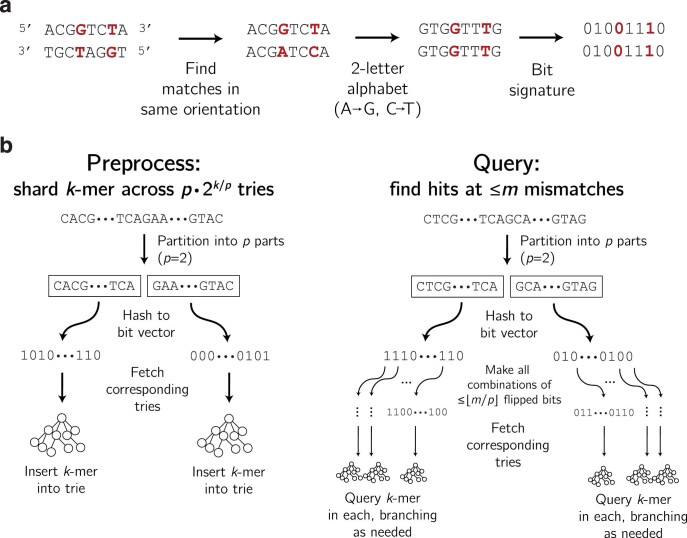
Sharding *k*-mers across tries for specificity queries. **a**, Constructing a bit signature after transforming a string to a two-letter alphabet, as described in Supplementary Note 3b. Two strings that match up to G-U base pairing (shown here as G-T) have the same bit signature. **b**, Left: Inserting a *k*-mer into the data structure of tries. Each *k*-mer is inserted into *p* tries, and there are *p* · 2^*k*/*p*^ tries in total. Right: Querying a *k*-mer for near neighbors (within *m* mismatches, sensitive to G-U base pairing as a match).

Viral detection assays must often distinguish between species or strains that are genetically similar. In patient diagnostics, related viruses can cause similar symptoms and a highly specific assay helps to identify the infection or rule out possibilities. Taxon-specificity is also essential to routine surveillance that tests for many viruses. We avoid cross-reactivity by constraining the ground set of probes to only ones deemed taxon-specific. Determining whether a probe is taxon-specific ought to tolerate multiple mismatches between probes and potential off-targets and, when the probes and targets are RNA (as with Cas13), G-U wobble base pairs (Supplementary Fig. 15). We developed a data structure and query algorithm that are fully tolerant of high divergence and G-U wobble base pairing (Extended Data Fig. 7). Evaluating a probe’s taxon-specificity is a computational bottleneck, and this method runs 10–100 times faster than a baseline simple data structure with the same capability (Supplementary Fig. 16). Supplementary Note 3 provides details.

### Designing comprehensive diagnostics at scale

**Fig. 4:**
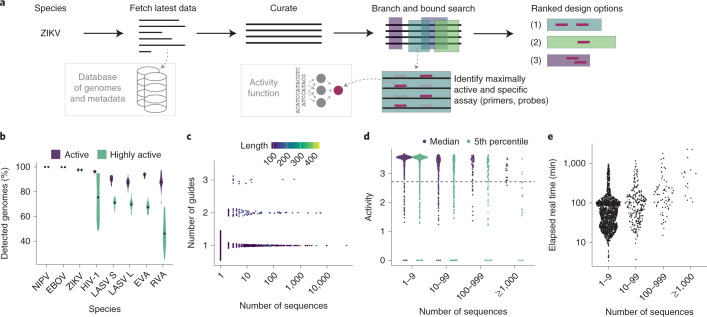
Large scale, end-to-end design with ADAPT. **a**, Overview of ADAPT’s steps. ADAPT accepts taxonomy identifiers, and fetches and curates their sequences from viral genome databases. It performs a branch and bound search to find genomic regions—each is an amplicon with primers—that contain maximally active and taxon-specific probe sets. ADAPT outputs the top design options, ranked by an objective function. **b**, Cross-validated evaluation of detection. For each species, we ran ADAPT on 80% of available genomes and evaluated performance, averaged over the top five design options, on the remaining 20%. Distributions are across 20 random splits of these genomes and dots indicate mean. Purple, fraction of genomes detected by primers and for which Cas13a guides are classified as active. Green, same except Cas13a guides also have an activity, predicted by the regression model, in the top 25% of our dataset (‘highly active’). NIPV, Nipah virus; EBOV, Zaire ebolavirus; ZIKV, Zika virus; LASV S/L, Lassa virus segment S/L; EVA, Enterovirus A; RVA, Rhinovirus A. **c**, Number of Cas13a guides in the top-ranked design option for each species in the vertebrate-associated virus designs. Color is the length of the targeted region (amplicon) in nt. **d**, Activity of each guide set with two summary statistics of its performance across known genome sequences: median and the 5th percentile taken across each species’ sequences. For the latter statistic, 95% of sequences are detected with predicted activity greater than or equal to the plotted value. The dashed line indicates the ‘high activity’ threshold from **b**. Sequences at 0 activity are predicted by the classifier to not be detected. Activities shown here are shifted up by 4 compared with the model output in Fig. 2. **e**, End-to-end elapsed real time running ADAPT. In **c**–**e**, each point is a vertebrate-associated viral species and the horizontal axis indicates its number of genome sequences.

To accommodate ever-growing viral genomic data, we built ADAPT. ADAPT designs assay options using our model-based optimization approach, while interfacing with viral genome databases to incorporate the latest available data (Fig. 4a).

ADAPT searches a viral genome to identify regions to target, scoring them according to their amplification potential and the activity of an optimal probe set. In the process, ADAPT designs amplification primers to achieve high coverage of sequence diversity. ADAPT’s genome-wide search follows the branch and bound paradigm and identifies the best *N* design options; users specify *N*, for which smaller values speed the search. Providing diverse design options allows for assays that target multiple distinct regions of a genome. ADAPT memoizes computations during its search, which decreases runtime by over 99% (Supplementary Fig. 17). Supplementary Note 4a,b details primer design and the search algorithm. ADAPT downloads and curates all near-complete or complete genomes from public databases^31^ for a specified virus taxonomy to use for design (Supplementary Note 4c). Fully automated assay design helps keep pace with viral evolution and emergence.

**Extended Data Fig. 8:**
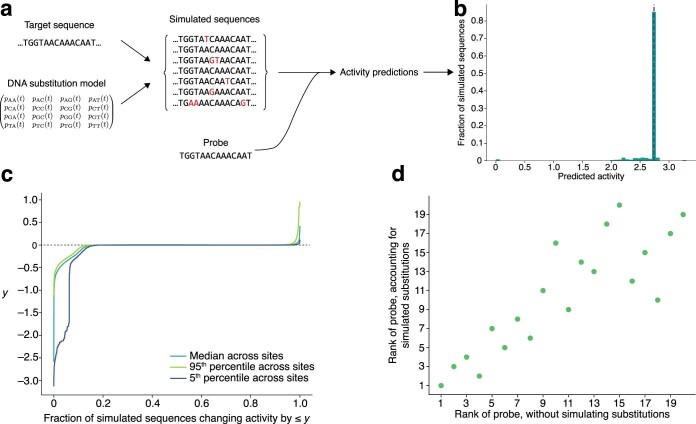
Estimating probe activity under forecasted substitutions. **a**, Sketch of proactive scheme to estimate probe performance, after a period of time, by forecasting relatively likely nucleotide substitutions. Starting with a target sequence and a GTR substitution model, we sample from a distribution of substitutions made to the original target sequence. In analyses that follow, we sample after 5 years. Against each simulated sequence, we predict the detection activity of a given probe using our Cas13a predictive model; across the simulated sequences, these predictions provide a distribution of activity under potential substitutions. **b**, Distribution of predicted detection activity across 10,000 simulated sequences that originate from a target sequence at one site in the SARS-CoV-2 genome. The probe is complementary to the original target sequence, except we randomly introduce a single mismatch. Most simulated sequences do not introduce any substitutions (i.e., they are identical to the original); the peak in the histogram (and vertical dashed line) represents these ones. Other than these simulated sequences, most simulated substitutions degrade the activity of the probe (left of the dashed line). Some enhance its activity (right of the dashed line), for example, by reversing the existing mismatch. **c**, Inverse CDF of the change in predicted detection activity after simulating substitutions, summarized across 1,000 random sites in the SARS-CoV-2 genome; **b** shows one such site. At each of these 1,000 sites, we simulate 10,000 target sequences according to our substitution model and construct a distribution of the change in the probe’s predicted detection activity compared to its activity in detecting the original sequence. As in **b**, at each site the probe is complementary to the original target sequence, except with one random mismatch. Plotted is the median change taken across sites, as well as the 95^th^ and 5^th^ percentiles. The faster the curve rises to 0, the less likely there is to be a drop in activity. That the 5^th^ percentile curve shows a sharp drop for low values (∼<0.1) on the horizontal axis indicates that some sites may experience a pronounced drop in detection activity over time, but that even for these sites it is unlikely (∼10% chance). **d**, Effect of simulating substitutions on the ordering of ADAPT’s designs. We begin with the top 20 design options output by ADAPT for targeting SARS-CoV-2 genomes and, for this analysis, consider only the probe (Cas13a guide) from each design option. Each point represents one of the 20 probes. We rank the probes according to their mean predicted detection activity across the genomes; this ranking is on the horizontal axis. Then, for each genome, we simulate 10,000 sequences according to our substitution model (at the site where a probe binds) and compute the 5^th^ percentile of the predicted detection activities between the probe and these simulated sequences. We rank the probes accounting for simulated substitutions (vertical axis) according to the mean of this 5^th^ percentile value taken across the genomes. In this analysis, we use only 500 randomly sampled genomes from the set of genomes used to design the 20 probes with ADAPT, in order to reduce runtime.

For some viruses there are few genome sequences, especially early in an outbreak, and therefore little data on their variation. We developed a scheme that uses the general time-reversible (GTR) nucleotide substitution model^44^ to forecast likely genome substitutions in the region a probe detects, allowing us to estimate a probability that a probe’s activity will drop over time (Supplementary Note 5 and Extended Data Fig. 8a). A drop may result from mutations at mismatch-sensitive sites or at other sites within or around the binding region. Applied to severe acute respiratory syndrome coronavirus 2 (SARS-CoV-2), we found that, for some Cas13a designs, there is a low probability (~10%) their predicted activity will drop over 5 years (Extended Data Fig. 8b,c). This forecasting may help in risk-averse situations, but it has only a minor effect overall on assay rankings (Extended Data Fig. 8d).

We computationally evaluated ADAPT’s output on seven RNA viruses with differing degrees of diversity. Precise outputs are affected by algorithmic randomness, but are generally consistent in targeting the same genomic regions (Supplementary Fig. 18). Cross-validation confirms that ADAPT’s designs generalize to unobserved data: designs are predicted to detect >85% of held-out genomes for all seven species and exhibit, in all but one species (Rhinovirus A), ‘high activity’ (defined as top 25% of our dataset’s activities) in detecting the majority of held-out genomes (Fig. 4b). Relaxed design criteria, which permit more complex assays (Methods), achieve an even higher sensitivity, with designs predicted to detect >96% of held-out genomes for all seven species (Supplementary Fig. 19). Thus, ADAPT’s outputs are robust across different viruses.

We applied ADAPT to design species-specific assays, including amplification primers and Cas13a guides, for the 1,933 viral species known to infect vertebrates. The designs have short amplicons and use 1–3 guides for all species (Fig. 4c and Supplementary Fig. 20). Thus, the assays are practical. For 95% of species, the guides detect the majority of known genomes with high predicted activity (Fig. 4d; for 88% of species, they detect >95% with high activity).

Our assays—designed to comprehensively detect species-level diversity—could detect novel viruses that are nested within known species. We simulated the design of assays in 2018 for detecting the SARS-related coronavirus (SARS-related CoV) species and then evaluated their detection of SARS-CoV-2, a lineage of the species that did not emerge until a year later. ADAPT’s second-highest-ranked design is predicted to detect SARS-CoV-2 well, while other designs are predicted to exhibit weak or no detection (Supplementary Fig. 21a,b). Detection is facilitated by bat SARS-like viral genomes, similar to SARS-CoV-2, that were available in 2018. Nevertheless, heavy sampling biases hinder the designs’ efficacies: in 2018, SARS-CoV-1 was overrepresented in the species (85%) relative to bat SARS-like viruses, and its divergence from SARS-CoV-2 weakens detection of the novel virus. If we downweigh consideration to SARS-CoV-1 (Methods), four of ADAPT’s five highest-ranked 2018 assays are predicted to detect SARS-CoV-2 well (Supplementary Fig. 21c,d). Such broadly effective assays constitute a proactive toolkit for detection.

We examined the computational requirements of designing assays for 1,933 species. ADAPT’s end-to-end design completed quickly: under 2 h for 80% of species, under 24 h for all but three species (human cytomegalovirus, SARS-related CoV and FLUAV) and under 38 h for all (Fig. 4e). Details on memory usage and other design considerations are in Supplementary Figs. 20c–f and 22 and Supplementary Note 4d.

### Experimental evaluation of ADAPT’s designs

**Fig. 5:**
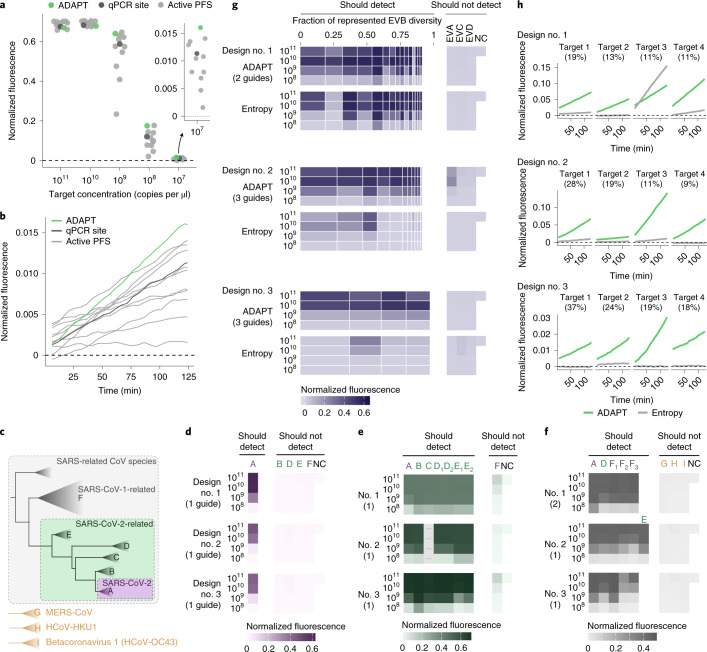
Sensitive and specific detection with ADAPT’s designs. **a**, Fluorescence at varying target concentrations in the US CDC’s SARS-CoV-2 N1 RT–qPCR amplicon. Compared Cas13 guides are: ADAPT’s guide (green); a guide with an active (non-G) PFS at the site of the N1 qPCR probe (dark gray); and ten randomly selected guides with an active PFS (light gray). All are constrained to the amplicon. **b**, Fluorescence over time for guides in **a** at target concentration of 10^7^ copies per µl. Panel **a** shows the final time point. **c**, Phylogeny within the SARS-related CoV species based on ref. ^47^, and three other betacoronaviruses. **d**, Fluorescence for ADAPT’s top-ranked SARS-CoV-2 designs in detecting representative targets of the clades in **c**. Rankings, from 1 to 3, follow ADAPT’s predicted performance. The label to the left of each row indicates target concentration (copies per µl). NC, no template control. Colors refer to **c**. Parentheticals indicate the number of Cas13 guides in ADAPT’s design. **e**, Same as **d**, but for SARS-CoV-2-related. The empty column indicates the sequence in the amplicon has high ambiguity and was not tested. Clades D and E sometimes have two representative targets in an amplicon; when two target labels have one representative sequence, the same value is plotted under each label. **f**, Same as **d**, but for SARS-related CoV. F_1_ is SARS-CoV-1, and F_2_ and F_3_ are related bat SARS-like CoVs. E requires a separate representative target in only one amplicon. G, H and I are defined in **c**. **g**, Fluorescence for ADAPT’s top-ranked EVB designs in detecting EVB and representative targets for Enterovirus A/C/D (EVA/C/D). Each band is an EVB target having width proportional to the fraction of EVB genomic diversity represented, within the amplicon of ADAPT’s design. Under each ADAPT design is one baseline guide (‘Entropy’) from the site in the amplicon with an active PFS and minimal Shannon entropy. **h**, Fluorescence over time at target concentration of 10^8^ copies per µl, for the four EVB targets encompassing the largest fraction of genomic diversity. Panel **g** shows the middle time point. In all panels, fluorescence is reference normalized and background subtracted (Methods).

**Extended Data Fig. 9:**
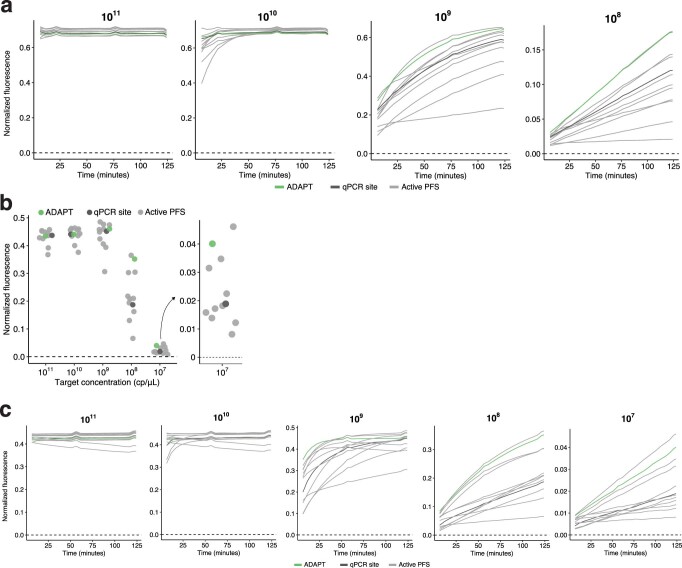
Benchmarking ADAPT’s designs in US CDC SARS-CoV-2 amplicons. **a**, Fluorescence over time for Cas13 guides at varying target concentrations (top of each plot, in cp/μL) within the US CDC’s SARS-CoV-2 N1 RT-qPCR amplicon. Compared guides are ADAPT’s design (green), a guide with an active (non-G) PFS at the site of the qPCR probe from the N1 assay (dark gray), and 10 randomly selected guides with an active PFS (light gray). Target concentration of 10^7^ cp/μL is shown in Fig. 5b. **b**, Fluorescence for Cas13 guides at varying target concentrations within the US CDC’s SARS-CoV-2 N2 RT-qPCR amplicon. The final time point is shown (124 minutes). Guides are as in **a**. **c**, Fluorescence over time for Cas13 guides at varying target concentrations (top of each plot, in cp/μL) within the US CDC’s SARS-CoV-2 N2 RT-qPCR amplicon. Guides are as in **a**.

We experimentally benchmarked our approach. We first considered the United States Centers for Disease Control and Prevention’s (US CDC’s) SARS-CoV-2 RT–qPCR diagnostic amplicons, a target of both RT–qPCR and CRISPR-based assays. As baselines in the N1 amplicon, we selected a Cas13a guide at the site of the qPCR probe and ten random guides in the amplicon, all having an active (non-G) PFS; selecting guides according to the PFS is the canonical design strategy, and the distribution of their activity in this amplicon is a benchmark representing previous strategies for CRISPR-based SARS-CoV-2 diagnostics^45,46^. The guide designed by our approach exhibits greater and faster-growing fluorescence at low target concentrations than all 11 of the baseline guides (Fig. 5a,b and Extended Data Fig. 9a). We observed similar results using the N2 amplicon (Extended Data Fig. 9b,c). Background activity does not impact these comparisons because all guides exhibit similarly low no-template fluorescence (Supplementary Fig. 23). These findings indicate that our designs permit better sensitivity against a known target sequence than the canonical approach focused on the PFS.

Next, we validated the comprehensiveness and specificity offered by our approach by considering taxa comprising the SARS-related CoV species (Fig. 5c). We tested ADAPT’s designs against representative targets within each taxon, which we identified systematically according to sequence composition (Methods). Our testing directly measures the fluorescent signal yielded by the Cas13a guides at varying target concentrations. We started with precise targeting of SARS-CoV-2. Using ADAPT, we generated lineage-specific designs for detecting SARS-CoV-2 that should not detect bat or pangolin SARS-like coronaviruses, including the RaTG13 genome (96% identity to SARS-CoV-2^47^), nor SARS-CoV-1 and other coronaviruses (Fig. 5c). All three of our approach’s best design options (ranked by predicted activity) detect SARS-CoV-2 with complete specificity: we observed no fluorescent signal for the related lineages (Fig. 5d).

We then broadened the targeted space within SARS-related CoV. We designed assays for the SARS-CoV-2-related lineage^48^, which consists of SARS-CoV-2 and its related bat and pangolin CoVs. Our approach’s three top-ranked designs sensitively and specifically detect all representative targets within SARS-CoV-2-related (Fig. 5e). Unlike the SARS-CoV-2 designs, we observed low off-target SARS-CoV-1 signal because the added comprehensiveness antagonizes specificity; this is unlikely to affect diagnostic results that use an adequate signal threshold for detection. We also designed species-specific assays for the full SARS-related CoV species, and all three top-ranked designs detect all representative targets within the species without any signal for three other betacoronaviruses (Fig. 5f).

**Extended Data Fig. 10:**
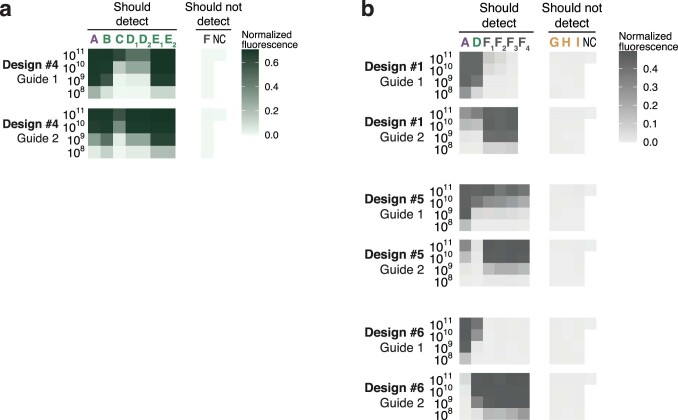
Separate guides for multi-guide designs in SARS-related CoV taxa. Fluorescence for ADAPT’s design options that use more than one Cas13 guide, separated by guide. Left label indicates target concentration (cp/μL). **a**, The two guides in Design #4 to detect the SARS-CoV-2–related taxon (Supplementary Fig. 24b). **b**, The two guides in Design #1 (Fig. 5f) and in Designs #5 and #6 (Supplementary Fig. 24c) to detect the SARS-related coronavirus species. In other figures, plotted value is the maximum across multiple guides. NC, no template control.

Overall, all top-ranked designs perform as desired across the SARS-related CoV taxa. For each taxon, ADAPT also generated additional design options (25 total) that generally exhibit the desired activity (Supplementary Fig. 24). Four of the designs use two Cas13a guides and, in these cases, our combinatorial optimization algorithm selects guides that detect distinct lineage groupings to maximize their collective sensitivity for the taxon (Extended Data Fig. 10).

We also evaluated limits of detection across extensive genomic variation. We focused on enteroviruses, which are estimated to cause millions of symptomatic infections yearly and frequent outbreaks^49,50^. Testing increasingly relies on pan-enterovirus RT–qPCR by targeting a highly conserved region, which has clinical value but limited surveillance utility^51^; species-specific assays would provide higher resolution than pan-enterovirus assays, and thus could aid surveillance.

We applied ADAPT to design species-specific assays for Enterovirus B (EVB), which is widespread^52^ and exceptionally diverse, with 63 known types^50^. ADAPT’s three top-ranked designs detect the spectrum of genomic variation with specificity for EVB, as desired (Fig. 5g). To benchmark our approach, we targeted conserved sites by designing a guide within each ADAPT-selected amplicon at the site with minimal Shannon entropy and an active PFS (Methods). Targeting conserved sites is a standard, widely used strategy for managing sequence diversity: conserved sites are a target of CRISPR-based diagnostics^3,53^ for diverse viruses and, in particular, entropy commonly steers the design of qPCR assays^12,54,55^. The entropy-based strategy fails to detect many targets representative of EVB’s genomic diversity (Fig. 5g). By contrast, our approach provides a higher fluorescent signal in nearly all representative targets, enabling a lower limit of detection in about half of them (Fig. 5h and Supplementary Fig. 25a–c). In many design options that we tested below the top three, the entropy-based strategy is more sensitive than our approach; however, in these cases the entropy-based strategy lacks species-specificity (Supplementary Fig. 26). Though ADAPT’s designs incorporate 1–3 guides and the entropy-based strategy uses one, we tested multiple entropy-based guides in five designs and found they exhibit similar activity at low target concentrations (Supplementary Figs. 25d–f and 27). Our results indicate that model-based optimization enables diagnostics that sensitively detect vast genomic diversity, including with improved sensitivity over a conservation-based strategy.

To further evaluate specificity in clinically relevant conditions, we compared, in silico, all experimentally tested guides with the human transcriptome and 11 common bacterial pathogen genomes (Methods). All ADAPT-designed guides are at least five mismatches different from human transcripts and these bacterial genomes, indicating they are unlikely to exhibit off-target effects (Extended Data Fig. 6b).

## Discussion

We developed an approach that combines a deep learning model with combinatorial optimization to design viral diagnostics. We applied our approach using CRISPR-based diagnostics, for which we generated a dataset on diagnostic signal and learned a model that predicts enzymatic activity during detection. Our approach integrates viral variation into an objective function to create designs that are maximally active across variants. Alongside achieving comprehensive sensitivity, the approach enforces specificity at any taxonomic level, so its outputs can be used in clinical assays to specifically detect viruses, including related subspecies. We built ADAPT, which runs our approach at scale.

We experimentally validated ADAPT’s designs across extensive target variation. ADAPT’s designs (1) exhibit higher fluorescence for SARS-CoV-2 at low concentrations than designs from previous strategies; (2) are sensitive and specific to the lineage level across closely related taxa; and (3) specifically identify a diverse species, EVB, with lower limits of detection across its genomic diversity, compared with a strategy focused on sequence conservation. While we tested extensively across viral variation, we used synthetic targets. Validation on patient and environmental samples would be important before deploying ADAPT’s assays in practice, although previous studies^8,53,56^ have demonstrated that they work well on such samples.

Days after SARS-CoV-2 was first sequenced, we applied an early version of ADAPT to design CRISPR-based assays for SARS-CoV-2 and other respiratory viruses^57^. Though designed from only the 20 genomes available then^58^, we predict this SARS-CoV-2 assay to detect 99.8% of the ~5.2 million genomes sequenced through December 2021. Early versions of ADAPT also designed assays for 169 human viruses and influenza subtyping^8^, and for Lassa virus^53^.

We envision running ADAPT regularly for thousands of viruses, so that designs continually reflect evolution. That will provide broadly effective designs in advance of an outbreak. ADAPT’s designs perform well for known viruses and can even be useful for novel viruses not yet known during the design. For novel viruses, however, genome sampling biases can hinder ADAPT’s performance. Our simulation of this application—in which we designed for SARS-related CoV before SARS-CoV-2’s emergence—motivates preparing several highly ranked assays rather than one, and having them ready to test on a novel virus. Relatedly, ADAPT’s assays could struggle for viruses with few sequences in genome databases; ADAPT’s forecasting of genome substitutions and their impact on designs (Supplementary Note 5 and Extended Data Fig. 8) may help, but is limited to the sequence space around known genomes.

There is room for methodological improvements in ADAPT. Integrating a learned model for amplification primers, rather than using conventional heuristics, could improve amplification steps of CRISPR-based diagnostics. However, such a model would require constructing a training dataset and recent developments in amplification-free CRISPR-based detection^46,59,60^ may negate motivation for such work. Another area is algorithmic. For instance, rather than maximizing detection over a uniform distribution of genomes, an approach to weigh genomes could correct for sampling biases and improve the chances that ADAPT’s designs detect undersampled emerging and novel viruses.

Our Cas13a dataset and modeling could illuminate guide design principles. While we extracted design considerations from linear models, a more thorough modeling of predefined features—similar to that performed for Cas13d^18^—may reveal additional Cas13a principles. Our CNN models may also learn novel features, and interpreting these models^61^ could identify elements of the input sequences that promote high activity and underlie new design principles.

Though we trained a deep neural network for CRISPR–Cas13a, ADAPT accommodates models for other nucleic acid technologies. An example is qPCR. SARS-CoV-2 qPCR assays exhibit variability in their reported sensitivities^62^ and many target regions that have acquired mutations^32,33,63^, motivating a learned model and an application of ADAPT.

Beyond viral diagnostics, our approach could benefit other tasks that require maximally active sequences across genomic variation. As examples, variation impacts short interfering RNA^64^, antibody^65^ and CRISPR-based antiviral^66^ therapeutic efficacy. With appropriate models, model-based optimization could also enhance sequence-based vaccine selection^67–69^ by designing vaccine antigens that drive immune responses to be maximally active across viral diversity.

ADAPT’s web frontend is available at https://adapt.run. This resource, which includes annotated design visualizations, also provides pre-made designs against vertebrate-associated viruses. Using ADAPT, those proactive designs can continually update to reflect recent variation. ADAPT is also available as a software package at https://github.com/broadinstitute/adapt.

Our approach, together with the introduction of ADAPT, improves the development and efficacy of viral diagnostics, and has the potential to do so for other sequence-based technologies.

## Methods

### ADAPT

Supplementary Notes describe ADAPT’s algorithms, data structures and implementation details. Supplementary Note 2 defines objective functions and describes how ADAPT optimizes them. Supplementary Note 3 describes how ADAPT enforces specificity. Supplementary Note 4 describes how ADAPT searches for genomic regions to target and links with sequence databases. Supplementary Note 5 describes how ADAPT forecasts relatively likely genome substitutions.

### Introductory analyses

To illustrate viral database growth, we charted the growth in the number of viral genomes and their unique 31-mers over time (Supplementary Fig. 1). We first curated a list of viral species known to infect humans from a National Center for Biotechnology Information (NCBI) database^70^ (November 2019). For each, we took all NCBI genome neighbors^31^ (influenza sequences from the Influenza Virus resource^71^), which represent near-complete or complete genomes. To assign a date for each, we used the GenBank entry creation date rather than sample collection date for several reasons, including that this date more directly represents our focus in the analysis (when the sequence becomes present in the database) and that every entry on GenBank contains a value for this field. To control for some viruses having multiple segments (and thus sequences), we only used counts for one segment for each species, namely the segment that has the greatest number of sequences.

We used FLUAV subtyping as an example to demonstrate the effect of evolution on diagnostics (Extended Data Fig. 1a and Supplementary Fig. 2). We selected the most conserved *k*-mers—representing probe or guide sequences—from the sequences available at different years. Here, for simplicity, we ignored all other constraints, such as detection activity and specificity (the latter of which is critical for subtyping), which may further degrade the temporal performance of the selected *k*-mers. In particular, for each design year *Y*, we selected the 15 non-overlapping 30-mers found in the largest number of sequences taken from the two most recent years (*Y* − 1 and *Y*). We then measured the fraction of sequences in subsequent test years (*Y*, *Y* + 1, …) that exactly contain each of these *k*-mers. We performed the design strategy over ten resamplings of the sequences and use the mean fraction. We repeated this four times: for segment 4 (HA) sequences of H1 and H3 subtypes, and segment 6 (NA) sequences of N1 and N2 subtypes.

To visualize mutations accumulating on a genome during the course of an outbreak (Extended Data Fig. 1b), we used complete SARS-CoV-2 genomes from Global Initiative on Sharing All Influenza Data (GISAID)^58^. We called variants in all genomes, through 2020, against the reference genome ‘hCoV-19/Wuhan/IVDC-HB-01/2019’ (GISAID accession ‘EPI_ISL_402119’). For every date *d* between 1 February 2020 and 1 January 2021, spaced apart by 1 month, at every position we calculated the fraction of all genomes collected up to *d* that have a variant against the reference. We called all variants present between 0.1% and 1% frequency on some *d* as ‘low’ frequency and variants at ≥1% frequency on some *d* as ‘high’ frequency. We ignored all variants present at ≥1% frequency on the initial *d* (ancestral) or that were both low frequency on the initial *d* and stayed low frequency by the final *d*—that is, we kept the variants that transitioned to low or high frequency by the final *d*. We show the *d* when the variant first becomes called as low (light purple) or high (dark purple) frequency. If a variant transitions both to low and then to high frequency by the final *d*, we only show it for the *d* when it becomes high frequency.

### Cas13a library design and testing

We designed a collection of CRISPR–Cas13a CRISPR RNA (crRNA) guides and target molecules to evaluate guide–target activity, focusing on assessing likely active guide–target pairs. First, we designed a target (the wild-type target) that is 865 nucleotides (nt) long (design details for the wild-type target are in the subsequent paragraph). We then created 94 guides (namely, the 28-nt spacers) tiling this wild-type target (Fig. 1a and Supplementary Fig. 3a). In the tiling scheme there are 30-nt blocks, each having four overlapping guides, in which the starts of the three guides, from the start of the most 5′ guide, are 4 nt, 13 nt and 23 nt. Of the 94 guides, 87 are experimental, three are negative controls and four are positive controls. We created 229 unique target sequences: one of them is the wild-type sequence (guides should exhibit activity against this target), 225 are experimental (mismatches and varying PFS alleles against the guides) and three are negative controls. All guides exactly match the wild-type target and should detect this, except the three negative control guides, which are not intended to detect any targets except one of the three negative control targets each. The four positive control guides target four 30-nt regions with a perfectly complementary sequence and non-G PFS that are held constant across all targets, with the exception of the three negative control targets. Across the experimental targets, the mismatches profile varying choices of positions and alleles against the guide. For the experimental targets, we generated single mismatches evenly spaced every 30 nt along the experimental region such that every guide targeting this region has either a single mismatch or an altered PFS at +1 or +2 nt from the protospacer; we created a total of 45 (3 × 15) such targets to probe all three possible mismatch alleles and 15 of 30 of the possible phasings. In the remainder of the experimental targets, we generated targets with two, three or four mismatches per 30-nt block with respect to the guide RNA in phase with the block. For these targets, we randomly selected mismatch positions to uniformly sample (or, when possible, exhaustively enumerate) average mismatch spacing and average mismatch distance to the center of the spacer, and randomly selected mismatch alleles. The 87 experimental guides may detect up to 226 unique target sequences (the wild type and 225 experimental targets), providing 19,662 experimental guide–target pairs.

To construct the wild-type target sequence, we aimed to produce a composition spanning viral genomic sequence diversity. In particular, we started with a previously described dataset of genomes from human-infecting viral species^72^, constructed a vector of the dinucleotide frequencies for each species and performed principal component analysis of the species from these vectors. For each 30-nt block of the wild-type target, we selected a point from the space of the first three principal components (uniformly at random), reconstructed a corresponding vector of dinucleotide frequencies (that is, transformed the point back to the original space) and then iteratively selected every next nucleotide in the block according to the distribution of dinucleotides. A goal of this scheme is for dinucleotides that are variable across viral species to also vary in frequency across the wild-type target: a dinucleotide that explains considerable variance across viral species (for example, is rich in some viral species and poor in others) ought to be rich in some blocks of the wild-type target and poor in other blocks, whereas a dinucleotide that explains little variance across species ought to have similar frequency along the target. In positions that would serve as a PFS for a guide, we disallowed G, and proportionately adjusted upwards the probability of choosing a G in non-PFS positions to maintain the total dinucleotide frequency in accordance with the randomly selected distribution (mismatches in experimental targets can still introduce a G PFS).

We synthesized the targets as DNA, in vitro transcribed them to RNA and synthesized the crRNAs as RNA. On all crRNAs, we used the same direct repeat (‘GAUUUAGACUACCCCAAAAACGAAGGGGACUAAAAC’). To determine a reasonable concentration for measuring fluorescence over time points, we tested eight concentrations of two targets and two guides in a pilot experiment (Supplementary Fig. 4a) and proceeded with 6.25 × 10^9^ copies per µl. We tested the library using CARMEN; we followed the methodology described in ref. ^8^, which also contains the protocol. Briefly, a guide–target pair is enclosed in a droplet, together with the Cas13a enzyme, that may result in a detection reaction and thus fluorescence. We took an image of each location on each chip roughly every 20 min to measure this fluorescence. To alleviate the presence of microdroplets in this experiment (that is, an irregular pairing of target and guide; about one-third of the droplets), we trained and applied a CNN on hand-labeled data to identify and remove these.

### Quantifying activity

In our Cas13a detection experiments, a fluorescent reporter is cleaved over time and its cleavage follows first-order kinetics:

$$\begin{array}{l}\frac{{{\mathrm{d}}\left[ R \right]}}{{{\mathrm{d}}t}} = - \frac{{k_{{\mathrm{cat}}}}}{{K_{\mathrm{M}}}}\left[ E \right]\left[ R \right]\\ \Rightarrow \left[ R \right] = [R]_0{\mathrm{e}}^{ - \frac{{k_{{\mathrm{cat}}}}}{{K_{\mathrm{M}}}}\left[ E \right]t}\end{array}$$

where [*R*] is the concentration of the not-yet-cleaved reporter, [*E*] is the concentration of the Cas13a guide–target complex, $\frac{{k_{{\mathrm{cat}}}}}{{K_{\mathrm{M}}}}$ is the catalytic efficiency of the particular guide–target complex and *t* is time. The fluorescence measurements that we make, *y*, are proportional to the quantity of cleaved reporter at some time point:

$$y \propto [R]_0 - \left[ R \right].$$

Therefore, for each guide–target complex we fit a curve of the form

$$y = C\left( {1 - {\mathrm{e}}^{ - kt}} \right) + B.$$

Here, *C* and *B* represent the saturation point and background fluorescence, respectively. *k* represents the rate at which the reporter is cleaved, and it is proportional to the catalytic efficiency of the particular guide–target complex:

$$k = \frac{{k_{{\mathrm{cat}}}}}{{K_{\mathrm{M}}}}\left[ E \right].$$

This relationship is validated by the linear relationship between *k* and [*E*] (Supplementary Fig. 4a) when we vary the concentration of target (the limiting component of the complex). In producing our dataset, we held [*E*] constant. We used log_10_(*k*) as our measurement of the overall enzymatic activity resulting from the guide–target pair (Figs. 1 and 2 and Supplementary Fig. 4a,b). Intuitively, each step-increase in log_10_(*k*) corresponds to a fold-decrease in the half-life of the reporter in the reaction.

Our experimental data incorporate multiple droplets for each guide–target pair (Extended Data Fig. 2a). Each droplet represents one technical replicate of a particular guide–target pair. Thus, we have fluorescence values for each replicate at different time points, and in practice we compute the activity log_10_(*k*) for each replicate.

We curated the data to obtain a final dataset. Namely, we discarded data from two guides that showed no activity between them and any targets, owing to low concentrations in their synthesis. We also did not use data from positive or negative control guides, or from the negative control targets. Our final dataset contains 19,209 unique guide–target pairs (Supplementary Fig. 3b,c), counting 20 nt of sequence context around each protospacer in the target (18,253 unique pairs when not counting context).

Most guide–target pairs show activity (Extended Data Fig. 2d), as expected. At small values of *k* on a limited time scale (*t* up to ~120 min), we do not observe reporter activation (Supplementary Fig. 4b). Moreover, the curve becomes approximately linear (first-order Maclaurin expansion: *y* ≈ *Ckt* + *B*). At such values of *k*, we cannot estimate both *C* and *k* together; intuitively, this is because there is too little detectable signal. Therefore, there is a cutoff at which we can estimate *k*; we labeled activities at log(*k*) *>* −4 as active, and the others as inactive. This phenomenon also implies that at smaller values of *k*, including ones we label as active, activity estimates might be less reliable.

### Predicting detection activity

#### Measurement error

To account for measurement error, we sampled, with replacement, ten technical replicate measurements of activity for each guide–target pair (Extended Data Fig. 2a). We used this strategy to ensure that, although there are differing numbers of replicates per guide–target pair, each pair would be represented in the dataset with the same number of replicates. There are 19,209 × 10 = 192,090 points in total in our dataset that we use for training and testing. When plotting regression results on guide–target pairs in the hold-out test set (Fig. 2c, Extended Data Fig. 4a and Supplementary Fig. 10), we set the true activity of a pair to be the mean of the measured activities across the technical replicates for the pair.

#### Model and input descriptions

We approached prediction using a two-step hurdle model, reasoning that (1) separate processes govern whether a guide–target pair is active compared with the level of its activity; and (2) we could better predict the activity of active pairs if we excluded the inactive pairs from a regression. We developed a classifier to decide whether a pair is inactive or active, and a regression model to predict the activity of only active pairs.

We explored multiple models for classification (Fig. 2a and Supplementary Fig. 5a), each with a space of hyperparameters:

- L1 logistic regression: regularization strength (logarithmic in [10^−4^, 10^4^])
- L2 logistic regression: regularization strength (logarithmic in [10^−4^, 10^4^])
- L1 + L2 logistic regression (elastic net): regularization strength (logarithmic in [10^−4^, 10^4^]), L1/L2 mixing ratio (1.0 − 2^*x*^ + 2^−5^ for *x* uniform in [−5, 0])
- Gradient-boosted trees (GBT): learning rate (logarithmic in [10^−2^, 1]), number of trees (logarithmic in [1, 2^8^], integral), minimum number of samples for splitting a node (logarithmic in [2, 2^3^], integral), minimum number of samples at a leaf node (logarithmic in [1, 2^2^], integral), maximum depth of a tree (logarithmic in [2, 2^3^], integral), number of features to consider when splitting a node (for *n* features, chosen uniformly among considering all, 0.1*n*, $\sqrt n$ and log_2_ *n*)
- Random forest (RF): number of trees (logarithmic in [1, 2^8^], integral), minimum number of samples for splitting a node (logarithmic in [2, 2^3^], integral), minimum number of samples at a leaf node (logarithmic in [1, 2^2^], integral), maximum depth of a tree (chosen uniformly among not restricting the depth or restricting the depth to a value picked logarithmically from [2, 2^4^] and made integral), number of features to consider when splitting a node (for *n* features, chosen uniformly among considering all, 0.1*n*, $\sqrt n$ and log_2_ *n*)
- Support vector machine (SVM; linear): regularization strength (logarithmic in [10^−8^, 10^8^]), penalty type (chosen uniformly among L1 and L2)
- Multilayer perceptron (MLP): number of layers excluding the output layer (uniform in [1, 3]), dimensionality of each layer excluding the output layer (each chosen uniformly in [4, 127]), dropout rate in front of each layer (uniform in [0, 0.5]), activation function (chosen uniformly among rectified linear unit (ReLU) and exponential linear unit (ELU)), batch size always 16
- Long short-term memory recurrent neural network (LSTM): dimensionality of the output vector (logarithmic in [2, 2^8^], integral), whether to be bidirectional (chosen uniformly among unidirectional and bidirectional), dropout rate in front of the final layer (uniform in [0, 0.5]), whether to perform an embedding of the one-hot encoded nucleotides and the dimensionality if so (chosen with 1/3 chance to not perform an embedding, and with 2/3 chance to perform an embedding with dimensionality chosen uniformly in [1, 8]), batch size is always 16
- CNN: number of parallel convolutional filters and their widths (chosen uniformly among not having a convolutional layer, 1 filter of width 1, 1 filter of width 2, 1 filter of width 3, 1 filter of width 4, 2 filters of widths {1, 2}, 3 filters of widths {1, 2, 3} and 4 filters of widths {1, 2, 3, 4}), convolutional dimension (uniform in [10, 249]), pooling layer width (uniform in [1, 3]), pooling layer computation (chosen uniformly among maximum, average and both), number of parallel locally connected layers and their widths (chosen uniformly among not having a locally connected layer, 1 filter of width 1, 1 filter of width 2 and 2 filters of widths {1, 2}), locally connected filter dimension (uniform in [1, 4]), number of fully connected layers and their dimensions (chosen uniformly among 1 layer with dimension uniform in [25, 74] and 2 layers each with dimension uniform in [25, 74]), whether to perform batch normalization in between the convolutional and pooling layers (uniform among yes and no), activation function (chosen uniformly among ReLU and ELU), dropout rate in front of the fully connected layers (uniform in [0, 0.5]), L2 regularization coefficient (lognormal with mean *µ* = −13, *σ* = 4), batch size (uniform in [32, 255]), learning rate (logarithmic in [10^−6^, 10^−1^])

Similarly, for regression we explored multiple models (Supplementary Fig. 5b,c), each with a space of hyperparameters:

- L1 linear regression: regularization strength (logarithmic in [10^−8^, 10^8^])
- L2 linear regression: regularization strength (logarithmic in [10^−8^, 10^8^])
- L1 + L2 linear regression (elastic net): regularization strength (logarithmic in [10^−8^, 10^8^]), L1/L2 mixing ratio (1.0 − 2^*x*^ + 2^−5^ for *x* uniform in [−5, 0])
- GBT: same hyperparameter space as for classification
- RF: same hyperparameter space as for classification
- MLP: same hyperparameter space as for classification
- LSTM: same hyperparameter space as for classification
- CNN: same hyperparameter space as for classification

Model selection and evaluation describes the search process.

When training and testing the models, we used a 28-nt guide and target sequence, and include 10 nt of context in the target sequence on each side of the protospacer. We tested the following different inputs:

- ‘One-hot (1D)’: vector containing 4 bits to encode the nucleotide at each target position and 4 bits similarly for each guide position; with a 28-nt guide and 10 nt of context in the target around the protospacer, there are (10 + 28 + 10 + 28) × 4 = 304 bits
- ‘One-hot MM’: similar to ‘One-hot (1D)’ except explicitly encoding mismatches between the guide and target—that is, vector containing 4 bits to encode the nucleotide at each target position and 4 bits, at each guide position, encoding whether there is a mismatch (if not, all 0) and, if so, the guide allele; same length as ‘One-hot (1D)’
- ‘Handcrafted’: features are count of each nucleotide in the guide, count of each dinucleotide in the guide, GC count in the guide, total number of mismatches between the guide and target sequence, and a one-hot encoding of the 2-nt PFS (coupling the 2 nucleotides); the number of features is 4 + 16 + 1 + 1 + 16 = 38
- ‘One-hot MM + Handcrafted’: concatenation of features from ‘One-hot MM’ and ‘Handcrafted’, except removing from ‘One-hot MM’ the bits encoding the 2-nt PFS because these are included in ‘Handcrafted’

We used these inputs for all models except the LSTM and CNN. For these two models, which can capture and extract spatial relationships in the input, we used an alternative input (labeled ‘One-hot (2D)’ in figures). Here, the input dimensionality is (48, 8) and consists of a concatenated one-hot encoding of the target and guide sequence. Namely, each element *x*_*i*_ (*i* ∈ {1*…* 48}) is a vector [*x*_*i*,*t*_, *x*_*i*,*g*_]. Target context corresponds to *i* ∈ {1*…* 10} (5′ end) and *i* ∈ {39*…* 48} (3′ end); for these *i*, *x*_*i*,*t*_ is a one-hot encoding of the target sequence and *x*_*i*,*g*_ is all 0. The guide binds to the target at *i* ∈ {11*…* 38} and, for these *i*, *x*_*i*,*t*_ is a one-hot encoding of the target sequence protospacer at position *i* − 10 of where the guide is designed to bind, while *x*_*i*,*g*_ is a one-hot encoding of the guide at position *i* − 10.

We evaluated all models, except the MLP, LSTM and CNN, using scikit-learn 0.22 (ref. ^73^). We implemented and evaluated the MLP, LSTM and CNN models in TensorFlow 2.1.0 (ref. ^74^).

For the MLP, LSTM and CNN models, we used binary cross-entropy as the loss function for classification and mean squared error for regression. For these three models, we used the Adam optimizer^75^ and performed early stopping during training (maximum of 1,000 epochs) with a held-out portion of the training data. Additionally, for the CNN we regularized the weights (L2). When training all classification models, we weighted the active and inactive classes equally.

#### Data splits and test set

When performing model cross-validation, we must determine folds of the data. Guides are tiled along the 865-nt wild-type target (Fig. 1a and Supplementary Fig. 3a) and their positions along the RNA target enable dividing guide–target pairs into two sets in which each set consists of cognate guide–target pairs that are unrelated to the pairs in the other set. During *k*-fold cross-validation, we split the positions of the guide–target pairs into *k* consecutive folds (positions are ordered, that is, not shuffled). For each fold, the validation set consists of guide–target pairs where the guide’s position is from the validation range, and the training set consists of guide–target pairs where the guide’s position is from the position ranges in the remaining *k* − 1 folds. Note that the validation set consists of guide–target pairs from one contiguous region of the 865-nt RNA targets, while the training set is not necessarily contiguous. With this strategy alone, guides between the training and validation sets may overlap according to the position against which they were designed along the wild-type target. Although effects on activity might be position-dependent within the guide, this overlap can cause guides to have similar sequence composition or to be in regions of the target sequence with similar structure. To remove this possibility of leakage between a data split, after making a split of *X* into *X*_train_ and *X*_validate_, we removed all guide–target pairs from *X*_validate_ for which the guide has any overlap, in target sequence it is designed to detect, with a guide in *X*_train_. We performed this data splitting strategy during all cross-validated analyses, including for determining outer and inner folds of nested cross-validation.

We also followed this strategy to choose a test set that we hold out from all analyses and use only for evaluating the final CNNs. This test set consists of the 30% of all guides (counted before removing overlaps between the test set and other data) that detect the 3′ end of the 865-nt targets.

#### Model selection and evaluation

We performed nested cross-validation to select models—both for classification and regression—and evaluate our selection of them (Fig. 2a and Supplementary Fig. 5). We used five outer folds of the data. For each outer fold, we searched for hyperparameters using a cross-validated (five inner folds) random search over the space defined in Model and input descriptions; we scored using the mean auROC (classification) or Spearman correlation (regression) over the inner folds. In each random search, we used 100 hyperparameter choices for all models, except for the LSTM and CNN models (50), which we found slower to train.

The CNN models outperformed others in the above analysis, so we selected a final CNN model for classification and another for regression. For each of classification and regression, we performed a random search across five folds of the data using 200 random samples. We selected the model with the highest auROC (classification) or Spearman correlation (regression) averaged over the folds. Our evaluations of these two models used the hold-out test set.

#### Incorporating into ADAPT

We integrated the CNN models into ADAPT. First, we set the decision threshold on the classifier’s output to be 0.577467. We chose the threshold, via cross-validation, to achieve a desired precision of 0.975. In particular, we took five folds of our data (excluding test data) and, for each fold, we calculated the threshold that achieves a precision of 0.975 on the validation data. Our decision threshold is the mean across the folds.

We then defined a piecewise function, incorporating the classification and regression models, as:

$$d( {p,s} ) = \left\{ {\begin{array}{*{20}{c}} {0,} & {{\mathrm{if}}\,C( {p,s} ) < t} \\ {{\mathrm{max}}\left( {0,r + R( {p,s} )} \right),} & {\mathrm{else}} \end{array}} \right.$$

where *d*(*p*, *s*) is the predicted detection activity between a probe *p* and target sequence *s* (*s* includes 10 nt of context). *C*(*p*, *s*) is the output of the classifier, *t* is the classification decision threshold and *R*(*p*, *s*) is the output of the regression model. *r* is a shift that we add to regression outputs to ensure *d*(*p*, *s*) is non-negative; though a nice property, it is not strictly needed as long as we constrain the ground set as described in Supplementary Note 2a. The choice of *r* should depend on the range of activity values in the dataset; here, *r* = 4.

#### Comparison of predictions with independent Cas13a datasets

Supplementary Note 1 describes how we evaluated our model’s predictions using independent Cas13a datasets from refs. ^36,37^. When reporting *P* values for Spearman’s test and Pearson’s test (Extended Data Fig. 5), the alternative hypothesis is that the true correlation is not 0 (Pearson’s test uses a *t*-distribution). Pearson’s *r* (Extended Data Fig. 5) was calculated as a sample correlation coefficient between our model’s predicted values and paired, independently measured values.

### ADAPT analyses

#### Comparing algorithms for submodular maximization

To compare the canonical greedy algorithm for constrained monotone submodular maximization^43^ with the fast randomized combinatorial algorithm^42^ (Supplementary Fig. 13), we ran ADAPT five times under each choice of parameter settings and species. For each run, we plotted the mean objective value taken across the best five design options. We used the arguments ‘-pm 3 -pp 0.9 --primer-gc-content-bounds 0.3 0.7 --max-primers-at-site 10 -gl 28 --max-target-len 250’ with our Cas13a activity model. We used the default objective function in ADAPT: 4 + *A* − 0.5 *P* − 0.25 *L*, where *A* is the objective value maximized by the submodular maximization algorithms, *P* is the number of primers and *L* is the target length.

#### Benchmarking comprehensiveness

To benchmark comprehensiveness (Fig. 3b,c and Supplementary Fig. 14), we ran ADAPT with three approaches. In all approaches, we decided that a probe detects a target sequence if and only if they are within one mismatch, counting G-U wobble pairs as matches, and used a sliding window of 200 nt and a probe length of 30 nt. We used bootstrapping to estimate uncertainty around plotted values owing to viral genome sampling: five times, we randomly sampled with replacement from all NCBI genome neighbors^31^ for each species (if there are *N* neighbors, we randomly sampled *N* with replacement) and used each of these resamplings as input to five runs. In the first approach (baselines), we used ADAPT’s design_naively.py program to select probes within each window via three strategies: (1) the consensus probe, computed at every site within the window, that detects the most number of genome sequences (‘consensus’); (2) the most common probe sequence, determined at every site within the window, that detects the most number of genome sequences (‘mode’); and (3) the *n* most common subsequences, with all *n* determined at each site in the window, choosing the *n* from the site where they collectively detect the most number of genome sequences (doing this separately for *n* ranging from 1 to 10). In the second approach, we maximized expected activity using ADAPT across the target sequences with different numbers of probes (hard constraints) using a penalty strength of 0 (that is, no soft constraint). Here, we defined the activity to be binary: 1 for detection, and 0 otherwise; this has the property that expected activity is equivalent to the fraction of sequences detected. In the third approach, we use the objective function in ADAPT that minimizes the number of probes subject to constraints on the fraction of sequences detected (specified via ‘-gp’; 0.9, 0.95 and 0.99).

#### Evaluating dispersion and generalization

We evaluated the dispersion, owing to randomness and sampling, in ADAPT’s designs (Supplementary Fig. 18). In all cases, we used all NCBI genome neighbors^31^ for each species and used the following arguments with ADAPT: ‘--obj maximize-activity --soft-guide-constraint 1 --hard-guide-constraint 5 --penalty-strength 0.25 -gl 28 -pl 30 -pm 3 -pp 0.98 --primer-gc-content-bounds 0.35 0.65 --max-primers-at-site 10 --max-target-length 500 --obj-fn-weights 0.50 0.25’, with a cluster threshold such that there is only one cluster, and used our Cas13a activity model. We ran ADAPT in two ways: 20 times without changing the input (output differences are owing to algorithmic randomness) and 20 times with resampled input (output differences are owing both to randomness and to sampling of the input sequences). Then, we measured dispersion by treating the five highest-ranked design options from each run as a set and computing pairwise Jaccard similarities across the 20 runs. This computation requires us to evaluate overlap between two sets: in one comparison, we consider a design option *x* to be in another set if *x* is present exactly in that other set (same primers and probes) and, in the other comparison, we consider a design option *x* to be in another set if that other set has some design option with both endpoints within 40 nt of *x*’s endpoints.

To evaluate the generalization of ADAPT’s designs (Fig. 4b), we performed cross-validation via repeated random subsampling. For each species, we took all NCBI genome neighbors^31^ and, 20 times, randomly selected 80% of them to use as input for design and the remaining 20% to test against. For each split, we used the same arguments with ADAPT as when evaluating dispersion: ‘--obj maximize-activity --soft-guide-constraint 1 --hard-guide-constraint 5 --penalty-strength 0.25 -gl 28 -pl 30 -pm 3 -pp 0.98 --primer-gc-content-bounds 0.35 0.65 --max-primers-at-site 10 --max-target-length 500 --obj-fn-weights 0.50 0.25’, with a cluster threshold such that there is only one cluster, and used our Cas13a activity model. When computing the fraction of sequences in the test set that are detected, we required the sequence to be detected by a primer on the 5′ and 3′ ends of a region (within three mismatches) and a probe (here, guide) to detect the region; we used the analyze_coverage.py program in ADAPT for this computation. We labeled detection of a sequence as ‘active’ if a guide in the guide set is decided by our Cas13a classification model to be active against the target. We labeled the detection as ‘highly active’ if a guide in the guide set is both decided to be active by the Cas13a classification model and its predicted activity, according to the Cas13a regression model, is ≥2.7198637 (4 added to the output of the model, −1.2801363). This threshold corresponds to the top 25% of predicted values on the subset of our hold-out test set that is classified as active.

Using the same cross-validation strategy, we also evaluated generalization except with relaxed settings on constraints for the number of guides and more stringent settings on primer coverage (Supplementary Fig. 19): ‘--obj maximize-activity --soft-guide-constraint 3 --hard-guide-constraint 10 --penalty-strength 0.05 -gl 28 -pl 30 -pm 3 -pp 0.995 --primer-gc-content-bounds 0.20 0.80 --max-primers-at-site 15 --max-target-length 1000 --obj-fn-weights 0.30 0.05’. These settings allow for more complex assay designs (for example, more guides and primers) to enable a higher sensitivity. Additionally, when deciding detection of the held-out genomes in this analysis, we adjusted thresholds to allow a higher sensitivity with lower precision: we allowed four mismatches for primers (instead of three) and lowered the decision threshold of our Cas13a classification model to 0.3 (instead of 0.577467).

#### Benchmarking trie-based specificity queries

We benchmarked the approach described in Supplementary Note 3d against a single, large trie (Supplementary Fig. 16). For this, we sampled 1.28% of all 28-mers from 570 viral species (~78.7 million 28-mers in total), and built data structures indexing these. We then randomly selected 100 species (here, counting each segment of a segmented genome as a separate species), and queried 100 randomly selected 28-mers from each of these for hits against the other 569 species. We performed this for varying choices of mismatches. We used the same approach to generate results in Supplementary Fig. 15, there comparing queries with and without tolerance of G-U base pairing.

#### Benchmarking runtime improvement with memoization

We benchmarked the effect on runtime of memoizing repeated computations (Supplementary Fig. 17), as described in Supplementary Note 4b. We used all genome neighbors from NCBI’s viral genomes resource^31^ as input for each of the three species tested. To run ADAPT while memoizing computations, we used the arguments: ‘--obj maximize-activity --soft-guide-constraint 1 --hard-guide-constraint 5 --penalty-strength 0.25 --maximization-algorithm random-greedy -pm 3 -pp 0.9 --primer-gc-content-bounds 0.3 0.7 --max-primers-at-site 10 -gl 28 --max-target-len 250 --best-n-targets 10 --id-m 4 --id-frac 0.01 --id-method shard’. We also used our Cas13a predictive model and enforced specificity against all other species within each species’ family. To perform runs without memoizing computations, we did the same except added the argument ‘--do-not-memoize-guide-computations’, which skips all memoization steps during ADAPT’s search (except for calls to the predictive model).

#### Design of broadly effective SARS-related CoV assays in 2018 and their evaluation

To evaluate the efficacy of species-level assays on a novel virus (Supplementary Fig. 21), we focused on SARS-related CoV. We simulated the 2018 design of assays for detecting the SARS-related CoV species, roughly a year before the initial detection of SARS-CoV-2. In particular, we used as input all genome neighbors from NCBI’s viral genomes resource^31^ for SARS-related CoV that were released on or before 31 December 2018 (there are 311 genomes). For ADAPT’s designs, we used the same parameters used for the vertebrate-infecting viral species designs (‘Designs across vertebrate-infecting species’), except tolerating up to one mismatch between primer and target sequences; the specificity criteria were also the same as in those designs.

In 2018, SARS-related CoV was biased toward SARS-CoV-1 genomes (owing to SARS outbreak sequencing) relative to viruses sampled from animals. To alleviate this overrepresentation, we also produced designs using ADAPT in which the input downsampled SARS-CoV-1 to a single genome (Supplementary Fig. 21c,d). We used the RefSeq, GenBank accession AY274119, as that genome.

To determine the performance of these designs on SARS-CoV-2, we used the 184,197 complete genomes (low-quality removed) available on GISAID^58^ as of 12 November 2020. For an assay to be predicted to detect a target sequence (Supplementary Fig. 21b,d), we require that (1) primers on both ends are within three mismatches of the target sequence; and (2) a guide in the guide set is classified by our Cas13a predictive model as active. We used these criteria for evaluating detection of SARS-CoV-2 and of the design’s input.

#### Designs across vertebrate-infecting species

We found all viral species in NCBI’s viral genomes resource^31^ that have a vertebrate as a host, as of April 2020. These are species ratified by the International Committee on Taxonomy of Viruses^76^. We added to this list others that may have been incorrectly labeled, as well as influenza viruses, which are separate from the resource. There were 1,933 species in total and we used ADAPT to design primers and Cas13a guides to detect them. As input, we used all genome neighbors from NCBI’s viral genomes resource^31^ (influenza database for influenza species^71^). We ran ADAPT in May–June 2020, and thus the input incorporates sequences available through those dates.

We constrained primers to have a length and GC content that are recommended for use with RPA^77^ (recombinase polymerase amplification), and thus are suitable for use with SHERLOCK^1^ (Specific High-Sensitivity Enzymatic Reporter UnLOCKing) detection. We enforced specificity at the species-level within each family. That is, we required that the guides for each species not have off-target hits to sequence from any other species in its same family. Restricting our specificity queries to one family at a time reduces ADAPT’s memory usage and runtime.

We used the following arguments when running ADAPT to maximize expected activity:

- Initial clustering: clustered with a maximum distance of 30% (‘--cluster-threshold 0.3’)
- Primers and amplicons: primer length of 30, primers must have GC content between 35% and 65%, at most 10 primers at a site (although high, this is only an upper bound and is meant to restrict the search space and thus restrict runtime), up to 3 mismatches between primers and target sequence for hybridization, primers must hybridize to ≥98% of sequences and length of a targeted genome region (amplicon) must be ≤250 nt (‘-pl 30 --primer-gc-content-bounds 0.35 0.65 --max-primers-at-site 10 -pm 3 -pp 0.98 --max-target-length 250’)
- Guides: Cas13a guide length of 28 nt, together with our Cas13a predictive model (‘-gl 28 --predict-activity-model-path models/classify/model-51373185 models/regress/model-f8b6fd5d’)
- Guide activity objective: soft constraint of 1 guide, hard constraint of 5 guides, guide penalty (*λ*) of 0.25, using the randomized greedy algorithm (‘--obj maximize-activity --soft-guide-constraint 1 --hard-guide-constraint 5 --penalty-strength 0.25 --maximization-algorithm random-greedy’)
- Specificity: query up to 4 mismatches counting G-U pairs as matches, calling a guide non-specific if it hits ≥1% of sequences in another taxon (‘--id-method shard --id-m 4 --id-frac 0.01’)
- Objective function and search: weights *λ*_*A*_ = 0.5 and *λ*_*L*_ = 0.25 in the objective function (defined in Supplementary Note 4b) and finding the best 20 design options (‘--obj-fn-weights 0.5 0.25 --best-n-targets 20’)

We made some species-specific adjustments. For influenza A and dengue viruses, two especially diverse species, we decreased the number of tolerated primer mismatches to two and allowed at most five primers at a site (‘-pm 2 --max-primers-at-site 5’); while these further constrain the design, they decrease runtime. For Norwalk virus and Rhinovirus C, we relaxed the number of primers at a site and the maximum region length to identify designs (‘--max-primers-at-site 20 --max-target-length 500’). For Cervid alphaherpesvirus 2, which has a short genome, we changed the GC-content bounds on primers to be 20–80% (‘--primer-gc-content-bounds 0.2 0.8’) to allow more potential amplicons. For 42 species, we relaxed specificity constraints to identify designs (list and details in code).

Of the 1,933 species, seven could not produce a design while maximizing activity and enforcing specificity, even with species-specific adjustments. They are: Bat mastadenovirus, Bovine associated cyclovirus 1, Chiropteran bocaparvovirus 4, Cyclovirus PKgoat21/PAK/2009, Finkel–Biskis–Jinkins murine sarcoma virus, Panine gammaherpesvirus 1 and Squirrel fibroma virus. Each of these seven species has just one genome sequence and ADAPT could not identify a guide set satisfying specificity constraints; it is possible they are misclassified or have very high genetic similarity to other species. When showing results for this objective, we report on 1,926 species.

In addition to using the above settings, which maximizes activity and enforces specificity, we ran ADAPT with three other approaches. We minimized the number of guides while enforcing specificity, requiring that guides be predicted to be highly active (as defined in ‘Evaluating dispersion and generalization’) in detecting 98% of sequences. We also ran the objectives to maximize activity and minimize guides without enforcing specificity. In total, 67 of the 1,933 species did not yield a design when minimizing the number of guides and enforcing specificity, owing to the constraints with this objective: ADAPT could not identify a guide set that is predicted to be highly active and achieves the desired coverage and specificity.

For species with segmented genomes, we ran ADAPT and produced designs separately for each segment. We then selected the segment whose highest-ranked design option has the best objective value (if multiple clusters, according to the largest cluster). We expect the selected segment to generally be the most conserved one.

In all analyses showing results of the designs (for example, number of guides, guide activity and target length), we used the highest-ranked design option output by ADAPT. For the species with more than one cluster, we report the mean across clusters from the highest-ranked design option in each cluster.

For producing designs across vertebrate-infecting viral species, we ran ADAPT on Amazon Web Services using the ‘x1.16xlarge’ instance type. We ran ADAPT in parallel across multiple species to fully use the instance’s resources. We evaluated ADAPT’s computational requirements, namely the runtime and memory usage, as part of these runs on that instance type.

### Designs for evaluating sensitivity and specificity

#### ADAPT design parameters

To generate designs with ADAPT for experimental testing, we used the following arguments unless otherwise noted:

- Initial clustering: force a single cluster (‘--cluster-threshold 1.0’)
- Primers and amplicons: primer length of 30, primers must have GC content between 35% and 65%, at most 5 primers at a site, up to 1 mismatch between primers and target sequence for hybridization, primers must hybridize to ≥98% of sequences and length of a targeted genome region (amplicon) must be ≤250 nt (‘-pl 30 --primer-gc-content-bounds 0.35 0.65 --max-primers-at-site 5 -pm 1 -pp 0.98 --max-target-length 250’)
- Guides: Cas13a guide length of 28 nt, together with our Cas13a predictive model (‘-gl 28 --predict-activity-model-path models/classify/model-51373185 models/regress/model-f8b6fd5d’)
- Guide activity objective: soft constraint of 1 guide, hard constraint of 5 guides, guide penalty (*λ*) of 0.25, using the randomized greedy algorithm (‘--obj maximize-activity --soft-guide-constraint 1 --hard-guide-constraint 5 --penalty-strength 0.25 --maximization-algorithm random-greedy’)
- Specificity: query up to 4 mismatches counting G-U pairs as matches, calling a guide non-specific if it hits ≥1% of sequences in another taxon (‘--id-method shard --id-m 4 --id-frac 0.01’)
- Objective function and search: weights *λ*_*A*_ = 0.5 and *λ*_*L*_ = 0.25 in the objective function (defined in Supplementary Note 4b) (‘--obj-fn-weights 0.5 0.25’)

For SARS-CoV-2 input sequences, we used the 9,054 complete genomes available on GISAID^58^ as of 28 April 2020. We also used genomes from GISAID for pangolin SARS-like CoV input sequences (isolates from Guangxi, China and Guandong, China). For all other input sequences—SARS-like CoV isolates RaTG13, ZC45 and ZXC21; other SARS-like CoVs; SARS-CoV-1 (also referred to as SARS-CoV); and other *Coronaviridae* species—we used all genome neighbors from NCBI from each taxon^31^. For input sequences to EVB designs, we also used genome neighbors from NCBI.

#### Generating test target sequences

Experimentally testing design options output by ADAPT also requires generating representative target sequences. We found representative sequences for a design option, using a collection of genomes spanning diversity of a taxon, as follows: (1) We extracted the amplicon (according to provided positions, for example, from primer sequences), while extending outward to achieve a minimum length (usually 500 nt). (2) We removed sequences that are too short, for example, owing to gaps in the alignment. (3) We computed pairwise Mash distances^78^ and performed hierarchical clustering (average linkage) to achieve a desired number of clusters or a maximum intercluster distance. (4) To avoid outliers, we greedily selected (in order of descending size) clusters that include a desired total fraction of sequences, or a particular number of targets, or ones representing particular taxa (specifics below). (5) We computed the medoid of each cluster—that is, the sequence with minimal total distance to all other sequences in the cluster. (6) We used the medoids of each of the clusters as representative target sequences. The pick_test_targets.py program in ADAPT implements the procedure and we used this program.

#### Baseline distribution of activity

We established a baseline distribution of activity using Cas13a guides, to detect SARS-CoV-2, selected from the genomic regions targeted by the US CDC’s RT–qPCR assays^79^. In particular, we picked ten random 28-mers from the US CDC’s N1 amplicon that have a non-G PFS and used these as Cas13a guides, according to the ‘hCoV-19/Wuhan/IVDC-HB-01/2019’ genome^58^. We also chose another Cas13a guide at the site of the TaqMan probe with a non-G PFS. We did the same from the US CDC N2 amplicon. In addition, in the N1 and N2 amplicons, we used ADAPT to design a single guide with maximal activity (ignoring specificity) from within the amplicon. This provides 24 guides in total.

#### Experimental designs with ADAPT

To evaluate the activity and lineage-level specificity of SARS-CoV-2 designs, we used ADAPT to produce ten design options for detecting SARS-CoV-2. We increased the specificity in ADAPT to call a guide non-specific if it hits any sequence outside SARS-CoV-2 and also used the greedy maximization to obtain more intuitive outputs because, in this case, we expect only a single Cas13a guide for each design option (‘--id-frac 0 --maximization-algorithm greedy’). We enforced specificity to not detect any sequences outside of SARS-CoV-2 from the SARS-related CoV species (including related bat and pangolin coronavirus isolates) and also to not detect sequences from the other 43 species in the *Coronaviridae* family. Owing to experimental constraints, we tested the highest-ranked five. We generated targets for each design option against which to test, using the ones representative of SARS-CoV-2; pangolin SARS-like CoVs (isolates from Guangxi, China); bat SARS-like CoV isolates ZC45 and RaTG13; and SARS-CoV-1.

To further evaluate activity and subspecies-comprehensiveness, we used ADAPT to produce ten design options for detecting the SARS-CoV-2-related taxon. In referring to SARS-CoV-2-related, we use the definition given in Fig. 1b of ref. ^48^; it encompasses SARS-CoV-2 and several related bat and pangolin SARS-like coronaviruses. To correct for sampling biases, we used ten sampled SARS-CoV-2 genomes as input so that they make up roughly half of sequences in the SARS-CoV-2-related taxon. We used the same adjusted arguments in ADAPT as used for the SARS-CoV-2 designs (‘--id-frac 0 --maximization-algorithm greedy’). We enforced specificity to not detect any sequences outside of SARS-CoV-2-related from the SARS-related CoV species (including other bat SARS-like coronaviruses) and also to not detect sequences from the other 43 species in the *Coronaviridae* family. For each design option, we generated targets, and used the ones representative of SARS-CoV-2; pangolin SARS-like CoVs (isolates from Guangxi, China and Guangdong, China); bat SARS-like CoV isolates ZC45, ZXC21 and RaTG13; and SARS-CoV-1. For this experiment, the SARS-CoV-1 target allows us to evaluate specificity, while the others allow us to evaluate activity and subspecies-comprehensiveness.

We used ADAPT to produce ten design options to detect the SARS-related CoV species, and we used these to evaluate activity, species-comprehensiveness and specificity. To correct for sampling biases, we used 300 sampled SARS-CoV-2 genomes as input so that they make up roughly half of sequences in the species. We enforced specificity to not detect sequences from the other 43 species in the *Coronaviridae* family. For each design option, we generated representative targets that encompass SARS-CoV-2, SARS-CoV-1, bat SARS-like CoVs, pangolin SARS-like CoVs, MERS-CoV, Human coronavirus OC43 and Human coronavirus HKU1. There were eight or nine representative targets in total for each design option.

To evaluate species-comprehensiveness, we focused on EVB and used ADAPT to produce ten design options. Owing to its extensive diversity, we made several adjustments to arguments, which help to increase the space of potential design options (‘--primer-gc-content-bounds 0.30 0.70 -pm 4 -pp 0.80 --max-primers-at-site 10 --id-frac 0.10 --penalty-strength 0.15’).

We enforced specificity to not detect the 18 other species in the *Enterovirus* genus. For each design option, we generated representative targets from clusters that encompass at least 90% of all sequences. There were between 1 and 15 targets for each design option (the precise number depends heavily on the location of the design option amplicon in the genome). We additionally tested specificity within the *Enterovirus* genus by generating a single representative target for each of Enterovirus A, Enterovirus C and Enterovirus D.

To benchmark ADAPT’s designs for EVB, we created baseline Cas13a guides using an entropy-based approach that identifies conserved sites. For each of ADAPT’s design options, we considered the amplicon it targets. Then, we computed the information-theoretic (Shannon) entropy, over alleles, at every site in the amplicon. (We counted an ambiguous base fractionally and a gap as a ‘base’.) We define the average entropy of a 28-nt site to be the mean entropy across its 28 positions. The approach finds the site in the amplicon that has the minimal average entropy and an active (non-G) PFS in GenBank accession MK800120. Our entropy-based baseline guide is the sequence from GenBank accession MK800120 at this site. We performed this process in the amplicon from each of ADAPT’s designs to generate and test one baseline guide; for five of the ten designs, we generated and tested two baseline guides, where the second was from the site with the second lowest entropy and an active PFS. The approach is implemented in ADAPT’s design_naively.py program.

We built a positive control into each target. In particular, we added the sequence 5′-CACTATAGGGGCTCTAGCGACTTCTTTAAATAGTGGCTTAAAATAAC-3′ to the 5′ end of each target and included in our tests of every target a guide with protospacer sequence 5′-GCTCTAGCGACTTCTTTAAATAGTGGCT-3′.

### Experiments evaluating sensitivity and specificity

#### Experimental procedure

We largely followed the CARMEN-Cas13 platform^8^ for experimentally validating ADAPT’s designs, with some key differences. DNA targets were ordered from Integrated DNA Technologies and in vitro transcribed using the HiScribe T7 High Yield RNA Synthesis Kit (New England Biolabs). Transcriptions were performed according to the manufacturer’s recommendations with a reaction volume of 20 µl that was incubated overnight at 37 °C. The transcribed RNA products were purified using RNAClean XP beads (Beckman Coulter) and quantified using NanoDrop One (Thermo Scientific). The RNA was serially diluted from 10^11^ to 10^4^ copies per µl and used as input into the detection reaction. crRNAs were synthesized by Integrated DNA Technologies, resuspended in nuclease-free water and diluted to 1 µM for input into the detection reaction. The Cas13 detection reactions were made into two separate mixes for loading onto a 192.24 Dynamic Array integrated fluidic circuit (IFC) for Gene Expression (Fluidigm). The assay mix contained 42.5 nM LwaCas13a, 42.5 nM crRNA, 2× Assay Loading Reagent (Fluidigm) and nuclease-free water. The sample mix contained 1 µl of RNAse Inhibitor (New England Biolabs), 1× ROX Reference Dye (Invitrogen), 1× GE Sample Loading Reagent (Fluidigm), 1.95 nM quenched synthetic fluorescent RNA reporter (FAM/rUrUrUrUrUrUrU/3IABkFQ/, Integrated DNA Technologies) and 9 nM MgCl_2_ in a nuclease assay buffer (40 mM Tris-HCl, 1 mM dithiothreitol pH 7.5). Syringe, Actuation Fluid, Pressure Fluid (Fluidigm) and 4 µl of assay and sample mixtures were loaded into their respective locations on a 192.24 IFC according to the manufacturer’s instructions. The IFC was loaded onto the IFC Controller RX (Fluidigm) where the ‘Load Mix’ script was run. After proper IFC loading, images over a 2-h period were collected using a custom protocol on Fluidigm’s Biomark HD.

#### Displaying experimental results

We plotted reference-normalized background-subtracted fluorescence for guide–target pairs. For a guide–target pair (at some time point *t* and target concentration), we first computed the reference-normalized value as

$${{{\mathrm{median}}}}\left( {\frac{{P_t - P_0}}{{R_t - R_0}}} \right)$$

where *P*_*t*_ is the guide signal (FAM) at the time point, *P*_0_ is its background measurement before the reaction, *R*_*t*_ is the reference signal (ROX) at the time point, *R*_0_ is its background measurement and the median is taken across Fluidigm’s replicates. We performed the same calculation for the no-template (water) control of the guide, providing a background fluorescence value for the guide at *t* (when there were multiple technical replicates of such controls, we took the mean value across them). The reference-normalized background-subtracted fluorescence for a guide–target pair is the difference between these two values. Note that, by definition, plotted values greater than 0 represent fluorescence that exceeds background and the no-template control (‘NC’ in figures) has value of 0. When plotting the no-template control separately (Supplementary Fig. 23), we show reference-normalized values without background-subtracting. In heatmaps showing fluorescence at a fixed time point, we used the middle time point (59 min). In kinetic curves that show fluorescence over time (for example, Fig. 5b), we smoothed the value by taking the rolling mean within a window of two time points.

When displaying the top-ranked design options from ADAPT (for example, in Fig. 5d–h), we ordered them according to the predicted activity of the Cas13a guides in expectation across the input genomes. ADAPT’s ranking incorporates additional factors (Supplementary Note 4b) that reflect amplification potential, and we used ADAPT’s objective function to identify the top *N* design options to test. But we ordered them according to only predicted fluorescent activity because our experimental testing did not involve amplification. When plotting fluorescence for a design that uses more than one guide, we plot the maximum fluorescence across the guides (computed separately at each target, target concentration and measurement time point). This is analogous to ADAPT’s model for measuring a probe set’s activity (Supplementary Note 2a), in which its activity in detecting a target sequence equals that of the best probe in the set for detecting that sequence.

#### Evaluating specificity against non-viral taxa

We performed an in silico comparison of all experimentally tested guides with human transcripts and bacterial pathogens to determine if there is potential cross-reactivity. We first built an index consisting of human transcript sequences from GENCODE v.38 (ref. ^80^) and NCBI reference genome sequences for 11 bacterial pathogens (*Bordetella pertussis* (NC_018518.1); *Chlamydia pneumoniae* (NC_005043.1); *Haemophilus influenzae* (NZ_CP009610.1); *Legionella pneumophila* (NZ_CP013742.1); *Mycobacterium tuberculosis* (NC_000962.3); *Mycoplasma pneumoniae* (NZ_CP010546.1); *Pseudomonas aeruginosa* (NC_002516.2); *Staphylococcus epidermidis* (NZ_CP035288.1); *Streptococcus pneumoniae* (NZ_CP046357.1); *Streptococcus pyogenes* (NZ_CP010450.1); *Streptococcus salivarius* (NZ_CP066093.1)). We also included, as positive controls for the analysis, NCBI reference sequence genomes for SARS-CoV-1 (NC_004718.3) and SARS-CoV-2 (NC_045512.2).

We sought to query guide sequences against this index while tolerating multiple mismatches over a short query length (that is, the guide length of 28 nt). To enable this, we used Bowtie 2 (ref. ^81^) to align guide sequences to the index with the parameters ‘-a --end-to-end -N 1 -L 7 -i S,1,1 --ma 0 --mp 1,1 --rdg 100,1 --rfg 100,1 --score-min L,-4,0’. These settings permit us to identify all alignments of guides, against our index, having four or fewer mismatches across the length of the guide without tolerating gaps. Such alignments represent potential non-specificity of the guides. Of all guides in our experimental testing, the only identified non-specificity was for one guide from the entropy-based strategy for benchmarking EVB detection (Design no. 8; four mismatches from a human transcript); thus, with this exception, all guides are at least five mismatches different from human transcripts and the included bacterial genomes.

### Reporting Summary

Further information on research design is available in the Nature Research Reporting Summary linked to this article.

## Online content

Any methods, additional references, Nature Research reporting summaries, source data, extended data, supplementary information, acknowledgements, peer review information; details of author contributions and competing interests; and statements of data and code availability are available at 10.1038/s41587-022-01213-5.

## Supplementary information

Supplementary Information Supplementary Notes 1–5 and Figs. 1–27.

Reporting Summary

## Data Availability

Data are available in several repositories: the CRISPR–Cas13a library and activity dataset is available at https://github.com/broadinstitute/adapt-seq-design/tree/main/data; serialized trained models are available at https://github.com/broadinstitute/adapt-seq-design/tree/main/models/cas13; experimentally tested designs and their measured data are available at https://github.com/broadinstitute/adapt-designs/tree/main/experimentally-tested.

## Code availability

Code is available in several repositories: ADAPT is freely available under the MIT license at https://github.com/broadinstitute/adapt; code to replicate the predictive modeling and analyses is available at https://github.com/broadinstitute/adapt-seq-design; code to replicate the designs across the vertebrate-infecting viral species is available at https://github.com/broadinstitute/adapt-designs-continuous; code to replicate the other analyses in this paper is available at https://github.com/broadinstitute/adapt-analysis.
