# Supplementary material for: Designing sensitive viral diagnostics with machine learning
Source: Nat Biotechnol. Author manuscript; Available in PMC 2022 Jul 21. (PMC9287178; doi:10.1038/s41587-022-01213-5)
Supplement: Supplementary Information [file NIHMS1820058-supplement-Supplementary_Information.pdf]

nature portfolio

https://doi.org/10.1038/s41587-022-01213-5

Supplementary information

Designing sensitive viral diagnostics with machine learning

In the format provided by the authors and unedited

Supplementary Note 1

This note describes a comparison of our model's LwaCas13a predictions with data from two independent studies. Results are in Extended Data Fig. 5.

1a Results

We tested our model's performance on two independent datasets, starting with ref. 1. Measurements from this study used a different ortholog (LbuCas13a) than the one used for training our model (LwaCas13a): we mitigated the effects of LbuCas13a's higher overall cleavage activity[2] and differing guide lengths (see Methods below). Following these changes (Extended Data Fig. 5a), our model's predictions of LwaCas13a activity correlate with the measured LbuCas13a cleavage rates (Spearman's $\rho = 0.816$ with $p < 10^{-4}$; Extended Data Fig. 5b), providing validation of our model. Considering data on LbuCas13a—RNA binding affinity from this same study, pairs with high predicted collateral activity rarely exhibit low binding affinity, whereas pairs with high binding affinity can have low predicted collateral activity (Extended Data Fig. 5c–f). This relationship is consistent with binding being necessary, but not sufficient, to achieve Cas13a collateral activity.

We also tested our model on data from ref. 3, which assessed on-target (*cis*-) cleavage by measuring RNA knockdown using LwaCas13a. Our model's LwaCas13a collateral activity predictions correlate highly with LwaCas13a knockdown levels (Spearman's $\rho = -0.826$ with $p < 10^{-10}$; Extended Data Fig. 5g). The roughly linear relationship between on-target and predicted collateral cleavage activity (Pearson's $r = -0.868$ with $p < 10^{-12}$) suggests that our model could predict Cas13a on-target cleavage and that high-throughput on-target assays could be valuable for modeling Cas13a collateral activity. These results provide another independent validation of our model's performance and show its generalizability.

1b Methods

We compared our model's predictions with data from ref. 1. Although our model was trained for LwaCas13a whereas the data in ref. 1 is from LbuCas13a, the comparison enables validation on an independent dataset and the opportunity to assess whether RNA binding activity correlates with collateral (or *trans*-) cleavage activity. We padded the 20-nt spacer sequences used in the data with 8 randomly determined bases because our model requires 28-nt spacer sequences; in particular, we added `5'-AAAATCTG-3'` to the 3′ end of the crRNA spacer sequences, with matching complementary bases in the target sequences. Additionally, we padded the target sequences with random bases to obtain 10-nt on each side of the protospacer, as required by our model. In the RNA binding data: for crRNA X ('X' and 'Y' are as in ref. 1), we added `5'-AAGCATG-3'` to the 5′ end of the corresponding target sequence and `5'-TGCCATA-3'` to the 3′ end; for crRNA Y, we added `5'-GATTCAA-3'` to the 5′ end of the corresponding target sequence and `5'-CAGCATA-3'` to the 3′ end. In the collateral cleavage activity data: we added `5'-CATAT-3'` to the 3′ end of crRNA X's corresponding target sequence (the 5′ end has a sufficient number of context bases for our model, and crRNA Y was not a part of this experiment).

Along with padding the data from ref. 1 for input to our model, we made several other choices when handling the data from ref. 1 to allow for comparisons. For LbuCas13a–RNA binding measurements, we normalized the data as done in ref. 1. That is, we used fold-changes (ratio of read counts relative to apo-Cas13a, i.e., protein with no guide), and then normalized them by subtracting average fold-change for the off-target (for crRNA X, crRNA Y's target; for crRNA Y, crRNA X's target) and

then dividing by the average fold-change for the on-target (for crRNA X, crRNA X's target, and likewise for crRNA Y). In our comparison, we skipped data points where the fold-change could not be computed (reported as 'nan'; possibly, the control (apo-Cas13a) had 0 reads). To correct for substantial differences in the numbers of mismatches across data points, we randomly downsampled to 150 data points for each number of mismatches (the number of mismatches is a strong predictor of activity, and not downsampling would cause data points with fewer than 3 mismatches to be a small component of the comparison); we chose 150 because there are 156 data points with 1 mismatch, and more than 150 for each choice of ≥ 2 mismatches. For collateral cleavage measurements, we used 0 as the value from from ref. 1 (the normalized cleavage rate relative to no mismatches) for two data points labeled 'ND'.

When comparing measured LbuCas13a cleavage rates from ref. 1 to our model's predictions of LwaCas13a collateral activity, we also subsetted the data. On the full cleavage rate data (1 guide, 26 targets), our model's predictions show a weak and non-significant correlation with the measurements (Spearman's $\rho = 0.360$ with $p = 0.07$; Extended Data Fig. 5a). LbuCas13a's greater overall cleavage activity[2], compared to LwaCas13a, could explain the discrepancy. Thus, we also considered only the guide-target pairs where mismatches harm activity, removing the 8 mismatched targets where LbuCas13a exhibits even greater activity than against a matching target (Extended Data Fig. 5b).

We also compared our model's predictions with measurements (mean across replicates) of on-target (*cis-*) cleavage, namely LwaCas13a knockdown, from Figure 3a–c in ref. 3. For the target sequence of the Gluc guide 1 (defined in ref. 3), we used sequence from GenBank accession MF882921; for the *CXCR4* guide, we used sequence from NM_001008540; and for the Gluc guide 2, we used sequence from MF882921. To insert mismatches into spacers, we randomly selected an allele for the mismatch at the positions given in the data.

For all comparisons, when making predictions on these data with our model, we used the hurdle model (piecewise function) described in Methods with the same high-precision decision threshold on the classifier that we use in ADAPT.

Supplementary Note 2

This note describes two formulations for objective functions implemented in ADAPT. Unless otherwise noted, for designs and analyses in the paper, we use the formulation in Design formulation #1: maximizing expected activity.

2a Design formulation #1: maximizing expected activity

Objective

Let S be an alignment of sequences from species t in a genomic region. We wish to find a set P of probes that maximizes detection activity over these sequences. This objective bears some similarity to the problem of designing PCR primers that cover a maximum number of sequences[4], though solutions to that problem binarize decisions about detection rather than accommodate continuous predictions. As described in Methods, we have a model to predict a measurement of detection activity between one probe p and one sequence $s \in S$, which we represent by $d(p, s)$. (In Fig. 3a, we use $A(P, s)$ to represent this quantity.) While more than one probe in the set P may be able to detect s, we use the best probe against s to measure P's detection activity for s; that is, the predicted detection activity is

$$d(P, s) = \max_{p \in P} d(p, s).$$

One way to consider why taking the maximum is reasonable is that, in practice, we could apply each $p \in P$ in parallel reactions to a sample and obtain a readout even if only one p works well for the particular target in that sample. We also define $d(P, s)$ when P is the empty set to be the lowest value in the range of $d(p, s)$, indicating no detection (in practice, 0).

We represent the predicted detection activity by P, against all sequences in S, with the function $F(P)$. $F(P)$ is the expected value of $d(P, s)$ taken over all the $s \in S$; the weights w_s for each s can reflect a prior probability on applying a diagnostic when the target sequence is like s (e.g., a prior of observing s based on the genome's date or geographic location). Thus, we define

$$F(P) = \sum_{s \in S} w_s \cdot d(P, s).$$

In practice, we usually set a uniform prior over targeting the genomes in S, with the effect being that all w_s are equal.

We must introduce penalties for the number of probes. Striving for a small number of probes is important because (a) if probes are used in separate reactions, they add time and labor; (b) if multiplexed in one reaction, having more may reduce the resulting detection signal; and (c) they require time and money to synthesize, and to experimentally validate. For this penalty, we use a soft constraint m_p and hard constraint $\overline{m_p}$ on the number of probes, with $\overline{m_p} \geq m_p$. We wish to solve

$$\max_{P} \{F(P) - \lambda \cdot \max(0, |P| - m_p) : |P| \leq \overline{m_p}\}$$

where $\lambda > 0$ serves as a weight on the penalty. λ reflects a tolerance for higher $F(P)$ at the expense of more probes.

Submodularity of the objective

Let $\widetilde{F}(P) = F(P) - \lambda \cdot \max(0, |P| - m_p)$. We want to prove that $\widetilde{F}(P)$ is submodular.

We start by showing first that $d(P, s)$ is submodular. For ease of notation, we drop s, referring to $d(P, s)$ as $d(P)$ and $d(p, s)$ as $d(p)$. Consider probe sets A and B, with $A \subseteq B$, and some possible probe $x \notin B$. Note that

$$\begin{aligned} d(A \cup \{x\}) &= \max_{p \in A \cup \{x\}} d(p) \\ &= \max(\max_{p \in A} d(p), d(x)) \\ &= \max(d(A), d(x)) \end{aligned}$$

and therefore $d(A \cup \{x\}) - d(A) \geq 0$. Likewise, $d(B \cup \{x\}) = \max(d(B), d(x))$, and $d(B \cup \{x\}) - d(B) \geq 0$. Also, since $A \subseteq B$, we have

$$\begin{aligned} d(B) &= \max_{p \in B} d(p) \\ &= \max(\max_{p \in A} d(p), \max_{p \in B \setminus A} d(p)) \\ &= \max(d(A), \max_{p \in B \setminus A} d(p)) \\ &\geq d(A). \end{aligned}$$

We now consider two cases:

- Assume $d(B) \geq d(x)$. Then, $d(B \cup \{x\}) - d(B) = \max(d(B), d(x)) - d(B) = 0$. Therefore, $d(A \cup \{x\}) - d(A) \geq d(B \cup \{x\}) - d(B)$.
- Assume $d(B) < d(x)$. It follows from $d(B) \geq d(A)$ that $d(x) > d(A)$ and that $d(x) - d(A) \geq d(x) - d(B)$. Since $\max(d(A), d(x)) = d(x)$ and $\max(d(B), d(x)) = d(x)$, we have $\max(d(A), d(x)) - d(A) \geq \max(d(B), d(x)) - d(B)$. Therefore, in this case as well, $d(A \cup \{x\}) - d(A) \geq d(B \cup \{x\}) - d(B)$.

Hence, $d(P, s)$ is submodular. Since $F(P)$ is a non-negative linear combination of $d(P, s)$, it follows that $F(P)$ is submodular.

Using the above result, we show that $\widetilde{F}(P)$ is submodular. Again, consider probe sets A and B, with $A \subseteq B$, and some possible probe $x \notin B$. We want to show that $\widetilde{F}(A \cup \{x\}) - \widetilde{F}(A) \geq \widetilde{F}(B \cup \{x\}) - \widetilde{F}(B)$. We have that

$$\begin{aligned} \widetilde{F}(A \cup \{x\}) - \widetilde{F}(A) &= F(A \cup \{x\}) - \lambda \cdot \max(0, |A| + 1 - m_p) - F(A) + \lambda \cdot \max(0, |A| - m_p) \\ &= F(A \cup \{x\}) - F(A) - \lambda(\max(0, |A| + 1 - m_p) - \max(0, |A| - m_p)) \\ &\geq F(B \cup \{x\}) - F(B) - \lambda(\max(0, |A| + 1 - m_p) - \max(0, |A| - m_p)) \qquad (\star) \end{aligned}$$

where the last step follows from submodularity of $F(P)$. We now consider two cases:

- Assume $|A| \geq m_p$. Since $A \subseteq B$, $|B| \geq m_p$. Continuing from ($\star$), we have

$$\begin{aligned} \widetilde{F}(A \cup \{x\}) - \widetilde{F}(A) &\geq F(B \cup \{x\}) - F(B) - \lambda(|A| + 1 - m_p - (|A| - m_p)) \\ &= F(B \cup \{x\}) - F(B) - \lambda \\ &= F(B \cup \{x\}) - F(B) - \lambda(|B| + 1 - m_p - (|B| - m_p)) \\ &= F(B \cup \{x\}) - \lambda(|B| + 1 - m_p) - [F(B) - \lambda(|B| - m_p)] \\ &= F(B \cup \{x\}) - \lambda \cdot \max(0, |B \cup \{x\}| - m_p) - [F(B) - \lambda \cdot \max(0, |B| - m_p)] \\ &= \widetilde{F}(B \cup \{x\}) - \widetilde{F}(B). \end{aligned}$$

- Assume $|A| < m_p$. Continuing from ($\star$) in this case, we now have

$$\begin{aligned}\widetilde{F}(A \cup \{x\}) - \widetilde{F}(A) &\geq F(B \cup \{x\}) - F(B) \\ &\geq F(B \cup \{x\}) - F(B) - \lambda[\max(0, |B| + 1 - m_p) - \max(0, |B| - m_p)] \\ &= F(B \cup \{x\}) - \lambda \cdot \max(0, |B| + 1 - m_p) - [F(B) - \lambda \cdot \max(0, |B| - m_p)] \\ &= F(B \cup \{x\}) - \lambda \cdot \max(0, |B \cup \{x\}| - m_p) - [F(B) - \lambda \cdot \max(0, |B| - m_p)] \\ &= \widetilde{F}(B \cup \{x\}) - \widetilde{F}(B).\end{aligned}$$

Hence, $\widetilde{F}(P)$ is submodular.

Note that $\widetilde{F}(P)$ is non-monotone owing to the penalty term.

That the function is non-monotone means that while initially its value will increase as the number of probes increases, the value may eventually decrease; this is an expected property because, while adding more probes may initially improve detection, our function penalizes the number of them. Submodularity corresponds to the property of "diminishing returns"—that is, as the number of probes increases, the improvement in performance from each additional probe diminishes, which is also an expected property.

Non-negativity of the objective

Now we show how to ensure that $\widetilde{F}(P)$ is non-negative. Let P only contain probes p such that $F(\{p\}) \geq \lambda \cdot (\overline{m_p} - m_p)$. Since F is monotonically increasing, $F(P) \geq \lambda \cdot (\overline{m_p} - m_p)$. Thus,

$$\begin{aligned}\widetilde{F}(P) &= F(P) - \lambda \cdot \max(0, |P| - m_p) \\ &\geq \lambda \cdot (\overline{m_p} - m_p) - \lambda \cdot \max(0, |P| - m_p).\end{aligned}$$

If $|P| \leq m_p$, then

$$\begin{aligned}\widetilde{F}(P) &\geq \lambda \cdot (\overline{m_p} - m_p) - 0 \\ &\geq 0\end{aligned}$$

where the last inequality follows from $\overline{m_p} \geq m_p$. If $|P| > m_p$, then

$$\begin{aligned}\widetilde{F}(P) &\geq \lambda \cdot (\overline{m_p} - m_p) - \lambda \cdot (|P| - m_p) \\ &= \lambda \cdot (\overline{m_p} - m_p - |P| + m_p) \\ &= \lambda \cdot (\overline{m_p} - |P|) \\ &\geq 0\end{aligned}$$

where the last inequality follows from $\overline{m_p} \geq |P|$. If P is the empty set, $\widetilde{F}(P) = 0$ according to our definition of $d(P, s)$. Therefore, $\widetilde{F}(P) \geq 0$ always. Let our ground set Q be the set of probes from which we select P—i.e., $P \subseteq Q$. To enforce non-negativity, we restrict Q to only contain probes p such that $F(\{p\}) \geq \lambda \cdot (\overline{m_p} - m_p)$. In other words, every probe has to be sufficiently good. In practice, given our activity function, $\lambda \in [0.1, 0.5]$ is a reasonable choice and the constraint on $F(\{p\})$ is generally met; for example, with $\lambda = 0.25$, $\overline{m_p} = 5$, and $m_p = 1$, then we require $F(\{p\}) \geq 1$.

Algorithm 1 Construct set of probes P to maximize $\widetilde{F}(P)$ subject to hard constraint.

Input

T	alignment of sequences extracted from a window of S, from taxon t
l_p	probe length
F	function of probe set, including soft constraint
$\overline{m_p}$	hard constraint on number of probes

Output

P	collection of probes

1 **function** DETERMINE-PROBE-SET(T, l_p, $\widetilde{F}$, $\overline{m_p}$)
2 $\quad C \leftarrow$ Clusters of l_p-mers at and across each position of T
3 $\quad Q \leftarrow$ Representative (consensus) of each cluster in C ▷ ground set
4 $\quad Q \leftarrow Q \setminus \{l_p$-mers in Q not meeting non-negativity constraint$\}$
5 $\quad Q \leftarrow Q \setminus \{l_p$-mers in Q not specific to $t\}$ ▷ enforce specificity
6 $\quad Q \leftarrow Q \cup \{2 \cdot \overline{m_p}$ "dummy" elements that contribute 0 to $\widetilde{F}\}$
7
8 $\quad P \leftarrow \{\}$
9 $\quad$ **for** $j \leftarrow 1$ to $\overline{m_p}$ **do**
10 $\qquad M_j \leftarrow \overline{m_p}$ elements from $Q \setminus P$ that maximize $\sum_{u \in M_j} \left(\widetilde{F}(P \cup \{u\}) - \widetilde{F}(P)\right)$
11 $\qquad p^* \leftarrow$ Element from M_j chosen uniformly at random
12 $\qquad P \leftarrow P \cup \{p^*\}$
13 $\quad P \leftarrow P \setminus \{$"dummy" elements$\}$
14 $\quad$ **return** P

Solving for P

Recall we want to solve

$$\max_P \left\{ \widetilde{F}(P) : |P| \leq \overline{m_p} \right\}$$

where $\widetilde{F}(P) = F(P) - \lambda \cdot \max(0, |P| - m_p)$. We need to maximize a non-negative and non-monotone submodular function subject to a cardinality constraint. We apply the recently-developed discrete randomized greedy algorithms in ref. 5, namely Algorithm 1. It provides a $1/e$-approximation for non-monotone functions—i.e., we obtain a probe set with an objective value within a factor $1/e$ of the optimal. (Algorithm 5, which provides a better approximation ratio, is likely to not be much better in our case because the constraint $\overline{m_p}$ is small compared to the size of the ground set.)

Based on the work in ref. 5, the function DETERMINE-PROBE-SET (Algorithm 1) shows how we compute P to detect a particular genomic window of an alignment S. We use locality-sensitive hashing to rapidly cluster potential probe sequences throughout the window, and their representatives form the ground set Q of probes (line 3). In particular, we sample nucleotides—i.e., concatenate locality-sensitive hash functions drawn from a Hamming distance family—and take the consensus of sequences within each cluster, where clusters are defined by their hash values. Then, we require that probes in Q be specific to the taxon to which S belongs, using the methods in Supplementary Note 3d). We add to the ground set "dummy" elements that provide a marginal contribution of 0 to any set input to $\widetilde{F}$ (line 6), as required by an assumption of the algorithm (Reduction 1 in ref. 5). Then, we greedily choose $\leq \overline{m_p}$ probes, at each iteration selecting one randomly from a set of not-yet-chosen probes that maximize marginal contributions to $\widetilde{F}$ (lines 10–11).

The runtime to design probes is practical in the typical case. Here we ignore the runtime of

evaluating specificity (line 5), which is given in Supplementary Note 3d. Let L be the window length and n be the number of sequences. There are $O(nL)$ probes in the ground set in the worst-case, and they take $O(nL)$ time to construct (line 3). Finding the $\overline{m_p}$ elements that maximize marginal contributions (line 10) takes $O(nL)$ time, and we do this $O(\overline{m_p})$ times. Thus, the runtime in the worst-case is $O(nL\overline{m_p})$. In a typical case, the number of clusters at a position in the window is a small constant ($\ll n$) owing to sequence homology in the alignment; thus, the size of the ground set is $O(L)$, although it still takes $O(nL)$ time to construct. Now, finding the $\overline{m_p}$ elements that maximize marginal contributions takes $O(L)$ time, and we do this $O(\overline{m_p})$ times. So the runtime in a typical case is $O(nL + L\overline{m_p})$. Note that, in general, $\overline{m_p} \ll L$ and $\overline{m_p} \ll n$.

We also evaluated the classical discrete greedy algorithm[6] for submodular maximization. It offers similar results in practice (Supplementary Fig. 13), but does not offer theoretical guarantees in our case because it assumes a monotone function.

2b Design formulation #2: minimizing the number of probes

Objective

As in the above objective, let S be an alignment of sequences from species t in a genomic region and let $d(p, s)$ be a predicted detection activity between one probe p and one sequence $s \in S$. We wish to find a set P of probes with minimal $|P|$ that satisfies constraints on detection activity across these sequences. In particular, we introduce a fixed detection activity m_d and say that p is highly active in detecting s if $d(p, s) \geq m_d$. To define whether P detects a sequence with high activity, let

$$d(P, s) = \begin{cases} 1 & \text{if } |\{p : p \in P,\ d(p, s) \geq m_d\}| \geq 1 \\ 0 & \text{otherwise} \end{cases}$$

We additionally introduce a lower bound f_S on the minimal fraction of sequences in S that must be detected with high activity. Then, we wish solve

$$\min_P \{|P| : |\{s : s \in S,\ d(P, s) = 1\}|/|S| \geq f_S\}.$$

That is, we want to find the smallest probe set that detects, with high predicted activity, at least a fraction f_S of all sequences.

Solving for P

To approximate the optimal P, ADAPT follows the canonical greedy solution to the set cover problem[7,8] in which the universe consists of the sequences in S and each possible probe covers a subset of sequences in S. Similar approaches have been used for PCR primer selection[9–13]; in contrast to prior approaches, rather than starting with a collection of candidate probes (i.e., the sets), we construct them on-the-fly.

Iteratively, we approximate a probe that covers the most number of sequences that still need to be covered. Here, a probe p *covers* a sequence s if $d(p, s) \geq m_d$. Find-Optimal-Probe, shown in Algorithm 2, implements a heuristic. Briefly, at each position Find-Optimal-Probe rapidly clusters l_p-mers in the input sequences (l_p is the probe length) by sampling nucleotides—i.e., concatenating locality-sensitive hash functions drawn from a Hamming distance family—and uses each of these clusters to propose a probe. It iterates through the clusters in decreasing order of score, stopping early (line 11) if it is unlikely that remaining clusters will provide a probe that achieves more coverage than the current best. This procedure relies on two subroutines, Score-Cluster and Num-Detect, that are described below.

Algorithm 2 Construct probe p^* with highest coverage.

Input

M sequences in S to cover, from taxon t
l_p probe length

Output

p^* probe in window

function FIND-OPTIMAL-PROBE(M, l_p)
  Initialize p^*
  for each length l_p sub-window w in M **do**
    $clusts \leftarrow$ Cluster all l_p-mers of M in w
    $clusts \leftarrow$ Sort $clusts$, descending, according to SCORE-CLUSTER
    repeat
      $p \leftarrow$ Representative (consensus) of l_p-mers in next best cluster in $clusts$
      if p is specific to taxon t **then** ▷ enforce specificity
        if NUM-DETECT$(p, M) >$ NUM-DETECT(p^*, M) **then**
          $p^* \leftarrow p$
    until early stopping criterion is met
  return p^*

Algorithm 3 Construct minimal collection of probes in window that collectively achieve desired detection coverage.

Input

T alignment of sequences extracted from a window of S, from taxon t
l_p probe length
f_S fraction of sequences in T to detect

Output

C collection of probes

function DETERMINE-PROBE-SET(T, l_p, f_S)
  $C \leftarrow \{\}$
  while $|\{s : s \in T,\ d(C, s) = 1\}|/|T| < f_S$ **do**
    $M \leftarrow$ Sequences $s \in T$ such that $d(C, s) = 0$
    $p^* \leftarrow$ FIND-OPTIMAL-PROBE(M, l_p)
    $C \leftarrow C \cup \{p^*\}$
  return C

Using this procedure, it is straightforward to construct a set of probes in the window (region) that achieve the desired coverage by repeatedly calling FIND-OPTIMAL-PROBE. This is shown by DETERMINE-PROBE-SET, in Algorithm 3. In other words, the output probes collectively detect, with high activity, the sequences in the region.

This approach, with on-the-fly construction of probes, is similar to a reduction to an instance of the set cover problem, the solution to which is essentially the best achievable approximation[14,15]. In such a reduction, each set would represent one of the 4^{l_p} possible probes, consisting of the sequences that it would detect with high activity. Then, each iteration would identify the probe that detects, with high activity, the most not-yet-covered sequences. Here, rather than starting with such a large

space, we use a heuristic to approximate the probe at each iteration.

The runtime to design probes in a window is poor in the worst-case but practical in the typical case. Let n be the number of sequences in the alignment and L be length of the window. In the worst-case, we choose n different probes in the window. Each choice requires iterating over $O(L)$ positions, and at each one we iterate through $O(n)$ clusters, taking $O(n)$ time to evaluate the probe proposed by each cluster with NUM-DETECT. Thus, this is $O(n^3 L)$ time. In a typical case, there is a small constant number of clusters owing to sequence homology across the alignment, and the number of probes needed to achieve the constraint is also a small constant. Selecting each probe requires iterating over $O(L)$ positions, and at each one we consider $O(1)$ clusters, taking $O(n)$ time again to evaluate the probe proposed. So the runtime is $O(nL)$ with these assumptions.

Scoring clusters and detection across sequences

Sequences from S can be *grouped* according to metadata such that each group receives a particular desired coverage (f_{S_g}). For example, in ADAPT's implementation they can be grouped according to year (each group contains sequences from one year), with a desired coverage that decays for each year going back in time, so that ADAPT weighs more recent sequences more heavily in the design.

There are two subroutines in Algorithm 2 that we consider here: scoring a cluster and computing the number of sequences detected by a probe. These must account for groupings. First, on line 5 of FIND-OPTIMAL-PROBE, the function SCORE-CLUSTER($clust$) computes the number of sequences $clust \in clusts$ contains that are needed to achieve the desired coverage across all the groups. That is, it calculates

$$\sum_{x \in X} \min(n_x, |\widetilde{clust} \cap M_x|)$$

where X is the collection of sequence groups, n_x is the number of sequences from group x that must still be covered to achieve x's desired coverage, $\widetilde{clust}$ gives the sequences of M from which the l_p-mers in $clust$ originated, and M_x consists of the sequences in M that are in group x. In essence, it computes a contribution of each cluster toward achieving the needed coverage of each group, summed over the groups. Similarly, on line 9 of FIND-OPTIMAL-PROBE, the function NUM-DETECT(p, M) is the detection coverage provided by probe p across the groups. In particular, its value is

$$\sum_{x \in X} \min(n_x, |B \cap M_x|)$$

where B is the set of sequences in M that p covers—i.e., $B = \{s : s \in M,\ d(p, s) \geq m_d\}$.

These subroutines are intuitive in the case where sequences are not grouped. Equivalently, consider a single group x_0. Here, SCORE-CLUSTER($clust$) is $\min(n_{x_0}, |\widetilde{clust} \cap M_{x_0}|)$. Since $\widetilde{clust} \subseteq M_{x_0} = M$, this is $\min(n_{x_0}, |\widetilde{clust}|)$. Thus, the score is simply the size of the cluster (larger clusters are preferred), or n_{x_0} for clusters large enough so as to provide more than sufficient coverage. Similarly, NUM-DETECT(p, M) is $\min(n_{x_0}, |B \cap M_{x_0}|)$. Because $B \subseteq M_{x_0} = M$, this is $\min(n_{x_0}, |B|)$. So NUM-DETECT is effectively the number of sequences covered by p that must still be covered to achieve the coverage constraint.

Furthermore, if sequences are grouped, note that line 3 of Algorithm 3 instead iterates until achieving the desired coverage for each group.

A recent paper[16] on submodular optimization looks at a similar problem; it refers to the groupings in this problem as ground sets and provides an approximation ratio given by the greedy algorithm.

Note on practicality

As with our maximization objective, we applied this minimization objective to design species-specific detection assays, including amplification primers and Cas13a guides, for the 1,933 viral species known to infect vertebrates. We sought to minimize the number of guides subject to detecting >98% of genomes with high activity (Methods). We obtain few guides for most species, but 40 species require more than 3 guides (Supplementary Fig. 20b) and, in one extreme case, as many as 73 (Enterovirus B). Thus, depending on the particular constraints and species, an assay may not be practical with this objective function.

Supplementary Note 3

This note describes an overview of the challenge of evaluating specificity and two formulations, implemented in ADAPT, for doing so. For designs and analyses in this paper, we use the formulation in Exact trie-based search for probe near neighbors.

3a Overview

In applications where differentially identifying a taxonomy is important, ADAPT ensures that the probes it constructs are specific to the taxonomy they are designed to detect. In general, the probes directly perform detection; thus, their specificity is ADAPT's focus, rather than other aspects of a design, such as primers.

The framework for this is as follows. Initially, ADAPT constructs an index of probes across all input taxonomies, which includes the taxonomies and particular sequences containing each probe. This index could also include background sequence to avoid, such as the human transcriptome, although we generally do not include non-viral background sequence. Then, when designing a probe for a taxonomy t_i with genomes S_i, ADAPT queries this index to determine its specificity against all sequences from any S_j for $j \neq i$—namely, to find hits within a specified number of mismatches of the query. The results inform whether the probe might detect some fraction of sequence diversity in t_j. Typically, ADAPT deems a probe to be non-specific if the query yields hits in at least 1% of the sequences from another taxon. ADAPT performs this query while constructing the ground set, as described in Supplementary Note 2.

This problem is computationally challenging. When querying, we generally wish to tolerate a high divergence within a relatively short query to be conservative in finding potential non-specific hits—e.g., up to ~5 mismatches within 28 nt. Also, G-U wobble base pairing (described below) generalizes the usual alphabet of matching nucleotides. Together, these challenges mean that popular existing approaches, including seed/MEM techniques, are not fully adequate for performing queries.

3b G-U wobble base pairing

Some detection applications (e.g., CRISPR-Cas13) rely on RNA-RNA binding. That is, the probe we design is synthesized as RNA and the target is RNA as well. RNA-RNA base pairing allows for more pairing possibilities than with DNA-DNA. In particular, `G` may bind with `U`, forming a *G-U wobble base pair*. It has similar thermodynamic stability to the usual Watson-Crick base pairs[17].

In our Cas13a dataset, we find that U-g mismatches (`U` in the target, `G` in the guide RNA spacer) preserves high activity across guide-target pairs (i.e., when 2 of 2 mismatches or 3 of 3 mismatches are U-g, activity is less likely to be reduced), but we do not observe this same effect for G-u mismatches (Extended Data Fig. 6c). Both U-g and G-u wobble pairings might be tolerated for binding, but the resulting geometries of the pairings could affect Cas13a nuclease activation in different ways. Nevertheless, treating both U-g and G-u pairs (collectively, G-U) as comparable to Watson-Crick base pairs would lead to designs at least as specific as if we were to only do so for U-g pairs. It also permits our algorithms to be tolerant of G-u pairs if other applications, with different enzymatic processes, tolerate those pairs well.

In ADAPT, we wish to treat G-U base pairs as matching when querying for a probe's specificity. For simplicity, here we will use `T` instead of `U` (the RNA nucleobase `U` replaces the DNA nucleobase

T), and thus we consider G-T base pairing. In particular, we consider a base $g[i]$ in a probe to match a base $s[i]$ in a target sequence if either (a) $g[i] = s[i]$, (b) $g[i] =$ A and $s[i] =$ G, or (c) $g[i] =$ C and $s[i] =$ T. (We synthesize the reverse complement of g and use that for detection, so these rules correspond to permitting G-T base pairing.) Note that activity models in ADAPT that are trained for a particular detection technology could prune the query results if the effect is different in some application.

Tolerating G-U base pairing considerably complicates the problem for several reasons. The addition of G-U base pairing raises the probability of a matching hit between a 28-mer and an arbitrary target, thereby expanding the space of potential query results. It also means the Hamming distance between a query and valid hit (considered in the same frame) can be as high as 100%. Yet accommodating this challenge is important in practice to avoid cross-reactivity: ignoring G-U pairing when designing viral species-specific probes can result in missing nearly all off-target hits and deciding many probes to be specific to a viral species when they likely are not (Supplementary Fig. 15).

A similar challenge arises in determining off-target effects when designing small interfering RNA (siRNA)[18,19]. It is common to ignore the problem (e.g., using BLAST to query for off-targets)[20–23]. Other approaches do address it. One is to treat G-U pairs like a mismatch, albeit not as heavily penalized as a Watson-Crick mismatch[24]; however, with this approach, searching for candidate hits may fail to find valid hits if the Hamming distance between the query and hit is sufficiently high owing to G-U pairs. Another approach uses the seed-and-extend technique where the seed is in a well-defined "seed region" that requires an exact match, tolerating G-U pairs in the seed[25]; although applicable to siRNA, a seed-based approach may fail to generalize if there is no seed region, if it is too short, or if it is not consistent or is tolerant of mismatches. For some RNA interference applications, G-U pairs may be detrimental to the activity of an enzyme complex[26], and therefore it may not be necessary to fully account for it when determining specificity. None of these approaches are fully satisfying in ADAPT.

To approach the challenge of G-U wobble base pairing, at several points in the algorithms below we use a transformed sequence (Extended Data Fig. 7a). We transform a probe g into g' by changing A to G and changing C to T; in g', the only bases are G and T. Likewise, we do this for a target sequence s. This is useful because any G-T matching between s and the complement of g is not reflected by different letters between g' and s'—i.e., if the reverse complement of g (what we synthesize) matches with s up to G-U base pairing, then g' and s' are equal strings.

3c Probabilistic search for probe near neighbors

To permit queries for specificity, we first experimented with performing an approximate near neighbor lookup (i.e., one that may miss hits) similar to the description in ref. 27 for points under the Hamming distance. Here, we wish to find probes that are $\leq m$ mismatches from a query.

The approach precomputes a data structure $H = \{H_1, H_2, \ldots, H_L\}$ where each H_i is a hash table that has a corresponding locality-sensitive hash function h_i, which samples b positions of a probe. The h_is bear similarity to the concept of spaced seeds[28]. It chooses L to achieve a desired reporting probability r:

$$L = \lceil \log_{1-P^b}(1-r) \rceil,$$

where $P^b = (1 - m/k)^b$ is a lower bound on the probability of collision (for a single h_i) for nearby probes of length k. In ADAPT, we have used $r = 0.95$ and $b = 22$. For all probes g across all sequences in all taxa t_j, each $H_i[h_i(g')]$ stores $\{(g, j)\}$ where j is an identifier of a taxon from which g

arises and g' is g in the two-letter alphabet described above. Additionally, the data structure holds a hash table G where $G[(g, j)]$ stores identifiers of the sequences in j that contain g. From these data structures, queries are straightforward. For a probe q to query, the query algorithm looks up q' in each H_i and check if q detects (is within m mismatches) each resulting g. For the ones that it does detect, G provides the fraction of sequences in each taxon containing g and therefore provides the fraction of sequences in each taxon that q detects. The algorithm deems q specific iff this fraction is sufficiently small. Note that, when designing probes for a taxon t_j, it is straightforward to mask j from each H_i; this is important for query runtime because most near neighbors would be from j.

This approach would be suitable if we were to not have to consider G-U base pairing, but we found that this consideration makes it too slow to be practical for many applications. To accommodate G-U base pairs, it stores two-letter transformed probes (g') and likewise queries transformed probes (q'). The dimensionality reduction enables finding hits within $\leq m$ mismatches of a query q, sensitive to G-U base pairs, but it also means that most results in each $H_i[h_i(q')]$ are far from q. As a result, the algorithm spends most of its time validating each of these results by comparing it to q. A higher choice of b can counteract this issue, but results in higher L and thus requires more memory. Also, the approach is probabilistic and may fail to detect non-specificity; while a reporting probability might be high per-taxon, when amplified across designing for many taxa the approach becomes more likely to output a non-specific assay for some taxon. Thus, below, we develop an alternative approach that is more tailored to the particular challenges we face.

3d Exact trie-based search for probe near neighbors

Here we describe a data structure and query algorithm that permits queries for non-specific hits of a probe. Unlike the probabilistic approach above, this approach is exact and will always detect non-specificity if present. Having one trie containing all the indexed probes would satisfy the goal of being fully accurate because we could branch, during a query, for mismatches and G-U base pairs; however, the extensive branching involved means that query time would depend on the size of the trie and may be slow (Supplementary Fig. 16). To alleviate this, we place (or *shard*) the probes across many smaller tries.

Briefly, the data structure stores an index of all probes across the input sequences from all taxa. Let k be the probe length (e.g., 28). The data structure splits each probe into p partitions (without loss of generality, assume p divides k). Each partition maps to a $\frac{k}{p}$-bit *signature* such that any two matching strings map to the same signature, tolerating G-U base pairing; each bit corresponds to a letter from the two-letter alphabet described in G-U wobble base pairing. There are $p \cdot 2^{k/p}$ tries in total, each associated with a signature and a partition, and every probe is inserted into p tries according to the signatures of its p partitions.

To query a probe q, the algorithm relies on the pigeonhole principle: tolerating up to m mismatches across all of q, there will be at least one partition with $\leq \lfloor m/p \rfloor$ mismatches against each valid hit. For each partition of q, the query algorithm produces all combinations of signatures within $\lfloor m/p \rfloor$ mismatches—there are $\sum_{i=0}^{\lfloor m/p \rfloor} \binom{k/p}{i}$ of them—and looks up q in the tries with these signatures for the partition. During each lookup, it branches to accommodate G-U base pairing and up to m mismatches. Note that the bit signature tolerates G-U base pairing—i.e., two positions have the same bit if they might be a match, including owing to G-U pairing—so the algorithm finds all hits, even if the query and hit strings diverge due to G-U pairing.

Extended Data Fig. 7 provides a visual depiction of building the data structure and performing queries, and Algorithms 4 and 5 provide pseudocode.

Algorithm 4 Build data structure of tries to support specificity queries.

Input

$\{S\}$ collection of sequences across taxonomies
k probe length
p number of partitions

Output

$\mathcal{T}$ space of tries indexing probes

function Build-Tries($\{S\}$, k, p)
  Initialize $\mathcal{T}$ ▷ contains $p \cdot 2^{k/p}$ tries, one per pair of partition and bit vector
  for each taxonomy t_i **do**
    $S_i \leftarrow$ Sequences for t_i
    for each k-mer (probe) g in S_i **do**
      for $r = 1$ **to** p **do**
        $g_r \leftarrow$ Partition r of g
        $g'_r \leftarrow$ Hash of g_r: `A` $\rightarrow 0$, `G` $\rightarrow 0$, `C` $\rightarrow 1$, `T` $\rightarrow 1$ ▷ bit vector
        $T \leftarrow$ Trie in $\mathcal{T}$ corresponding to partition r and bit vector g'_r
        Insert g into T ▷ include t_i and sequence identifier in leaf node
  return $\mathcal{T}$

A loose bound on the runtime of a query is

$$O\left(p \cdot \frac{n}{2^{k/p}} \cdot \sum_{i=0}^{\lfloor m/p \rfloor} \binom{k/p}{i}\right)$$

where n be the total number of probes indexed in the data structure. The query algorithm performs a search for p partitions of a query q. For each partition, it considers $\sum_{i=0}^{\lfloor m/p \rfloor} \binom{k/p}{i}$ tries, one for each combination of $\lfloor m/p \rfloor$ bit flips. The size of each trie is a loose upper bound on the query time within it; assuming uniform sharding, the size of each is $O(\frac{n}{2^{k/p}})$. Multiplying the size of each trie by the number of them considered during a query provides the stated runtime. Adjusting p, a small constant, allows us to tune the runtime: higher choices reduce the number of bit flips, and thus the number of tries to search, but yield larger tries, and thus requires more time searching within each of them. The runtime does not scale well with our choice of m, but this is generally a small constant (up to $\sim$5). The term providing the worst-case query time within each trie, $O(\frac{n}{2^{k/p}})$, is likely to be a considerable overestimate in practice because queries usually do not need to fully explore a trie.

Because the data structure stores each probe in p separate tries, the required memory is $O(np)$. Although this scales reasonably with n, it involves large constant factors and is memory-intensive in practice; one future direction is to compress the tries.

Algorithm 5 Query tries to find non-specific hits.

Input

q	probe to query for specificity to taxon t_i
m	number of mismatches to tolerate (counting G-U pairs as matches)
p	number of partitions

Requires: $\mathcal{T}$ from Build-Tries
Requires: taxon t_i is masked from $\mathcal{T}$
Output

G	taxon and sequence identifiers of non-specific hits

function Query(q, m, p)
    $G \leftarrow \{\}$
    for $r = 1$ **to** p **do**
        $q_r \leftarrow$ Partition r of q
        $q'_r \leftarrow$ Hash of q_r: `A` $\rightarrow 0$, `G` $\rightarrow 0$, `C` $\rightarrow 1$, `T` $\rightarrow 1$ ▷ bit vector
        for each variant $(q'_r)'$ of q'_r with $\leq \lfloor m/p \rfloor$ flipped bits **do**
            $T \leftarrow$ Trie in $\mathcal{T}$ corresponding to partition r and bit vector $(q'_r)'$
            $g \leftarrow$ Query results for q in T, branching always for G-U
                pairing and for up to m mismatches
            $G \leftarrow G \cup \{g\}$
    return G

Supplementary Note 4

This note describes how we link methods, from Supplementary Notes 2 and 3, in ADAPT to form an end-to-end system for designing assays. In particular, this involves identifying amplification primers, searching across genomic regions, and connecting with publicly available genome databases.

4a Identifying amplification primers

In many nucleic acid applications, we must amplify a genomic region to obtain enough material for detection. For example, the CRISPR-based detection platforms SHERLOCK[29] and DETECTR[30] use an isothermal approach, recombinase polymerase amplification (RPA), to amplify a target region; then, probes (in these applications, CRISPR guide RNAs) allow for target detection. Thus, the probes in a probe set P ought to be within a genomic region of the alignment that is bound by suitable primers to amplify the region (Supplementary Note Fig. 1).

In contrast to probes, our search for primers is related to a conventional approach that targets conserved regions and employs amplification-method–specific heuristics to filter primers. We identify suitable primers at every position of the alignment S by approximating a minimal set of primers that achieves a desired coverage over the input genomic variation. In particular, we run Algorithm 3 in Supplementary Note 2 at every site in the genome, except parameterized for primers. The parameters include primer length, number of tolerated mismatches, the fraction of genomes that must be covered, as well as bounds on GC content used as a filter; their values can be tuned based on a particular amplification method. See Methods for the particular parameter values that we use with ADAPT in practice, which we chose according to published recommendations for RPA[31].

4b Branch and bound search for genomic regions

In ADAPT, we perform a search for genomic regions to target (which may, optionally, include amplification primers) simultaneously with optimizing the probe objectives that are described in Supplementary Note 2. As with probes, we want to penalize the number of primers required to amplify a region because they can interfere with each other or require multiple reactions. Similarly, we wish to penalize the length of the region because longer regions are less efficient to amplify; penalizing the logarithm of length approximates the length-dependence of amplification efficiency. We first walk through the search using the objective that maximizes expected activity (Supplementary

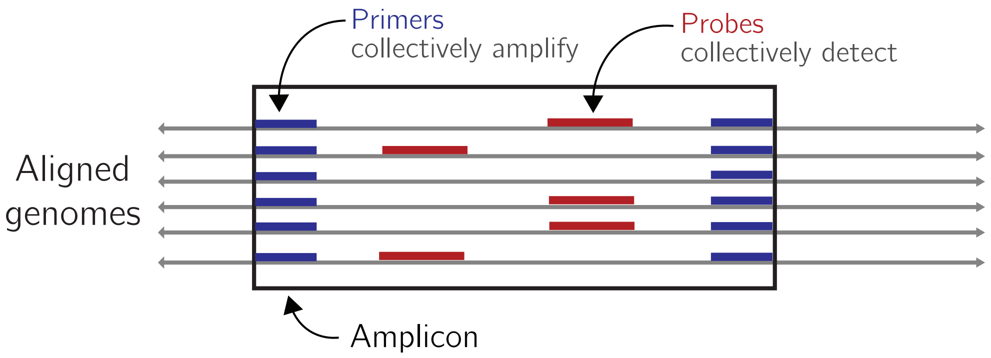

Supplementary Note Figure 1 — Searching for genomic regions. ADAPT searches for a region of the genome, bound by conserved sequence to use for primers, that contains probes that can collectively detect the region. The requirement that a region be bound by conserved sequence and represent an amplicon is optional.

Note 2a). For this, we now perform a search for a genomic region R that encompasses the probe set P and solve

$$\max_{P,R} \left\{ \widetilde{F}(P) - \lambda_A |R_A| - \lambda_L \log(R_L) : |P| \leq \overline{m_p} \right\}$$

where $\widetilde{F}(P)$ and $\overline{m_p}$ are defined in Supplementary Note 2a, R_A gives the set of primers bounding the region, R_L gives the nucleotide length of the region, and λ_A and λ_L give weights on the penalties. Note that λ_A and λ_L can optionally be set to 0, removing the requirement that a region be bound by conserved sequence and represent an amplicon. We typically add a fixed constant (4) to the objective values before reporting them to the user, which we find makes the values more interpretable to users because it makes them more likely to be non-negative; this shift has no impact on the design options or their rankings.

To solve this, we use an algorithm in which we search over options for R and prune unnecessary ones (Supplementary Note Fig. 2). Rather than finding a single maximum, we wish to compute the highest N solutions—i.e., N regions, each containing a probe set—to the objective. This is important so that multiple design options can be tested and compared experimentally; it also provides the option for an assay to target multiple regions in a genome. Note also that the range of $\widetilde{F}$ has an upper bound, which we call $\widetilde{F}_{\text{hi}}$, calculated from $F(P)$'s highest value (predicted activities are bounded) and $|P| = 1$.

We maintain a min heap h of the N designs with the highest value of the objective. First, we identify a primer set at every position in the alignment, as described in Identifying amplification primers. Then, we search over pairs of positions in the alignment, considering the regions that would be amplified by primers at each pair. Although the number of such regions is quadratic in the alignment length, we can effectively prune regions based on $|R_A|$ and R_L. We calculate, with these values, the objective value using $\widetilde{F}_{\text{hi}}$ in place of $\widetilde{F}(P)$; this value provides an upper bound on the solution. If this value falls below that of the minimum in h, the region cannot be in the top N and thus we do not need to compute P. For regions that could be in the best N, we compute the probe set P with the maximal $\widetilde{F}(P)$ as described in Supplementary Note 2a (Solving for P). If the objective value for the design given by (R, P) is greater than the minimum in h, we pop from h and push the design to it. This search identifies the top N regions according to the objective, up to our approximation of $\widetilde{F}(P)$.

This search follows the branch and bound paradigm in which the candidate solutions (R, P) make up a 3-level tree, excluding the root. The levels represent the (1) $5'$ primers, (2) $3'$ primers, and (3) probe set P. We can prune the choice of $3'$ primers based on the length of the amplicon they would form. Exploring the final level in particular—determining P—is the slow step. Since we can easily construct an upper bound on the candidate solution for nodes in the final level, which we compare to the minimum in h, we can discard nodes and thus avoid having to compute P for many candidate solutions.

It is also important that the design options are diverse, i.e., reflect meaningfully different regions rather than being simple shifts of one another. To account for this, we implement the following: if a design to push to h has a region overlapping that of an existing design in h, it must replace that existing design (and only does so if the new one has a higher objective value).

Additionally, during our search many of the computations—particularly when computing probe sets—would be performed repeatedly from the same input, owing to overlap between different regions across the search. As a result, we memoize results of these probe set computations according to genome position. A branch and bound implementation, as described above, might start with all

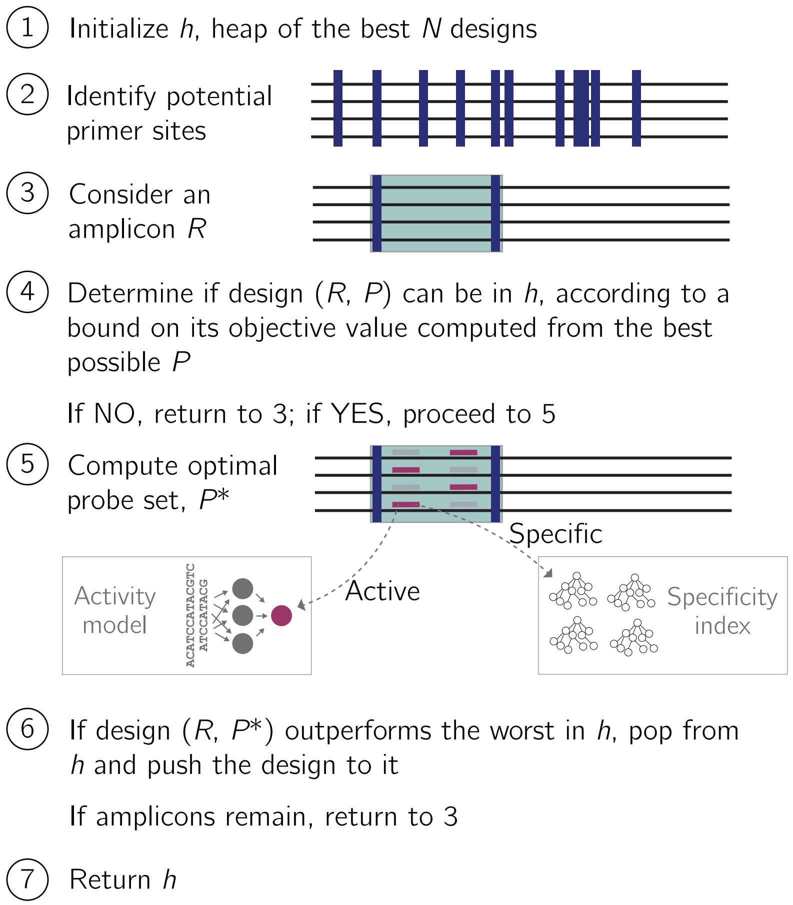

Supplementary Note Figure 2 — Branch and bound search for genomic regions. Sketch of the search for genomic regions (amplicons) and optimal probe sets within them. An amplicon R includes information about the primers used for amplifying it. Supplementary Note 2 describes the algorithms in Step 5. Step 5 makes use of the predictive activity model and the data structure (Supplementary Note 3) for evaluating specificity.

the $5'$ primers (first level) and then select the best $3'$ primers (second level), before advancing to computing probe sets. However, this could force each successive probe set computation to jump to a different region in the genome, as defined by the primer pairs: we would not be able to efficiently cleanup memoizations for these computations and memory would grow throughout the search. To avoid this issue, we scan linearly along the genome and, each time we advance the $5'$ primer position, we determine probe set memoizations that we no longer need to store. Memoizing these computations provides a considerable improvement in runtime (Supplementary Fig. 17).

The above description applies to maximizing expected activity, but it is straightforward to adjust the strategy when minimizing the number of probes (Supplementary Note 2b). In this case, we change our objective to solve for

$$\min_{P,R} \{|P| + \lambda_A |R_A| + \lambda_L \log(R_L)\}$$

where we also impose the constraint on coverage described in Supplementary Note 2b. The search now stores a max heap h of the designs with the smallest values of the objective. For pruning, we compute a lower bound on the candidate solution by letting $|P| = 1$, and compare this bound to the maximum in h.

The search is embarrassingly parallel. One future direction is to parallelize the search across genomic

regions, in which we perform it separately for contiguous parts of the genome and then merge the resulting heaps. In practice, the primary challenge is likely to be handling shared memory, in particular for the large index used to enforce specificity.

4c Fetching and curating sequences to target

ADAPT accepts a collection of taxonomies provided by a user: $\{t_1, t_2, \ldots\}$. It can either design for one t_i or for all t_i, in either case ensuring designs are specific accounting for all t_j where $j \neq i$. Each t_i generally represents a species, but can also be a subspecies taxon. In NCBI's databases, each taxonomy has a unique identifier[32] and ADAPT accepts these identifiers. ADAPT then downloads all near-complete and complete genomes for each t_i from NCBI's genome neighbors database, but uses its Influenza Virus Resource database[33] for influenza viruses. It also fetches metadata for these genomes (e.g., date of sample collection), which some downstream design tasks process. (Many species have segmented genomes. For these, ADAPT also needs the label of the segment. ADAPT effectively treats each segment as a separate taxonomy—i.e., for species that are segmented, the t_is are actually pairs of taxonomy ID and segment.)

We must then prepare these genomes for design. Briefly, for each t_i we curate the genomes by aligning each one to one or more reference sequences for t_i and removing genomes that align very poorly to all references, as measured by several heuristics: by default, we remove a genome that has $< 50\%$ identity to all references or that have $< 60\%$ identity to all references after collapsing consecutive gaps to a single gap. (The "reference" sequences are determined by NCBI, but can also be provided by the user; they are manually curated, high-quality genomes and encompass major strains.) This process prunes genomes that are misclassified, have genes in an atypical sense, or are highly divergent for some other reason. Then, we cluster the genomes for t_i with an alignment-free approach by computing a MinHash signature for each genome, rapidly estimating pairwise distances from these signatures (namely, the Mash distance[34]), and performing hierarchical clustering using the distance matrix. The default maximum cophenetic distance (approximate average nucleotide dissimilarity) for clustering is 20%. In general, we obtain a single cluster for a species (Supplementary Fig. 20f). This provides another curation mechanism, because it can discard clusters that are too small (by default, just one sequence). Finally, ADAPT aligns the genomes within each cluster using MAFFT[35]. This yields a collection of alignments, where each is for a cluster of genomes from taxon t_i.

Many of these computations—such as curation, clustering, and alignment—are slow yet are repeated on successive runs of ADAPT. ADAPT memoizes results of the above computations, to disk, to reuse on future runs when the input permits it. This memoization to disk improves the runtime for routine use of ADAPT. We use it when reporting computational requirements.

4d Computational requirements in practice

We recorded computational requirements when using ADAPT to design maximally active, species-specific diagnostic assays for the 1,933 viral species known to infect vertebrates. Fig. 4e shows runtime for end-to-end design; runtime depended in part on the number of genome sequences. ADAPT required about 1 to 100 GB of memory per species, with one family's species requiring considerably more than others (Supplementary Fig. 20c); these memory requirements may necessitate using cloud computing or similar services.

After curating available genome sequences, ADAPT considered all or almost all genome sequences for most species (Supplementary Fig. 20d–f), including ones with >1,000 sequences. It retained

fewer than half of sequences during curation for 38 species; that might be appropriate (e.g., if many sequences are misclassified) but requires further investigation.

Enforcing species-specificity imposes a considerable computational burden on ADAPT, as expected, adding to its runtime and memory usage (Supplementary Fig. 22a,b) while decreasing the solution's activity and objective value because of the added constraints (Supplementary Fig. 22c,d). However, the effect is tunable: relaxing the stringency of these specificity constraints can greatly decrease the required computational resources, such as memory usage, which could be helpful for some users.

Supplementary Note 5

This note describes our approach to evaluate probe activity over time by forecasting relatively likely substitutions. Results are in Extended Data Fig. 8.

5a Motivation

Nucleic acid diagnostics are susceptible to degraded performance as viral genomes accumulate substitutions. Extensive genomic data can inform where to design probes, for example, by identifying regions with less variability or regions where a probe (e.g., Cas13a guide) can maintain high activity across variation. However, finding such sites is difficult or impossible when there is little genomic data, as is the case early in an outbreak of a novel virus or for an understudied virus.

One option is to use a substitution model to simulate likely types of substitutions in the genome, and then to predict activity against these simulated sequences. Parameters of the model can be transferred from related viruses. The approach would account for the possibility that some potential target regions are more likely to accrue mutations that degrade activity than other regions. For example, consider a region rich in `T` nucleotides—complementary probes have `A`—and a virus with a high relative rate of `T` to `C` transitions. Also, consider a case where `A-C` probe-target mismatches harm activity, particularly in a specific location of the probe. Such substitutions in the genome would induce those mismatches; simulating these substitutions could inform ADAPT to avoid the regions or to position probes to avoid this type of potential mismatch.

5b Background on substitution model

We use the general time-reversible (GTR) model, which is based on a continuous-time Markov process that accounts for relative rates of substitutions. Ref. 36 contains further information, and this subsection summarizes the model. The parameters of this model are the equilibrium base frequencies, $(\pi_A, \pi_C, \pi_G, \pi_T)$ and the rate parameters, $r_{AC}, r_{AG}, r_{AT}, r_{CG}, r_{CT}, r_{GT}$. Note that $r_{ij} = r_{ji}$. For convenience, denote the above parameters as a, b, c, d, e, f respectively.

The GTR model includes a rate matrix $Q = \{q_{ij}\}$, giving the instantaneous rate at which a base $i \in \{\mathrm{A, C, G, T}\}$ changes to $j \in \{\mathrm{A, C, G, T}\}$:

$$Q = \begin{pmatrix} \cdot & a\pi_C & b\pi_G & c\pi_T \\ a\pi_A & \cdot & d\pi_G & e\pi_T \\ b\pi_A & d\pi_C & \cdot & f\pi_T \\ c\pi_A & e\pi_C & f\pi_G & \cdot \end{pmatrix}$$

The diagonal entries are set so that each row sums to 0 and it is typical to normalize Q so that the average rate is 1 (i.e., $-\sum_{i=1}^{4} \pi_i Q_{ii} = 1$).

The GTR model specifies a transition probability matrix P, computed numerically via matrix exponentiation:

$$P(t) = e^{Q \cdot \mu \cdot t} = \begin{pmatrix} p_{\mathrm{AA}}(t) & p_{\mathrm{AC}}(t) & p_{\mathrm{AG}}(t) & p_{\mathrm{AT}}(t) \\ p_{\mathrm{CA}}(t) & p_{\mathrm{CC}}(t) & p_{\mathrm{CG}}(t) & p_{\mathrm{CT}}(t) \\ p_{\mathrm{GA}}(t) & p_{\mathrm{GC}}(t) & p_{\mathrm{GG}}(t) & p_{\mathrm{GT}}(t) \\ p_{\mathrm{TA}}(t) & p_{\mathrm{TC}}(t) & p_{\mathrm{TG}}(t) & p_{\mathrm{TT}}(t) \end{pmatrix}$$

where $p_{ij}(t)$ is a probability that i transitions to j after elapsed time t. The overall substitution rate is μ and $\mu \cdot t$ has units of expected number of substitutions per site. We use P, below, to simulate substitutions.

In analyses in Extended Data Fig. 8, we used $\mu = 10^{-3}$ substitutions/site/year and $t = 5$ years. We also empirically computed base frequencies from the SARS-CoV-2 genome used for those analyses and used the following relative rates (normalized to r_{GT}), which we estimated: $r_{AC} = 1.3$, $r_{AG} = 5.4$, $r_{AT} = 1.8$, $r_{CG} = 0.8$, $r_{CT} = 9.5$, and $r_{GT} = 1.0$.

More sophisticated models—such as ones that accommodate rate variation among sites—may perform better for this task, but we have not experimented with them.

5c Evaluating probes against simulated sequences

Let s be the sequence of a virus at a given time, and S_t be a discrete random variable representing the sequence of the virus after time t has elapsed. The above probability matrix P allows us to compute $\Pr(S_t = \bar{s} | s, t, P)$, the likelihood of sequence $\bar{s}$ given the current sequence s. We use this model to construct a distribution of potential sequences after time t, and assess our probe's activity against these sequences using our predictive model (namely, for Cas13a guides). For each pair of a probe g and original target sequence s_i, we can obtain a sampling of activities,

$$\{d(g, s_{i,1}), \ldots, d(g, s_{i,n})\},$$

where each $s_{i,j}$ is one of n simulated sequences and $d(g, s_{i,j})$ is a predicted detection activity.

Algorithm 6 Estimate probe activity against simulated sequences.

Input

$\{s_i\}$	collection of target sequences for taxon, at and around site where g binds
g	probe sequence
d	activity prediction function
P	transition probability matrix
n	number of sequences to simulate for each original target sequence s_i

Output

mean activity across target genomes

function EVALUATE-PROBE-AGAINST-SIMULATED-SEQUENCES($\{s_i\}$, g, d, P, n)
    $C \leftarrow \{\}$
    for each target sequence s_i in $\{s_i\}$ **do**
        $A_i \leftarrow \{\}$
        for $j \leftarrow 1$ to n **do**
            $s_{i,j} \leftarrow$ Simulate(s_i, P)   ▷ sample substitutions against s_i
            $A_i \leftarrow A_i \cup \{d(g, s_{i,j})\}$ ▷ predict detection activity
        $C \leftarrow C \cup \{$ Bottom-5th-Percentile(A_i) $\}$
    return mean(C)

We are often interested (Extended Data Fig. 8d) in whether a probe maintains activity against most of the potential substitutions—that is, we may want to be "risk-averse" and avoid a situation, even if unlikely, where we observe a drop in activity owing to possible substitutions. For this goal, we use the bottom 5th percentile of the different $d(g, s_{i,j})$ to summarize the activity against simulated sequences originating with each s_i. And then we summarize these across the different s_i by taking the mean. Algorithm 6 shows pseudocode.

Supplementary Figures

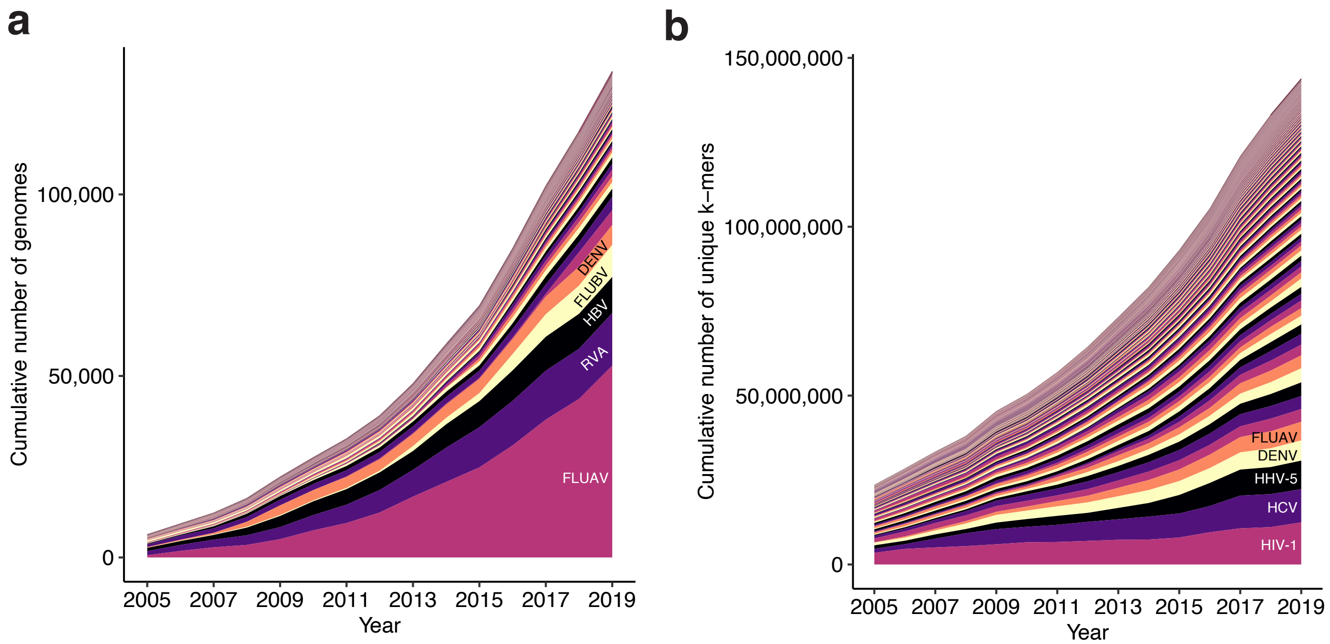

Supplementary Figure 1 — Growing number of viral genomes and diversity. Growth of data over time for 573 viral species known to infect humans. Each species is a color. **a**, Cumulative number of genome sequences, counted from NCBI[37] viral genome neighbor and influenza databases, for each species that were available up to each year. For genomes with multiple segments, this counts only the number of sequences of the segment that has the most sequences. 5 species with the most number of genomes are labeled. FLUAV, influenza A virus; RVA, rotavirus A; HBV, hepatitis B virus; FLUBV, influenza B virus; DENV, dengue virus. **b**, Number of unique 31-mers for the genomes in **a**, a simple measure of diversity. HIV-1, human immunodeficiency virus 1; HCV, hepatitis C virus; HHV-5, human betaherpesvirus 5. In both panels, year indicates the year of the entry creation date in the database.

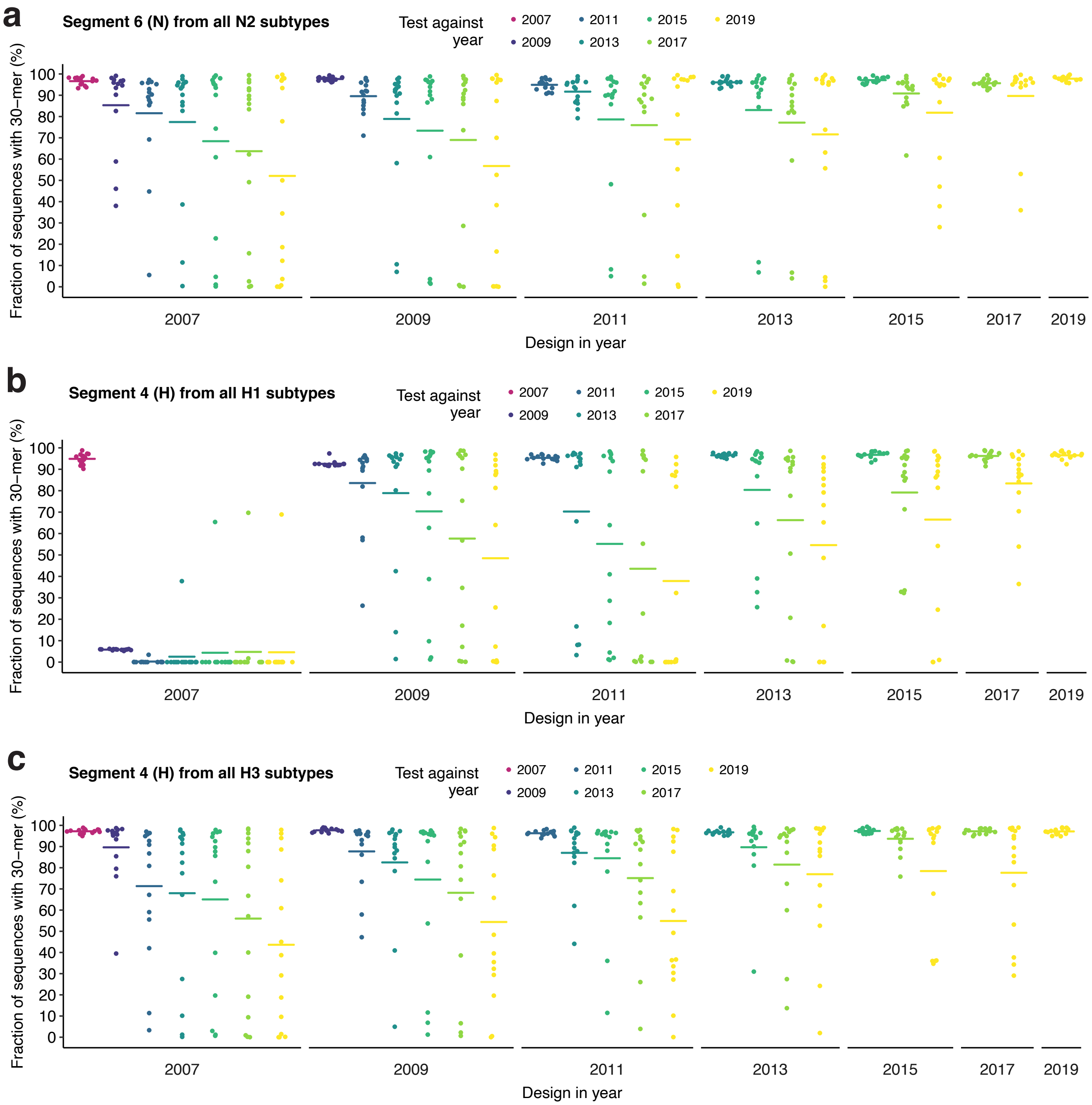

Supplementary Figure 2 — Comprehensiveness of conserved influenza A virus 30-mers over time. Even when considering the most conserved sequences, diagnostic performance of probes can degrade over time owing to genomic changes. At each year, we select the 15 most conserved non-overlapping 30-mers according to recent sequence data up to that year—a simple model for designing diagnostic probes at different years, without any consideration to other constraints such as specificity or activity. Each point represents a 30-mer from the year in which it was designed. We then measure the fraction of all sequences in subsequent years (colored) that contain each 30-mer—a simple test of comprehensiveness. Bars indicate the mean fraction of sequences containing the 15 30-mers at each combination of design and test year. To aid visualization, only odd years are shown. **a**, Segment 6 (N) sequences from all N2 subtypes. **b**, Segment 4 (H) sequences from all H1 subtypes. **c**, Segment 4 (H) sequences from all H3 subtypes. Extended Data Fig. 1a shows segment 6 (N) sequences from N1 subtypes.

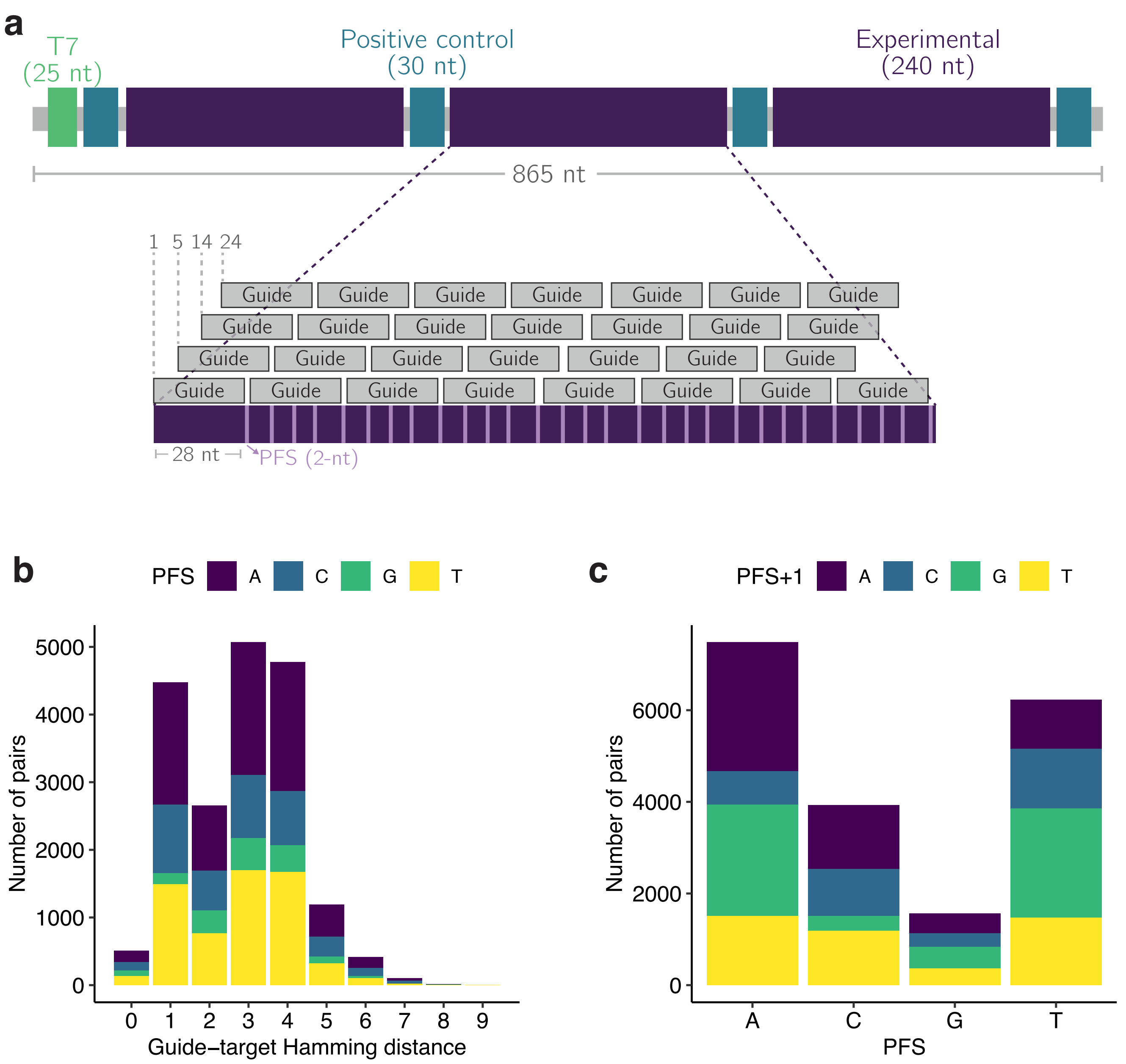

Supplementary Figure 3 — Guide-target library design. **a**, Top, depiction of the wildtype target. The wildtype contains a T7 promoter on the 5′ end for transcription, four positive control regions, and three experimental regions. Each positive control region contains a unique guide that matches perfectly all targets, except negative control targets (not shown). Bottom, zoom of one experimental region from the wildtype target. Guide sequence comes from tiling along this region—29 guides per experimental region. There are also negative control guides (not shown) that only match the negative control targets. Other targets contain mismatches relative to the wildtype target, and thus contain mismatches relative to the guide sequences. **b**, Distribution of the Hamming distance between guide and target across guide-target pairs. Color represents the number of pairs with each protospacer flanking site (PFS) at each Hamming distance. The 19,209 unique guide-target pairs included in our final, curated dataset (Methods) are shown. **c**, Same as **b**, but the distribution of PFS across the guide-target pairs. Color represents the number of pairs with each nucleotide immediately following the PFS (3′ end of protospacer).

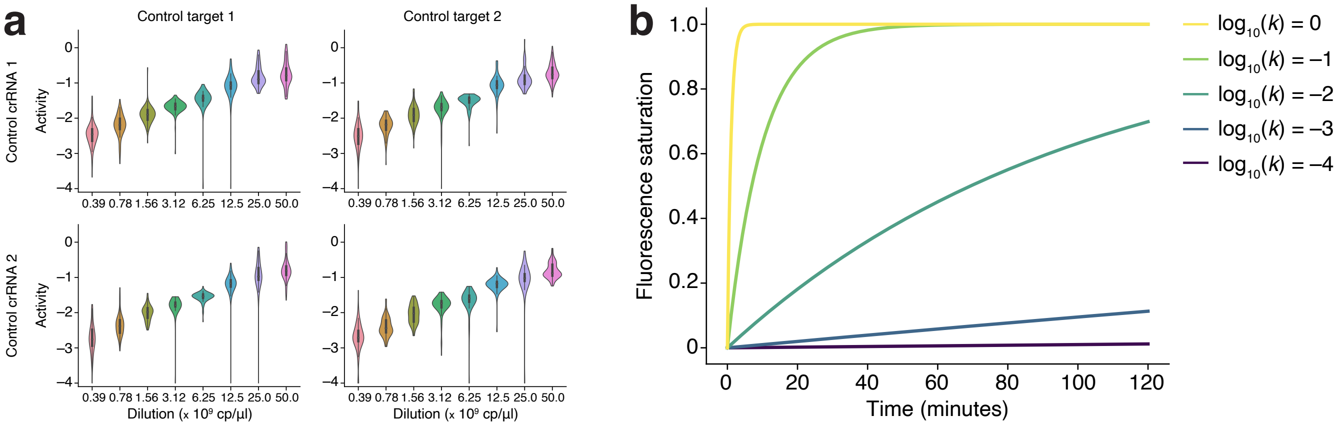

Supplementary Figure 4 — Assessing activity through CRISPR-Cas13a reaction kinetics. **a**, Density and interquartile ranges of Cas13 activity for a series of target concentrations, using two control targets and guides from our Cas13 library. We model fluorescence for each guide-target pair over time (Fig. 1a; Methods), fitting a curve of the form $C(1 - e^{-kt}) + B$ where t is time and e^{-kt} represents remaining reporter presence over time. We take $\log_{10}(k)$ to be the measure of Cas13 activity. The fluorescence growth curve depends on the concentration of the guide-target-Cas13a complex (of which target concentration is the limiting component) and its enzymatic efficiency; in generating our dataset, we hold the complex concentration constant so that activity evaluates enzymatic efficiency. **b**, Theoretical fluorescence saturation over time—namely, the term $1 - e^{-kt}$—for five activity values. Over the time scale of our experiment (t up to $\sim$120 minutes), when k is small we cannot observe reporter activation and the curve is approximately linear, making it difficult to estimate C and k together; these features motivate the use of an activity cutoff. Therefore, we label guide-target pairs with $\log_{10}(k) \leq -4$ as inactive and those with $\log_{10}(k) > -4$ as active.

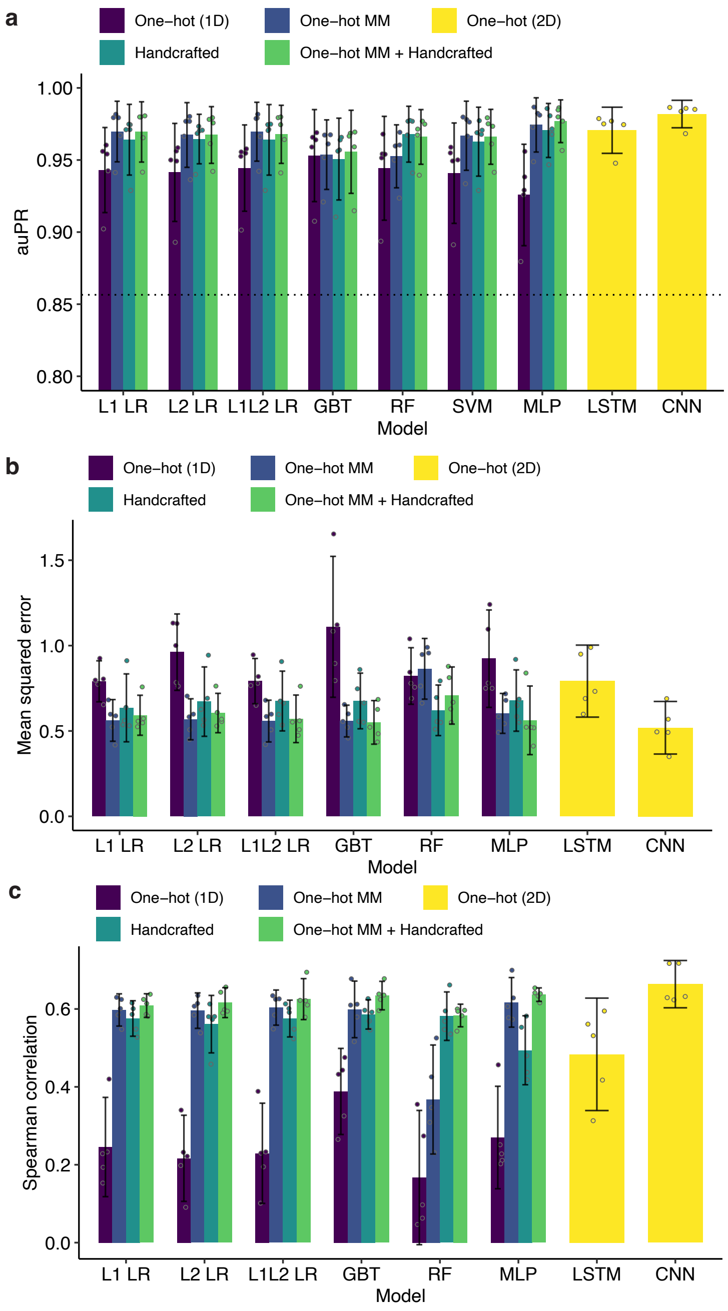

Supplementary Figure 5 — Nested cross-validation for classification and regression. For each model and input type (color) on each of five outer folds, we performed a five-fold cross-validated hyperparameter search. Bar shows the mean of a statistic on the validation data for the $n = 5$ outer folds (each is a point), and the error bar indicates the 95% confidence interval. **a**, Area under precision-recall curve (auPR) for different classification models. auROC is in Fig. 2a. L1 LR and L2 LR, logistic regression; L1L2 LR, elastic net; GBT, gradient-boosted classification tree; RF, random forest; SVM, support vector machine; MLP, multilayer perceptron; LSTM, long short-term memory recurrent neural network; CNN, convolutional neural network including parallel convolution filters of different widths and a locally-connected layer. One-hot (1D) is one-hot encoding of target and guide sequence independently, i.e., without encoding a pairing of nucleotides between the two; One-hot MM is one-hot encoding of target sequence nucleotides and of mismatches in guides relative to the target; Handcrafted is curated features of hypothesized importance (Methods); One-hot (2D) is one-hot encoding of target and guide sequence with encoded guide-target pairing. Dashed line is precision of random classifier (equivalently, the fraction of guide-target pairs that are active). **b**, Mean squared error (MSE) for different regression models (lower is better). L1 and L2 LR, regularized linear regression; L1L2 LR, elastic net; GBT, gradient-boosted regression tree; RF, MLP, LSTM, and CNN are as in **a** except constructed for regression. Input types are as in **a**. **c**, Same as **b** but the statistic is Spearman correlation.

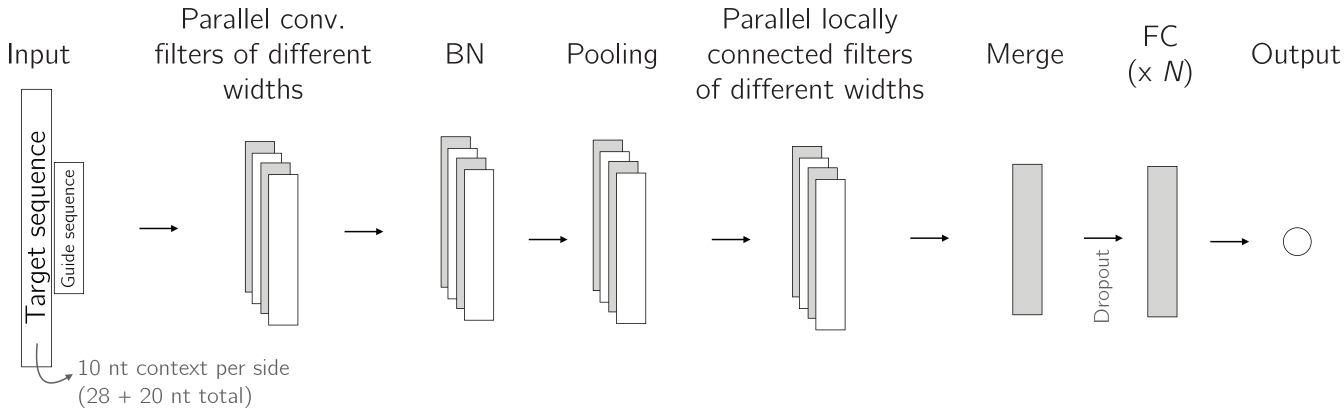

Supplementary Figure 6 — Architecture of convolutional neural network for guide-target activity prediction. Convolutional neural network (CNN) architecture for classifying and regressing activity; hyperparameter search and training is separate for each task. The inputs are one-hot encoded for the target and guide sequences (8 channels together). There are multiple convolutional filters of different widths processing the input in parallel, as well as multiple locally connected filters of different widths; outputs of these different filters are concatenated in the merge layer. Pooling includes maximum, average, and both. 'BN' is batch normalization and 'FC' is fully connected. There are N fully connected layers. The dropout layers are in front of each fully connected layer.

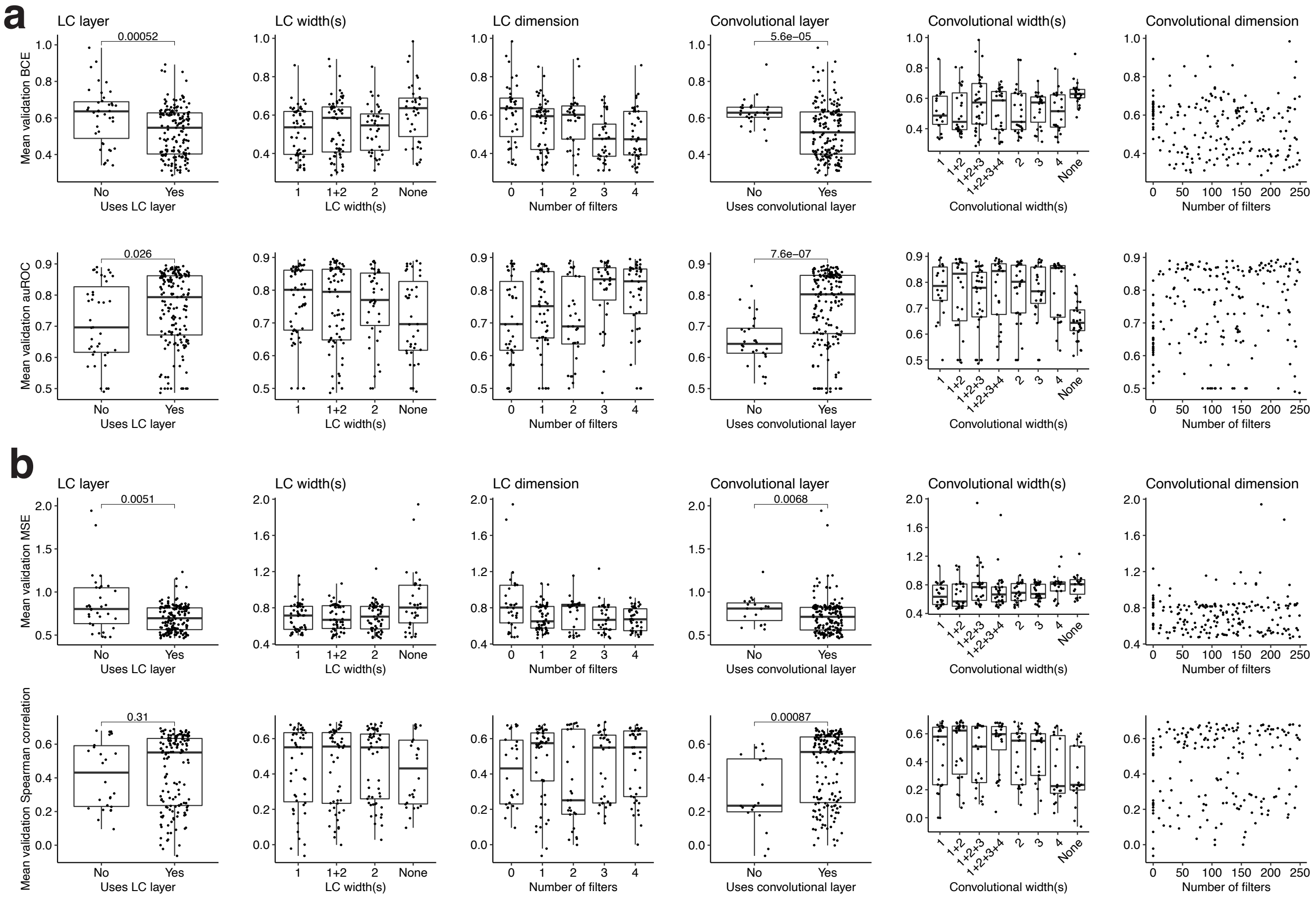

Supplementary Figure 7 — Hyperparameter search for convolutional neural networks. We used a random search over the hyperparameter space (200 draws) to select each convolutional neural network (CNN) model. Each plot corresponds to a hyperparameter and shows choices of that hyperparameter; see Methods for all hyperparameters. The evaluations are cross-validated: each dot indicates the mean of a metric, computed across $n = 5$ folds, for a draw of hyperparameters. Boxes indicate first and third quartiles (25th and 75th percentiles), center bars indicate median, upper whiskers extend to maxima (if points are higher than 1.5 times the interquartile range from the box, then only up to that value), and lower whiskers likewise extend to minima. 'LC', locally connnected. The '+' in LC and convolutional widths separates different widths of parallel filters; 'None' indicates that the model does not use an LC or convolutional layer. *P*-values are computed from Mann-Whitney *U* tests (one-sided). **a**, Results of hyperparameter search for classification. BCE, binary cross-entropy. **b**, Results of hyperparameter search for regression. MSE, mean squared error.

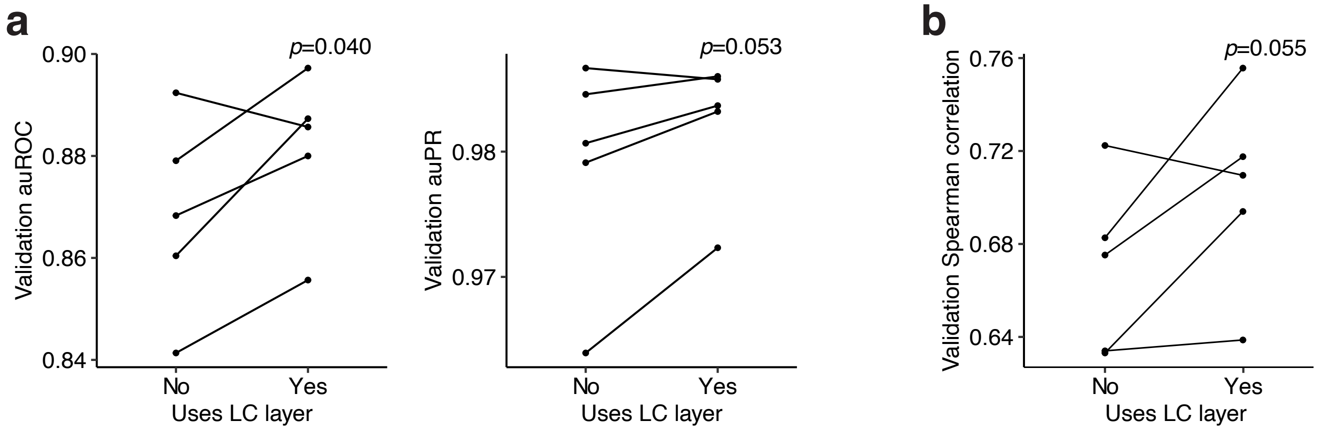

Supplementary Figure 8 — Effect of locally connected layers on model performance. Results of the forced inclusion or exclusion of locally connected layers ('LC'; Supplementary Fig. 6) in convolutional neural networks for Cas13a guide-target activity prediction. We perform nested cross-validation: on each of five outer folds, we perform a five-fold cross-validated hyperparameter search to select a model, once using locally connected layers and once not using them. Plotted values are calculated on the validation data for each of the five outer folds. **a**, auROC and auPR for classifying activity. **b**, Spearman correlation for regressing activity on active guide-target pairs. *p*-values are computed from one-sided paired *t*-tests.

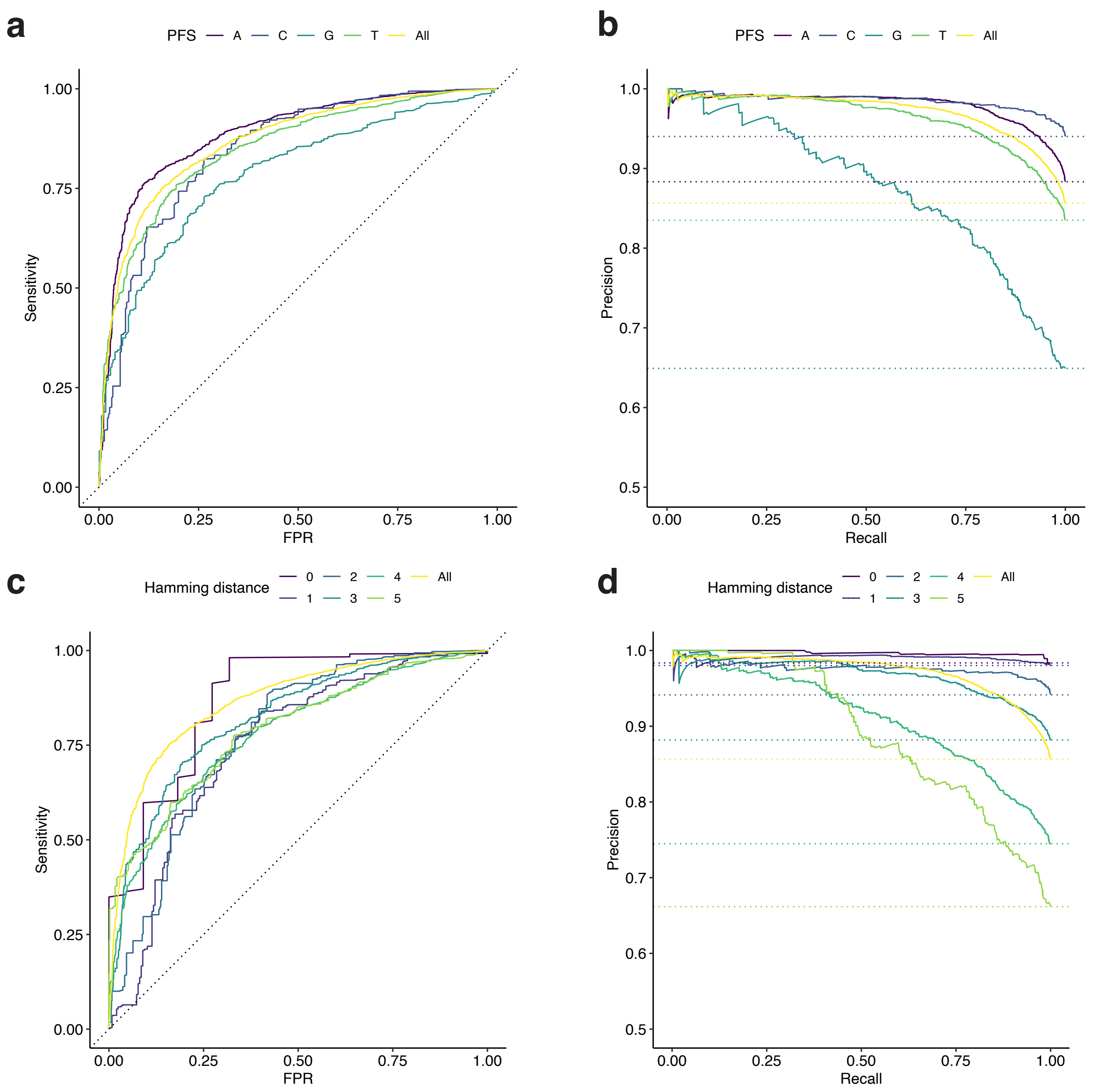

Supplementary Figure 9 — Classification performance on subsets of test data. Evaluations of classification on different subsets of the hold-out test data corresponding to features that considerably affect activity. Here, the model is the same for all evaluations and only tested (not trained) on different subsets. **a**, ROC curves computed from guide-target pairs with the different protospacer flanking site (PFS) nucleotides. **b**, Precision-recall (PR) curves computed from pairs with the different PFS nucleotides. Dashed lines are precision of random classifiers for each PFS (equivalently, the fraction of guide-target pairs that are active with each PFS). **c**, ROC curves computed from pairs with different Hamming distances between guide and target. **d**, PR curves computed from pairs with different Hamming distances between guide and target. Dashed lines are precision of random classifiers for each choice of Hamming distance (equivalently, the fraction of guide-target pairs that are active at each Hamming distance). In all panels, yellow curve is for all test data.

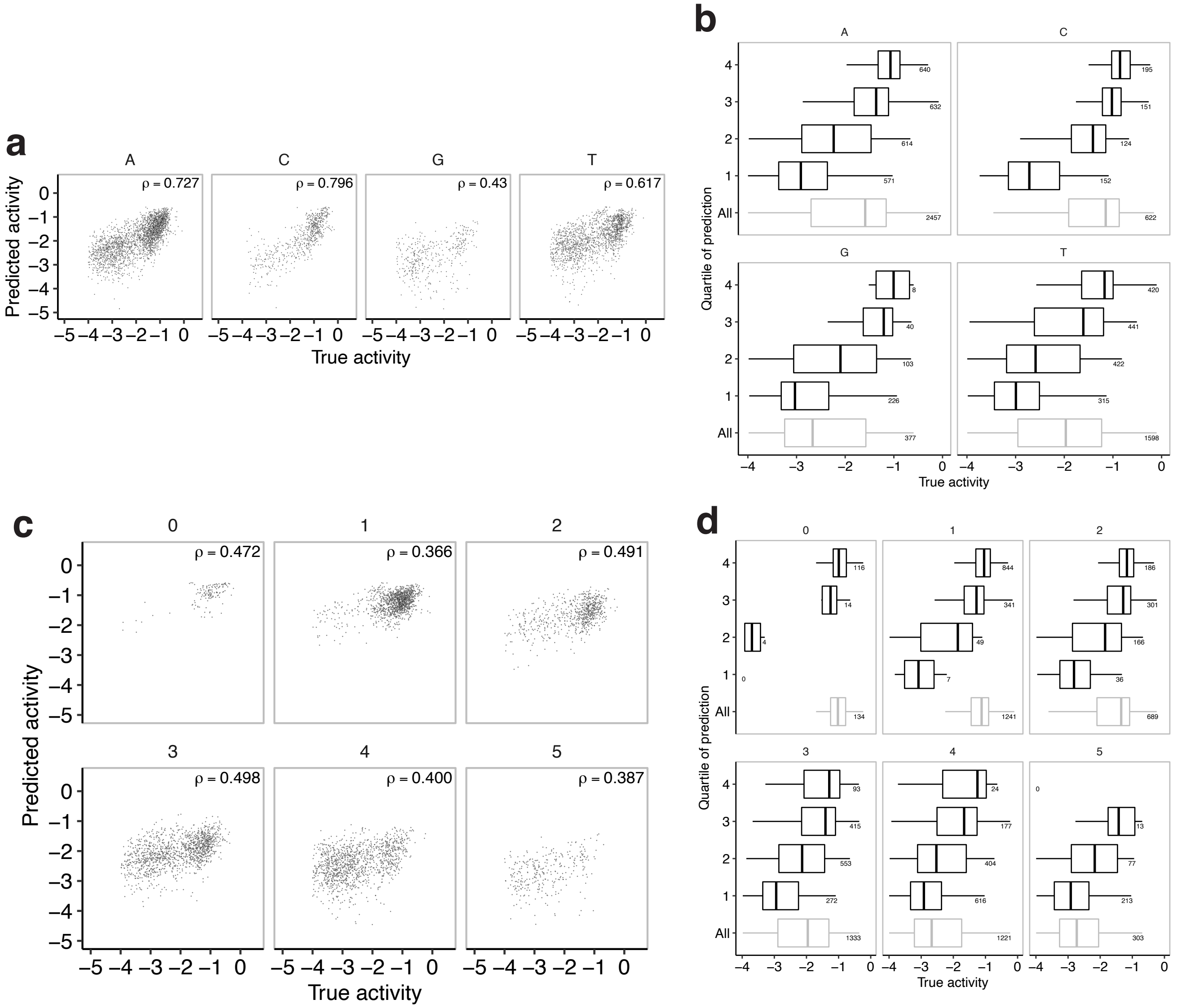

Supplementary Figure 10 — Regression performance on subsets of test data. Evaluations of regression on different subsets of active guide-target pairs in the hold-out test data, where the subsets correspond to features that considerably affect activity. Here, the model is the same for all evaluations and only tested (not trained) on different subsets. **a**, Pairs separated by the different protospacer flanking site (PFS) nucleotides, indicated above each plot. Each point is a guide-target pair. **b**, Pairs separated by the different PFS nucleotides. Each row contains one quartile based on their predicted activity (top row is predicted most active), with the bottom row showing all active pairs with the PFS. Adjacent numbers indicate the number of pairs (n) in each quartile; the quartile for each pair is based on its predicted activity across all PFS nucleotides, not only the PFS for each plot. **c**, Same as **a**, except separated by different Hamming distances between guide and target. **d**, Same as **b**, except separated by Hamming distance. In **a** and **c**, ρ is Spearman correlation. In **b** and **d**, boxes indicate first and third quartiles (25th and 75th percentiles), center bars indicate median, upper whiskers extend to maxima (if points are higher than 1.5 times the interquartile range from the box, then only up to that value with the remaining points treated as outliers and not shown), and lower whiskers likewise extend to minima.

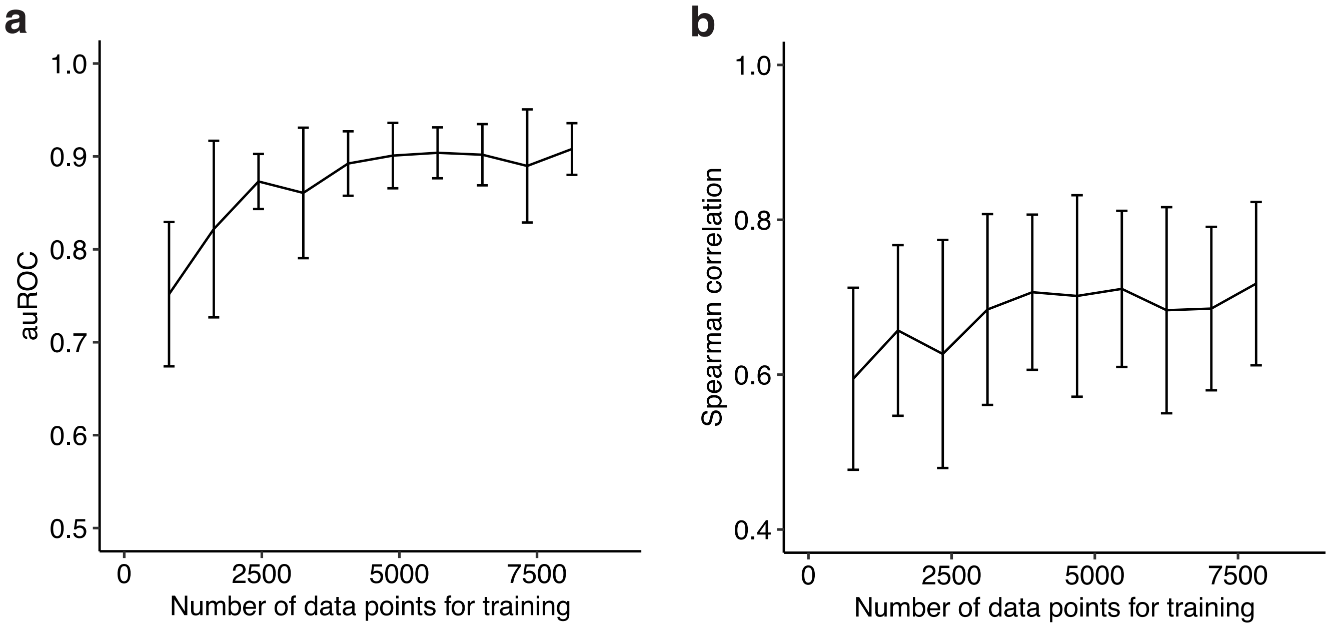

Supplementary Figure 11 — Learning curves. Learning curves for the convolutional neural networks used in ADAPT, which assess whether additional data could benefit model performance. At each number of input training data points, we perform nested cross-validation to select models: on each of five outer folds, we perform a five-fold cross-validated hyperparameter search to select a model. Line indicates the mean of a statistic on the validation data across the $n = 5$ selected models and error bars give a 95% confidence interval. **a**, Learning curve selecting models for classification. **b**, Learning curve selecting models for regression.

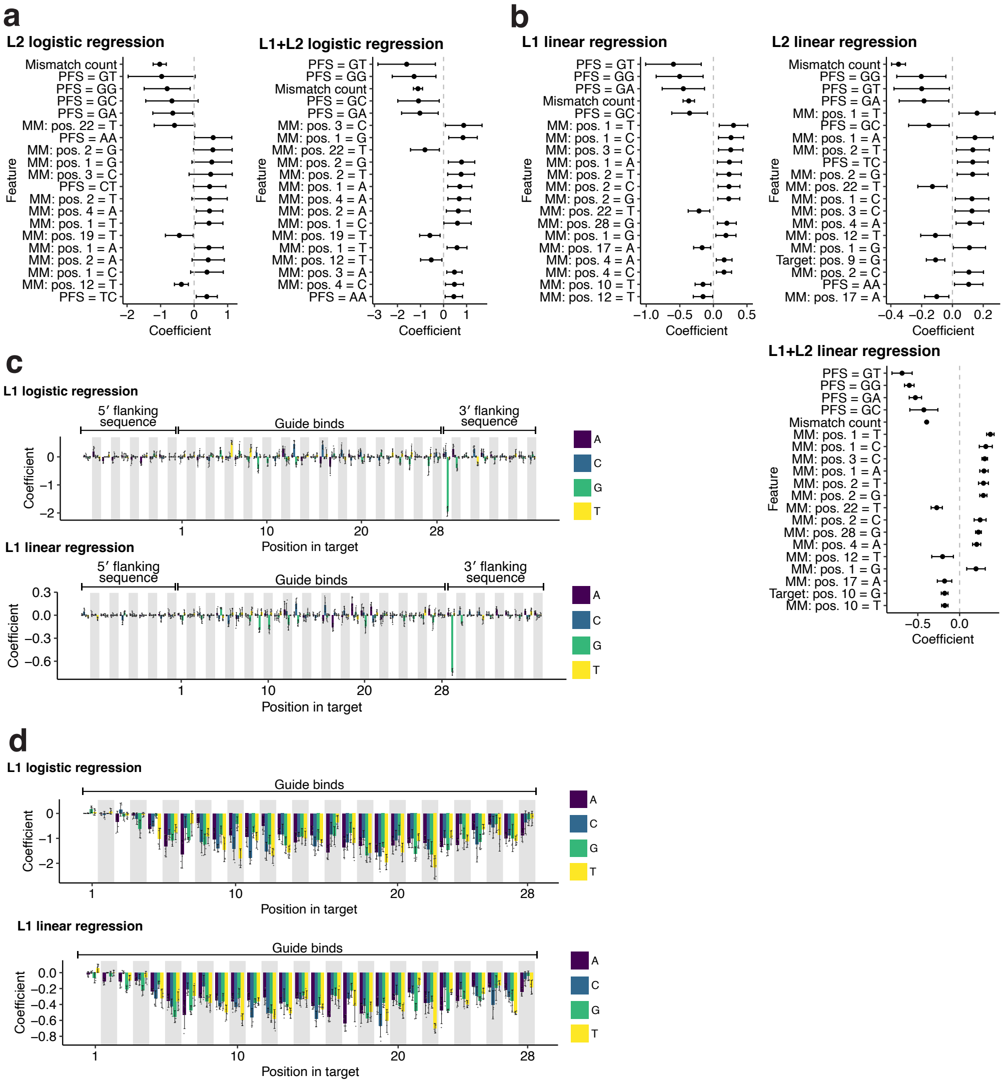

Supplementary Figure 12 — Importance of features in linear models. Feature coefficients in linear models for predicting guide activity. **a**, Linear models for classifying guide-target activity. Dot is the mean of the coefficient across training on $n = 5$ splits and error bar is the 95% confidence interval. Coefficients are ranked by absolute value and the top 20 are shown (PFS and number of mismatches dominate activity). Positions are along the target, where the guide binds at positions 1–28: position 1 is the 5′ end of the protospacer (position 28 of the guide spacer); position 28 is the 3′ end of the protospacer (position 1 of the guide spacer); positions −9–0 are 10-nt of context flanking the protospacer on the 5′ end; positions 29–38 are 10-nt of context flanking the protospacer on the 3′ end. Models use the 'One-hot MM + Handcrafted' input, which combines one-hot encoding of target sequence nucleotides and of mismatches in guides relative to the target with curated features of hypothesized importance (details in Methods). PFS, protospacer flanking site (flanking on 3′ side) including nucleotides at two positions. 'MM:' indicates a mismatch at the given position with the base representing the complement of the nucleotide in the guide's spacer. 'Target:' indicates a base in the target sequence, matching with the guide, at the given position. L1 logistic regression is in Fig. 2e. **b**, Same as **a** but for regression models on active guide-target pairs. **c**, Coefficients for nucleotide composition of the target—showing position-specific nucleotide preferences—from the L1 logistic regression model used for classifying activity and the L1 linear regression model used on active guide-target pairs. Bar is the mean of the coefficient across training on $n = 5$ splits and error bar is the 95% confidence interval. Models use the 'One-hot MM' input, which combines one-hot encoding of target sequence nucleotides and of mismatches in guides relative to the target (it leaves out the handcrafted features, including number of mismatches and two-nucleotide PFS interaction, present in **a** and **b**, so that they do not affect the coefficients along the target). Colors represent nucleotides in the target sequence. Outlier at position 29 indicates the effect of a `G` PFS. **d**, Same model and input as in **c**, but for the features representing guide-target mismatches along the target sequence in the region to which the spacer binds; features show the varying effect of mismatched nucleotides. Colors represent the complement of the nucleotide in the guide's spacer; for example, `A` indicates `T` in the spacer sequence that is mismatched with either `C`, `G`, or `T` in the target sequence.

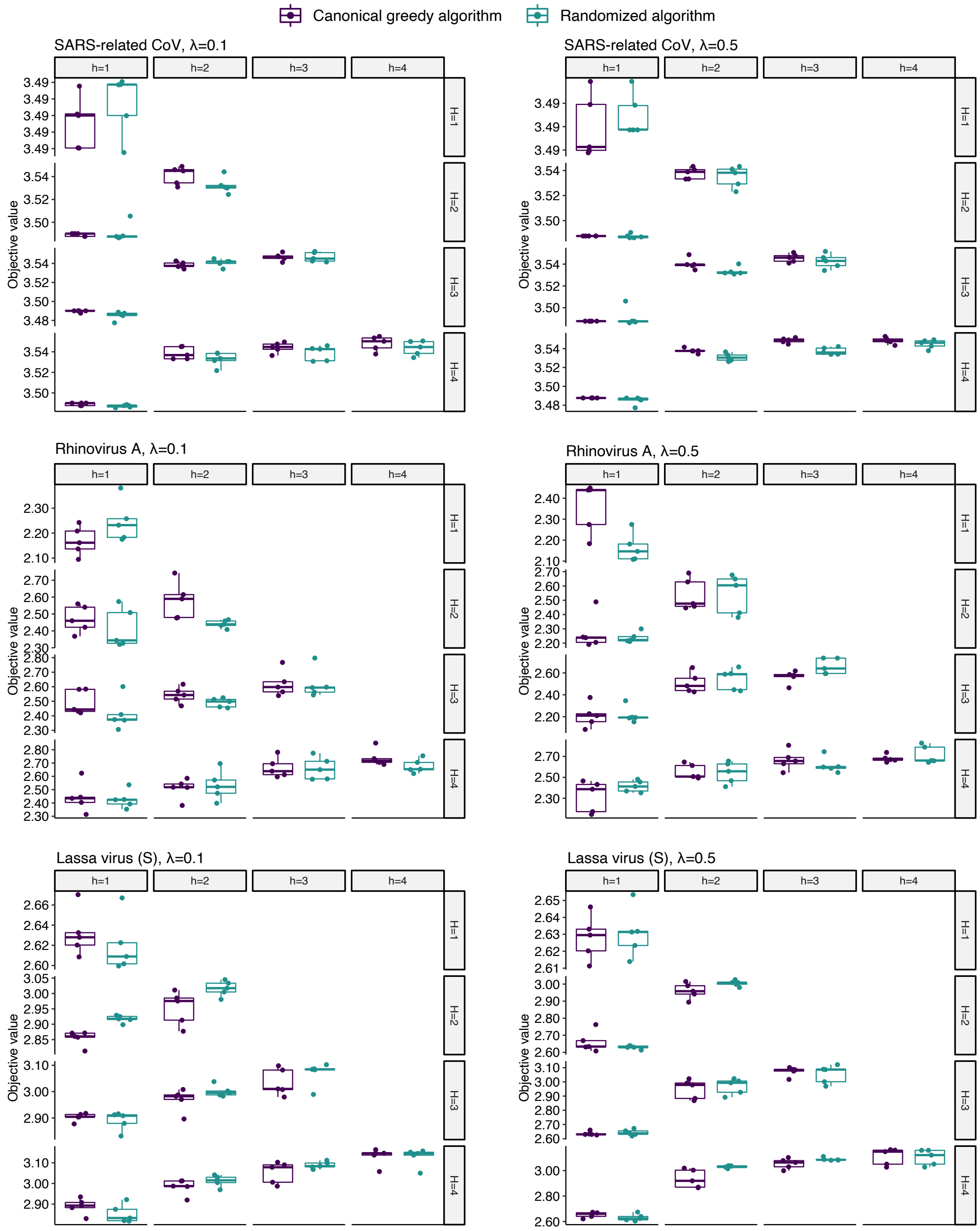

Supplementary Figure 13 — Comparison of algorithms for submodular maximization. Objective values of the optimal solutions identified by two algorithms for submodular maximization: the canonical greedy algorithm for monotone functions[6] and a randomized algorithm with provable guarantees on non-monotone functions[5]. The function here is non-monotone, but neither algorithm clearly outperforms the other. Three viral species are shown. Each was evaluated for two choices of the weight on the soft constraint/penalty, indicated by λ, as well as different choices of the soft cardinality constraint (h) and hard constraint (H) with $h \leq H$. Supplementary Note 2 contains a definition of the objective function, including the penalty weight and cardinality constraints. Each point indicates the result of one of $n = 5$ runs; differences account for randomness both in the randomized greedy algorithm and in constructing the ground set. Boxes indicate first and third quartiles (25th and 75th percentiles), center bars indicate median, upper whiskers extend to maxima (if points are higher than 1.5 times the interquartile range from the box, then only up to that value), and lower whiskers likewise extend to minima. Note that, for SARS-related CoV at $H = 1$, all values are the same up to numerical error.

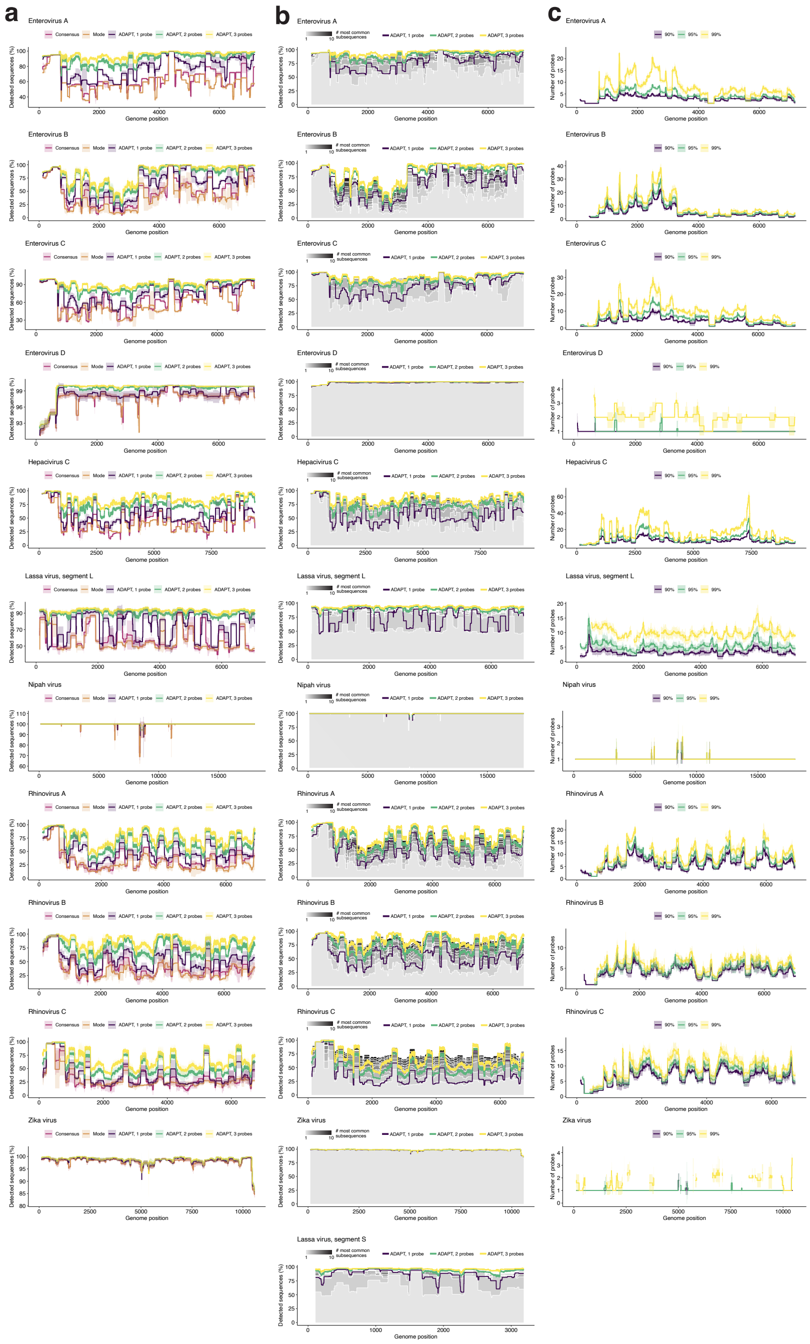

Supplementary Figure 14 — Comprehensiveness of probe design. Comparison of ADAPT's comprehensiveness in designing probe sequences with baseline methods, across 11 viral species. **a**, Fraction of genome sequences detected, with different design strategies in a 200 nt sliding window, using a model in which 30 nt probes detect a target if they are within 1 mismatch, counting G-U pairs at matches. Consensus, probe-length consensus subsequence from the window that detects the greatest number of genomes; Mode, most abundant probe-length subsequence within the window. Our approach (ADAPT) uses hard constraints of 1–3 probes and maximizes activity. **b**, Same as **a**, but generalizing the 'Mode' beyond one probe. Stacked grays show the cumulative fraction of genome sequences detected using the *n* probes representing the *n* most common subsequences at a site, ranging *n* from 1 (lightest gray) to 10 (darkest gray). Top of the lightest gray area corresponds to 'Mode' in **a** (1 probe) and top of all the grays is using 10 probes. **c**, Number of probes identified by ADAPT when solving a dual objective: minimizing the number of probes to detect >90%, >95%, and >99% of genome sequences using the model in **a**. Gaps at a site are present when it is not possible to construct a probe set that reaches the desired coverage, owing to gaps or missing data. In **a** and **c**, lines show the mean and shaded regions around them are 95% pointwise confidence bands across genomes sampled for each virus calculated by bootstrapping, i.e., randomly sampling genomes to be input to the design process; **b** shows only the mean, to ease visualization. Figure 3b,c show results from panel **a** and **c** for Lassa virus, segment S.

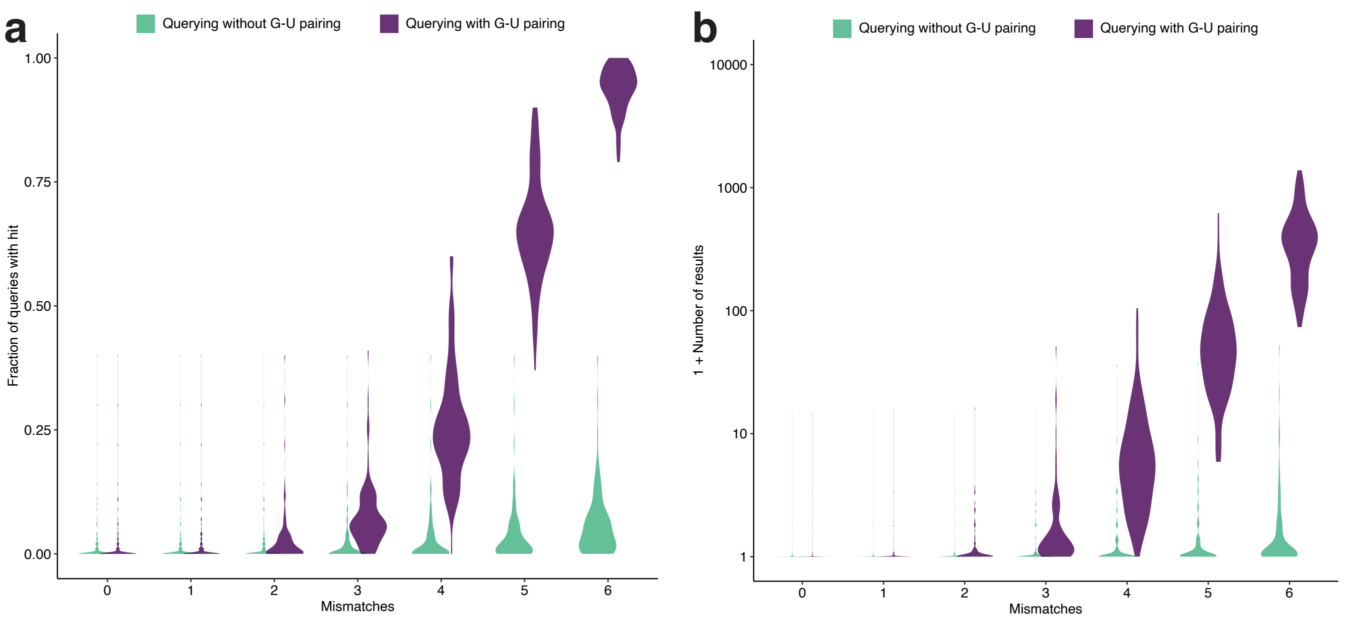

Supplementary Figure 15 — Potential hits with tolerance of G-U base pairing. Being tolerant of G-U base pairing increases the potential for non-specific hits of a *k*-mer. We built an index of $\sim$1 million 28-mers from 570 human-associated viral species. For each of 100 randomly selected species, we queried 28-mers for hits against the other 569 species (details in Methods). We performed this for each choice of m mismatches, counting a non-specific hit as one within m mismatches of the query, both being sensitive to G-U base pairing (purple; counting it as a match) and not being sensitive to it (green; counting it as a mismatch). Violin plots show the distribution, across the selected species, of the mean of the measured value taken over the queries for each species. **a**, Fraction of queries that yield a non-specific hit. The measured value for a query is 0 (no hit) or 1 ($\geq$ 1 hit), so the mean represents the fraction of queries with a hit. **b**, Number of non-specific hits per query.

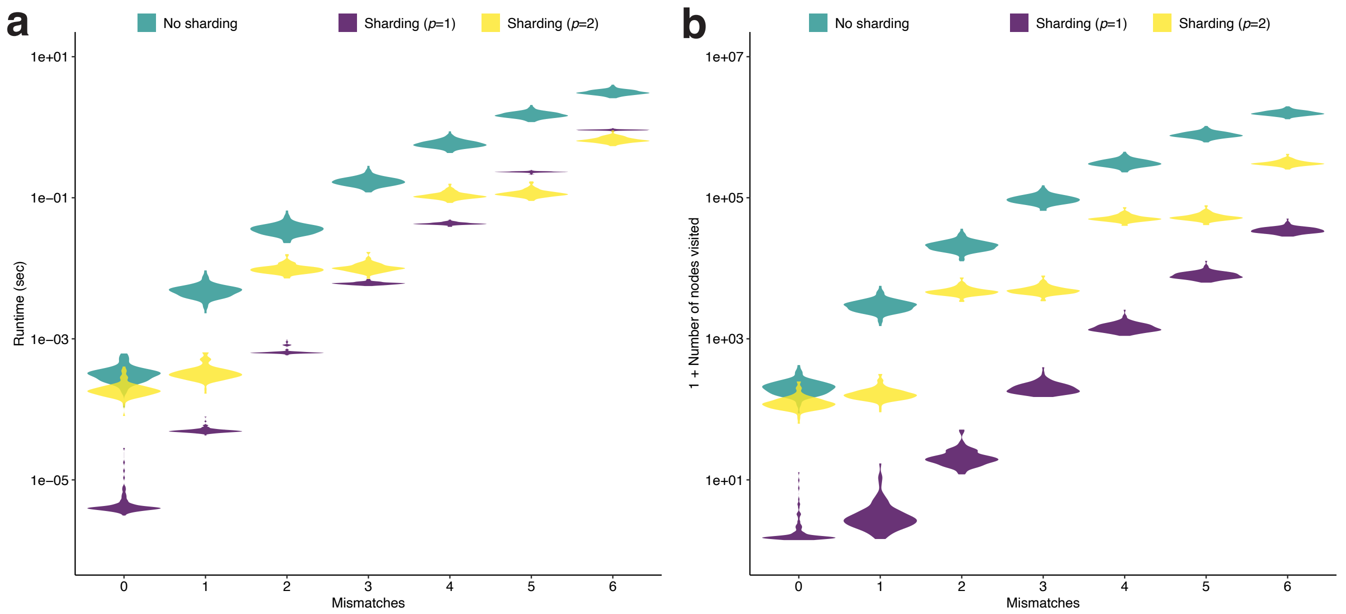

Supplementary Figure 16 — Benchmarking of specificity queries. **a**, The runtime of querying using an index of $\sim$1 million 28-mers across 570 human-associated viral species. For each of 100 randomly selected species, we queried 28-mers for hits against the other 569 species. Violin plots show the distribution, across the selected species, of the mean runtime for each query. Green shows results on a single, large trie of 28-mers; purple ($p = 1$) and yellow ($p = 2$) show results on the approach described in Supplementary Note 3d, with two choices of the partition number p. **b**, Same as **a**, but showing total number of nodes visited across the trie(s). The decrease in this value using our approach suggests that parallelizing the approach—by searching within multiple tries in parallel—may provide a further speedup.

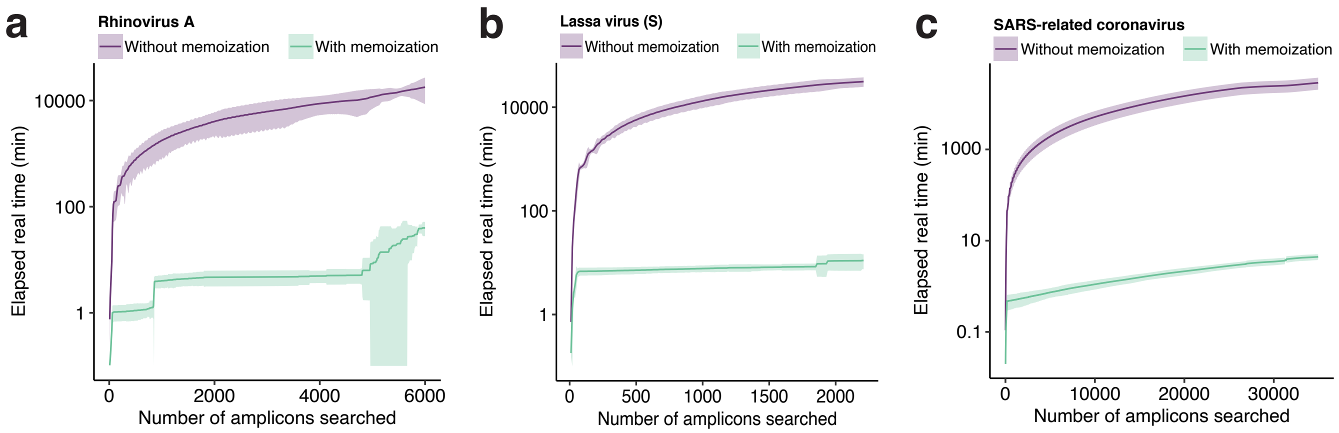

Supplementary Figure 17 — Runtime improvement provided by memoization. Runtime of ADAPT's search with and without memoizing computations, for three species. We plot the cumulative elapsed real time (minutes) at each successive window (amplicon) that ADAPT considers during its search. Shaded regions indicate a 95% confidence interval calculated across 3 runs (same input) and line is the mean. **a**, Rhinovirus A. The lower end of the confidence interval is cutoff at 0.1. Memoization provides a 99.71% reduction in runtime (mean). **b**, Lassa virus, segment S. Memoization provides a 99.75% reduction in runtime (mean). **c**, SARS-related coronavirus. Memoization provides a 99.96% reduction in runtime (mean). For **b** and **c**, the search without memoization was ended before its completion; thus, for these, the reduction is a lower bound assuming a faster growth of the runtime without memoization.

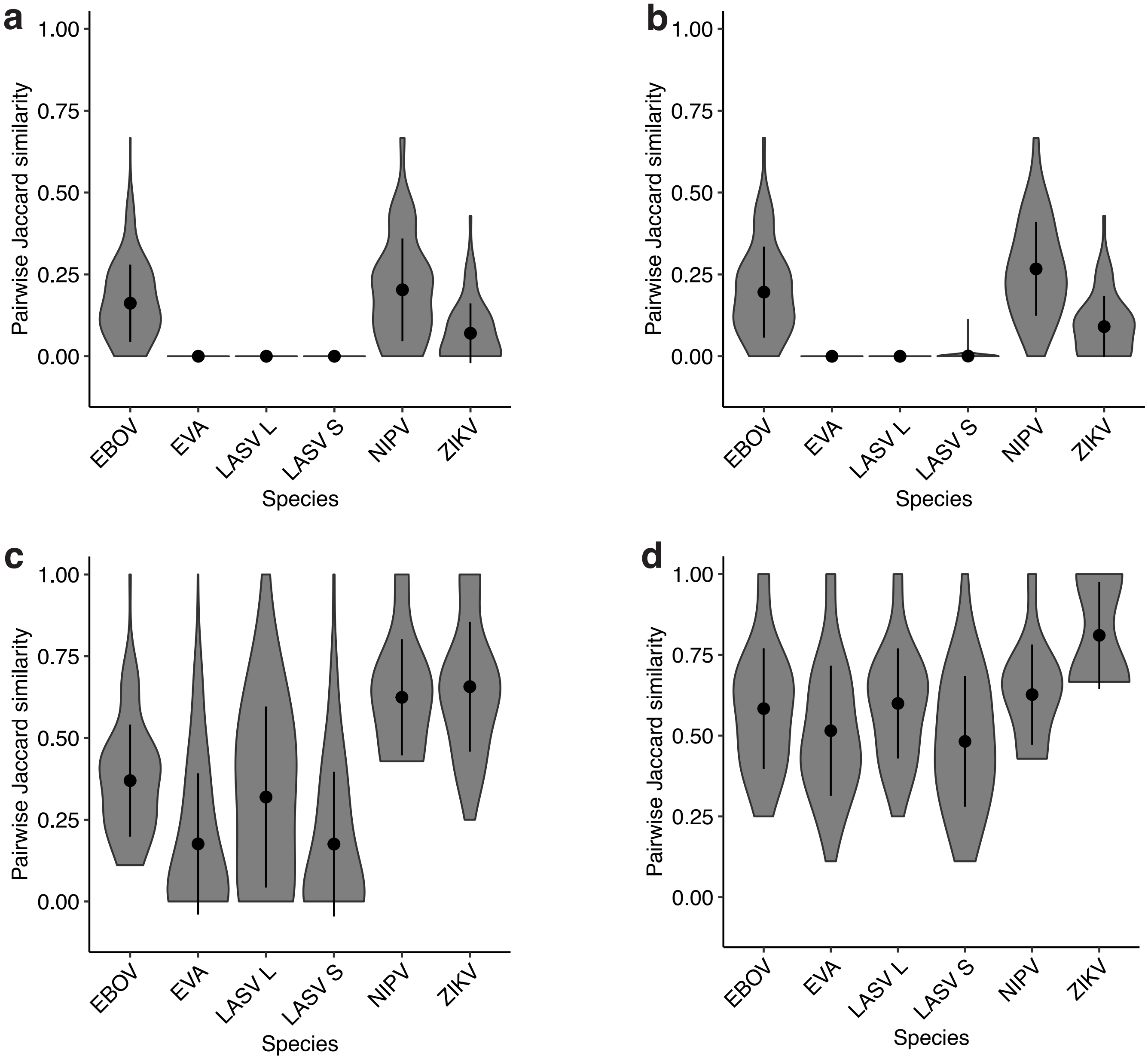

Supplementary Figure 18 — Dispersion in ADAPT's designs. For each species, we ran ADAPT 20 times. For each pair of runs, we calculated the Jaccard similarity comparing the top 5 design options from each. Violin plots show a smoothed density estimate of the pairwise Jaccard similarities. Dot indicates the mean and bars show 1 standard deviation around the mean across the $n = 190$ pairs for each species ($n = 171$ for LASV S because one of the 20 runs produced only 4 design options rather than 5, thereby providing 19 runs to compare). Lower values indicate more variability in ADAPT's design outputs across runs. The panels show different methods of providing input genomes and of comparing a pair of design outputs. **a**, Using resampled input genomes for each run and considering two design options to be equal if they have exactly the same primers and probes. **b**, Using the same input genomes for each run and considering two design options to be equal if they have exactly the same primers and probes. **c**, Using resampled input genomes for each run and considering two design options to be equal if their endpoints are within 40 nt of each other. **d**, Using the same input genomes for each run and considering two design options to be equal if their endpoints are within 40 nt of each other. When using resampled input genomes, the comparisons account for algorithmic randomness and input sampling. When using the same input genomes, the comparisons account only for algorithmic randomness. EBOV, Zaire ebolavirus; EVA, Enterovirus A; LASV L/S, Lassa virus segment L/S; NIPV, Nipah virus; ZIKV, Zika virus.

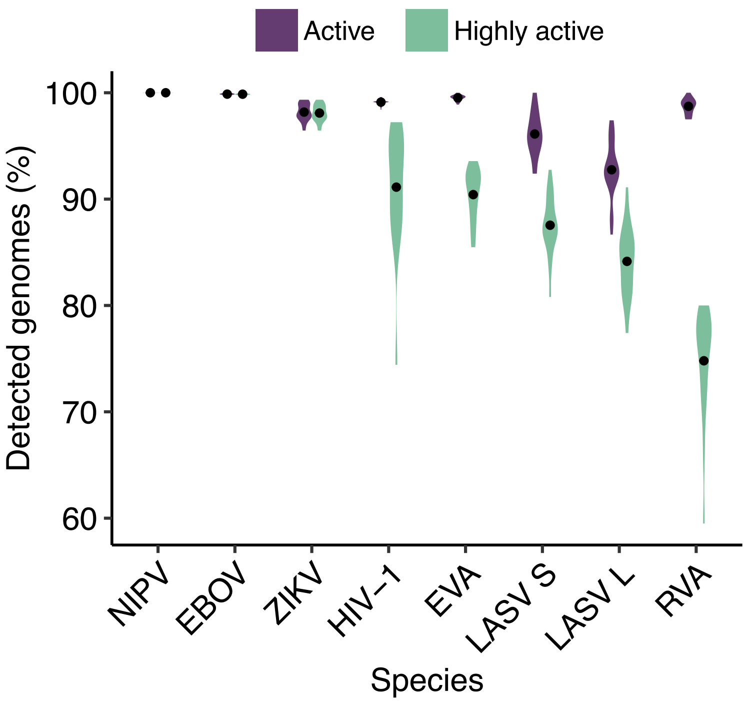

Supplementary Figure 19 — Cross-validated evaluation of detection with relaxed design parameters. Cross-validated evaluation of detection using more relaxed design parameters than the choices in Fig. 4b; the relaxed parameters (Methods) tolerate more complex assay designs (e.g., more guides) to achieve higher sensitivity. For each species, we ran ADAPT on 80% of available genomes and estimated performance, averaged over the top 5 design options, on the remaining 20%. Distributions are across 20 random splits and dots indicate mean. Purple, fraction of genomes detected by primers and for which Cas13a guides are classified as active. Green, same except Cas13a guides also have regressed activity in the top 25% of our dataset. NIPV, Nipah virus; EBOV, Zaire ebolavirus; ZIKV, Zika virus; LASV S/L, Lassa virus segment S/L; EVA, Enterovirus A; RVA, Rhinovirus A.

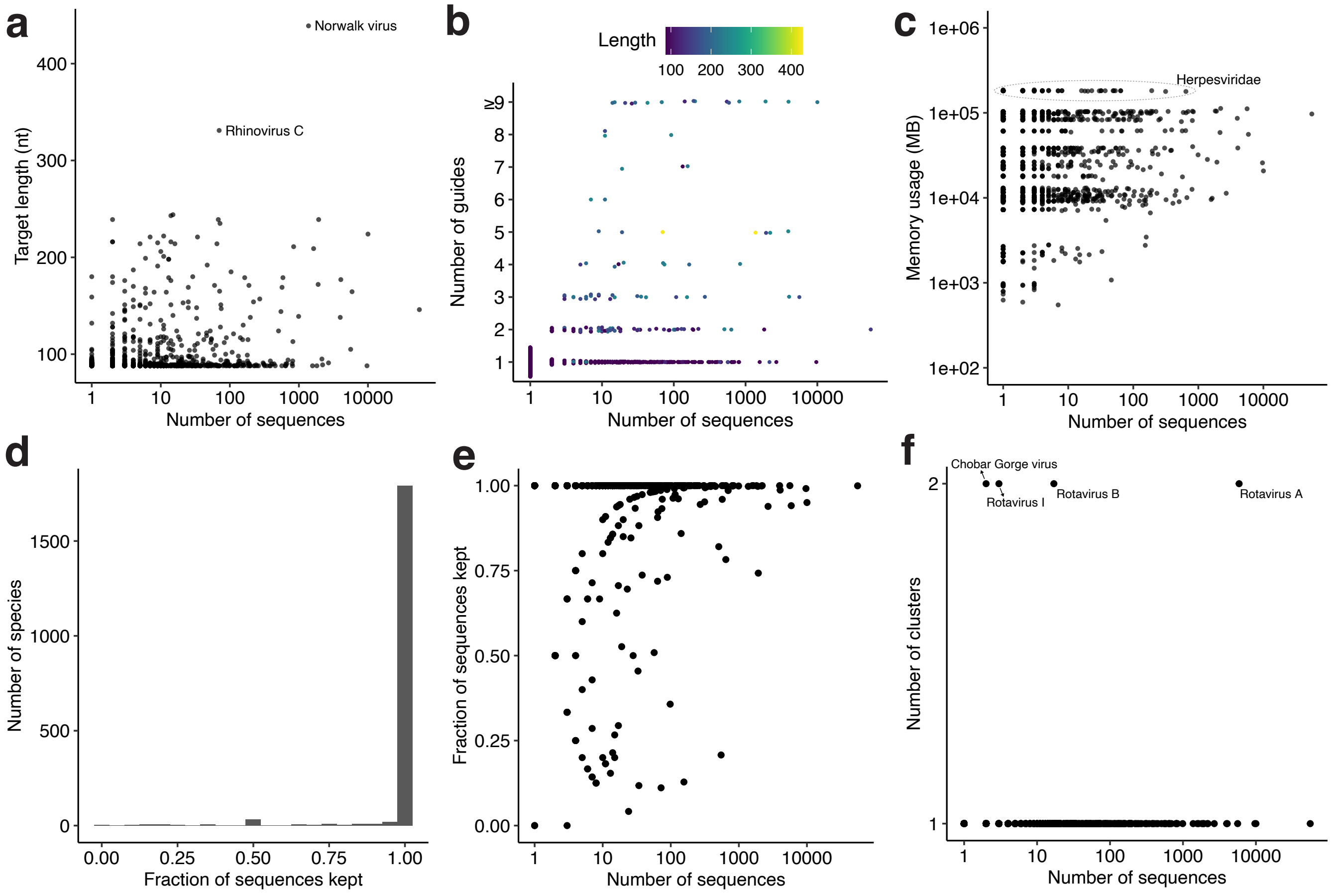

Supplementary Figure 20 — Results of ADAPT's designs for 1,926 vertebrate-associated viruses. Running ADAPT on 1,933 vertebrate-associated viral species produced designs on 1,926 (Methods). **a**, Length of each target region, i.e., amplicon, in nt of the highest-ranked design output by ADAPT for each species. As part of the design we restricted the length to $\leq$ 250-nt for all species except two (Methods). Horizontal axis is the number of input sequences for design. **b**, Number of Cas13a guides in the highest-ranked design option for each species, produced using an objective function in which we minimize the number of guides subject to detecting $>$ 98% of sequences with high activity. This objective function is a reformulation of, and differs from, our primary objective of maximizing activity. Color indicates the length of the targeted region (amplicon) in the design. 40 species have more than 3 guides; the most is 73 (Enterovirus B). **c**, Maximum resident set size (RSS), in MB, of the process running ADAPT on each species. Here, as in **a**, ADAPT is run using our objective function that maximizes activity. **d**, Distribution, across species, of the fraction of input sequences passing curation. **e**, Fraction of input sequences passing curation for each species compared the number of input sequences for that species. **f**, Number of clusters for each species compared to the number of input sequences for that species. In **a**–**c** and **e**–**f**, each point is a species.

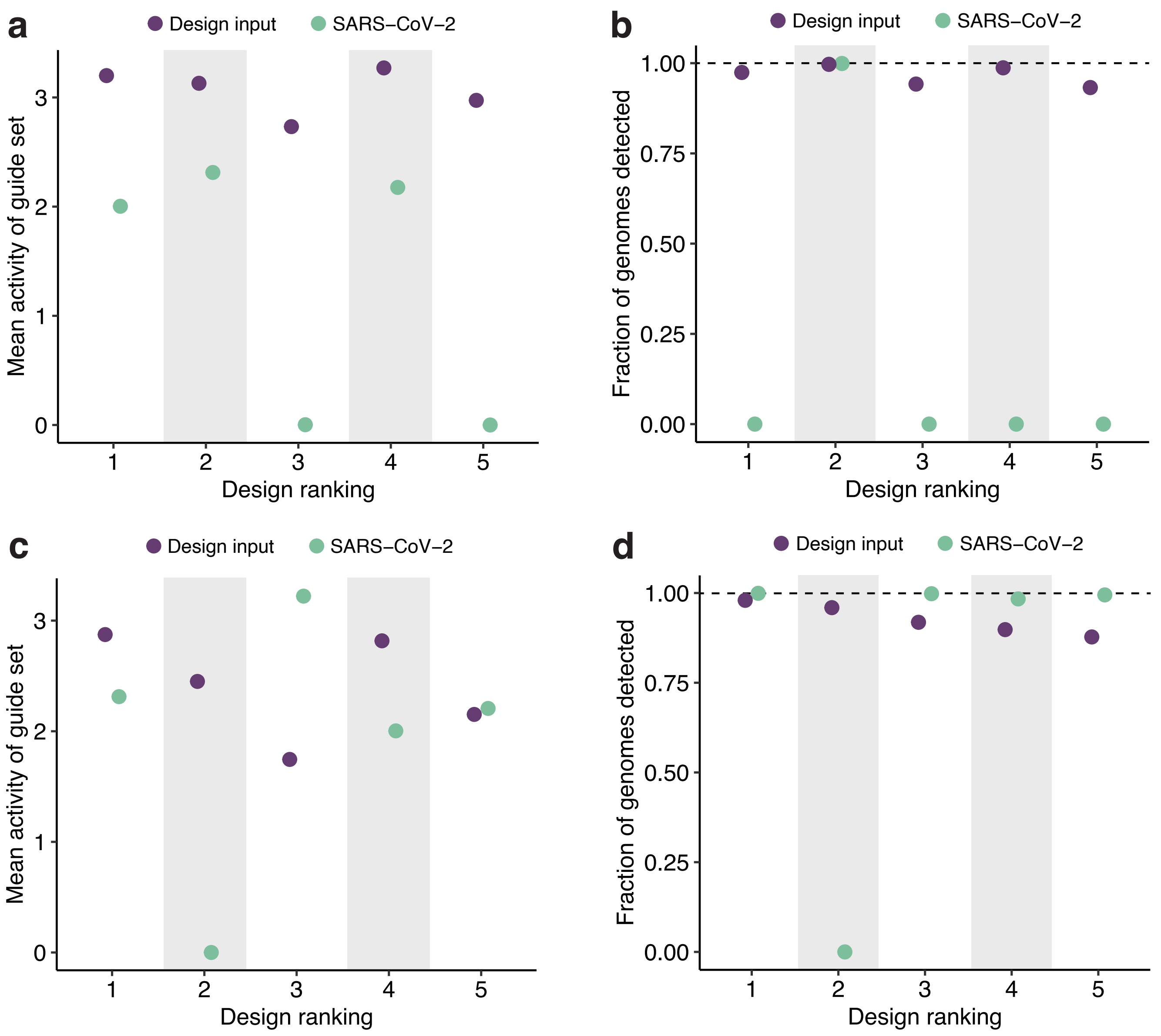

Supplementary Figure 21 — Evaluation of SARS-CoV-2 detection with assays designed, using genomes through 2018, to detect SARS-related coronavirus. The SARS-related CoV designs were generated using genomes available through the end of 2018, which simulates the design of broadly-effective assays a year before SARS-CoV-2's emergence. SARS-related CoV is a species that encompasses SARS-CoV-2, as well as SARS-CoV-1 and viruses sampled from wildlife. **a**, Performance of Cas13a guides from each of the five highest-ranked design outputs from ADAPT (ordered by ranking; 1 is best). Points indicate the mean predicted activity of each design's guides in detecting targeted genomes. Purple, mean across the 311 genomes used for the design (all SARS-related CoV genomes through the end of 2018). Green, mean across the 184,197 SARS-CoV-2 genomes available through November 12, 2020. **b**, Fraction of genomes predicted to be detected by each design's assay, accounting for both the primers and guides in the assay (details in Methods). Designs were produced as in **a** and colors are as in **a**. **c**, Same as **a**, except the designs used input that downsampled SARS-CoV-1 to a single genome, effectively down-weighing consideration to SARS-CoV-1 in the design. Purple, mean across the 49 genomes used for the design. Green, mean across the 184,197 SARS-CoV-2 genomes available through November 12, 2020. **d**, Fraction of genomes predicted to be detected by each design's assay, accounting for both the primers and guides in the assay. Designs were produced as in **c** and colors are as in **c**. In **a** and **c**, values at 0 indicate Cas13a guides that are classified as inactive; values above 0 are classified as active.

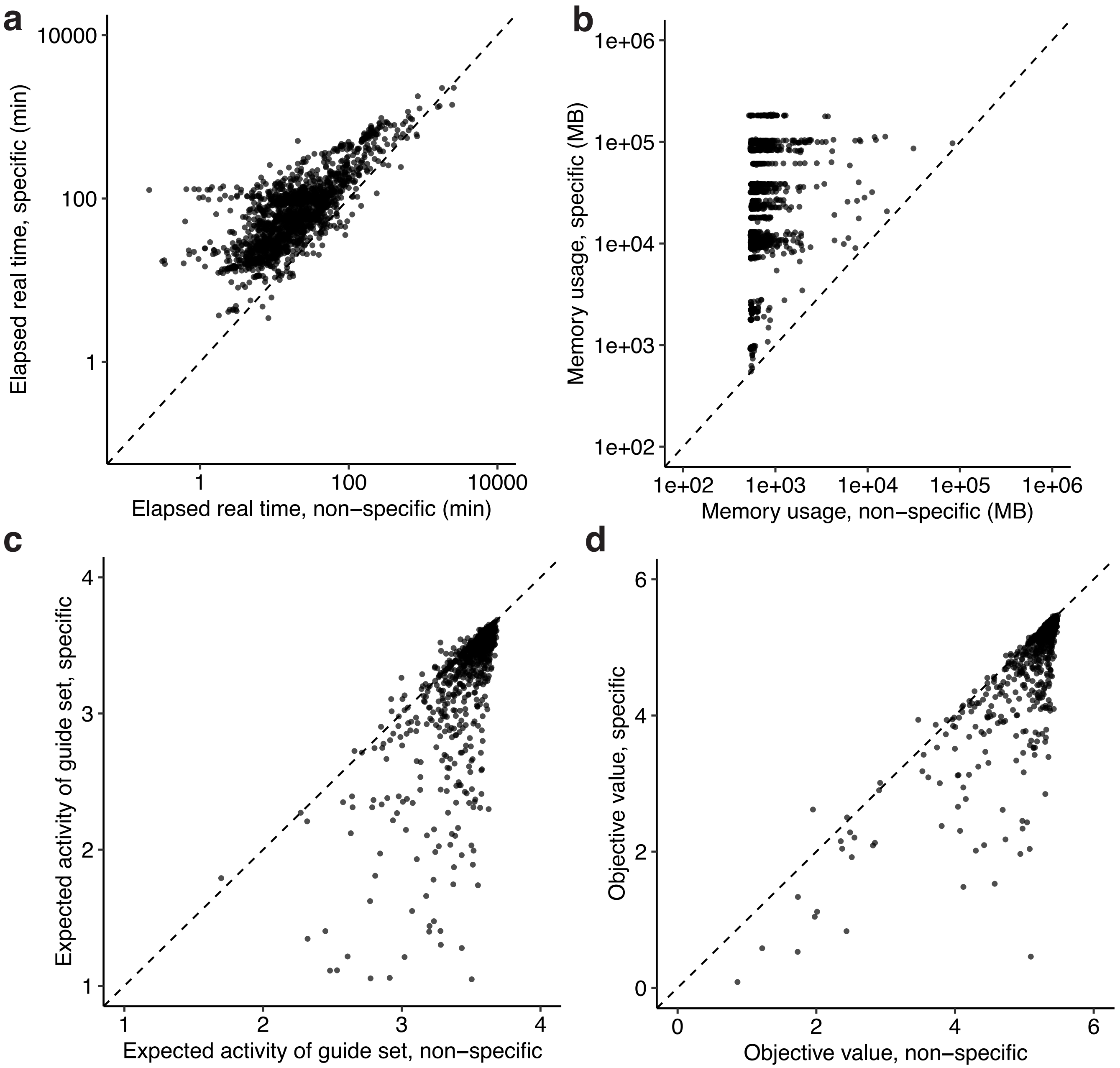

Supplementary Figure 22 — Effects of enforcing specificity on ADAPT's designs for 1,926 vertebrate-associated viruses. In each panel, each point is a species and comparisons are with and without enforcing species-level specificity within each family. **a**, End-to-end elapsed real time running ADAPT. **b**, Maximum resident set size (RSS), in MB, of the process running ADAPT. **c**, Mean activity of the guide set, from the highest-ranked design option, across input sequences. **d**, Objective value of the highest-ranked design option, which incorporates expected activity of the guide set, the number of primers, and the target region length. Not shown, 9 species with objective value < 0. In all panels, 1,926 species are shown (7 of the 1,933 vertebrate-associated species did not produce designs; Methods).

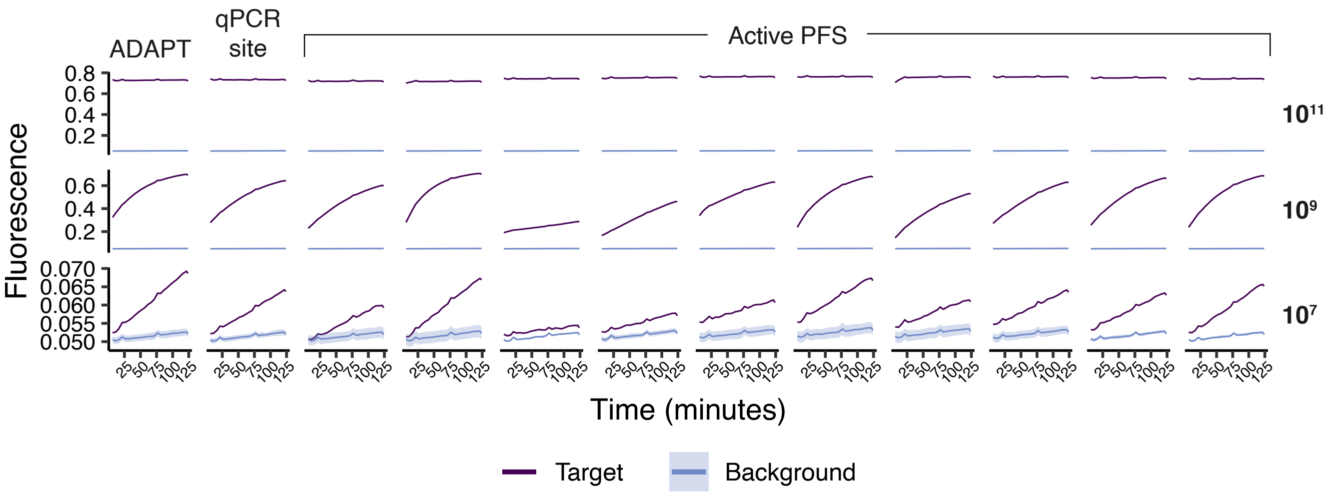

Supplementary Figure 23 — No-template background control fluorescence. Fluorescence over time against the no-template background control (water; blue) and against the template (purple) for each guide tested in the US CDC's SARS-CoV-2 N1 RT-qPCR amplicon. Guides, separated by columns, are: ADAPT's design, a guide with an active (non-`G`) PFS at the site of the qPCR probe, and 10 randomly selected guides with an active PFS. Labels on the right indicate target concentration in cp/μL (irrelevant for the background values). Shaded regions around the background values are 95% pointwise confidence bands across $n = 7$ replicates. Unlike in other plots of fluorescence, here the plotted values are not background-subtracted.

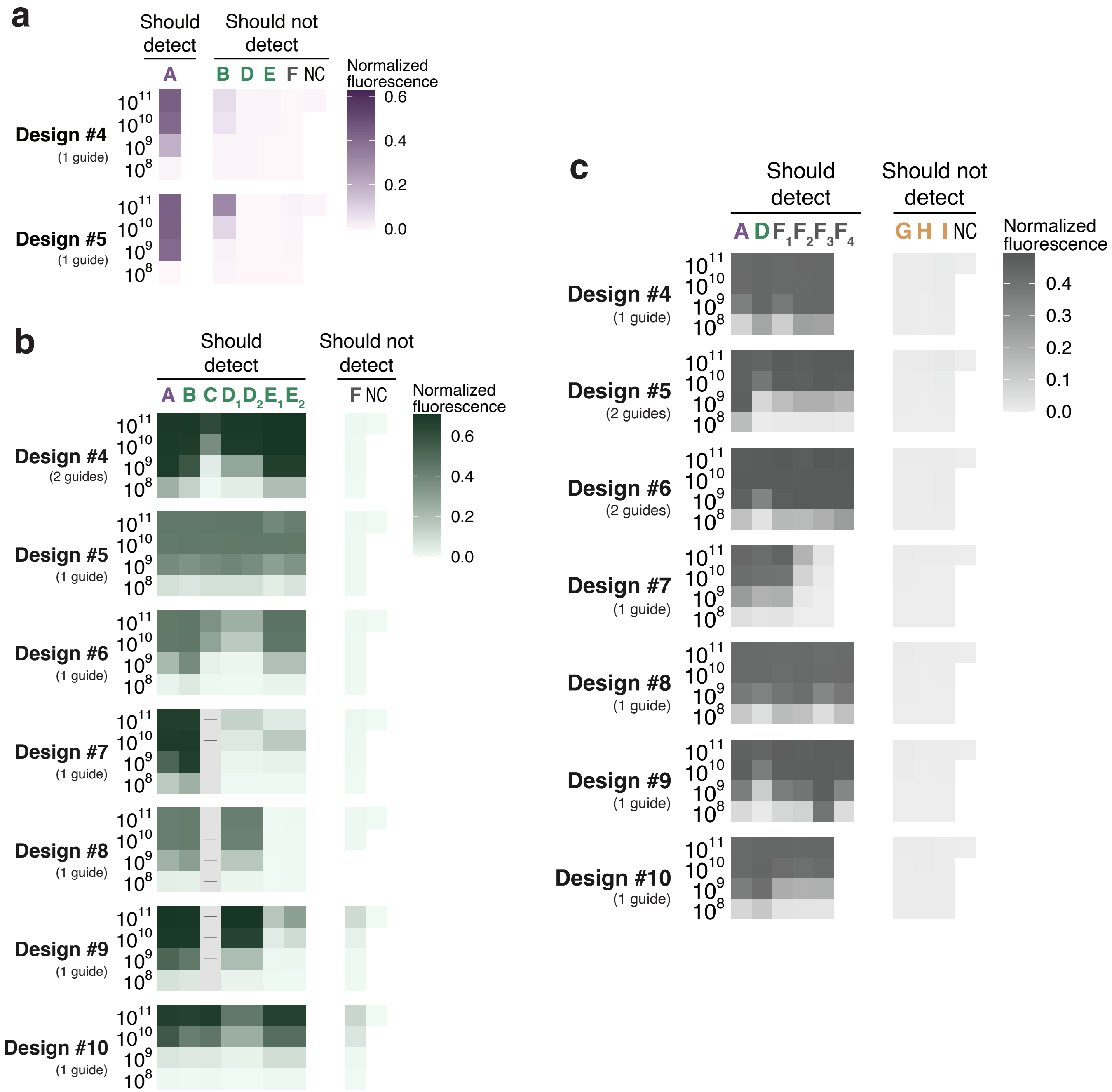

Supplementary Figure 24 — Sensitivity and specificity of additional designs for SARS-related CoV taxa. Fluorescence for ADAPT's designs specific to **a**, SARS-CoV-2, **b**, SARS-CoV-2–related, and **c**, SARS-related coronavirus species. Fig. 5c shows phylogenetic relationships of these taxa. Assays are ranked by ADAPT's predicted performance. Assays ranked from 4 through 10 are shown (only 5 tested for SARS-CoV-2); the top 3 are shown in Fig. 5. Target definitions are in Fig. 5c and the Fig. 5 legend provides additional details about each panel. In **c**, clade F required a fourth representative target (F_4) in only some amplicons. In all panels, parenthetical numbers are the number of Cas13 guides in ADAPT's design. NC, no template control.

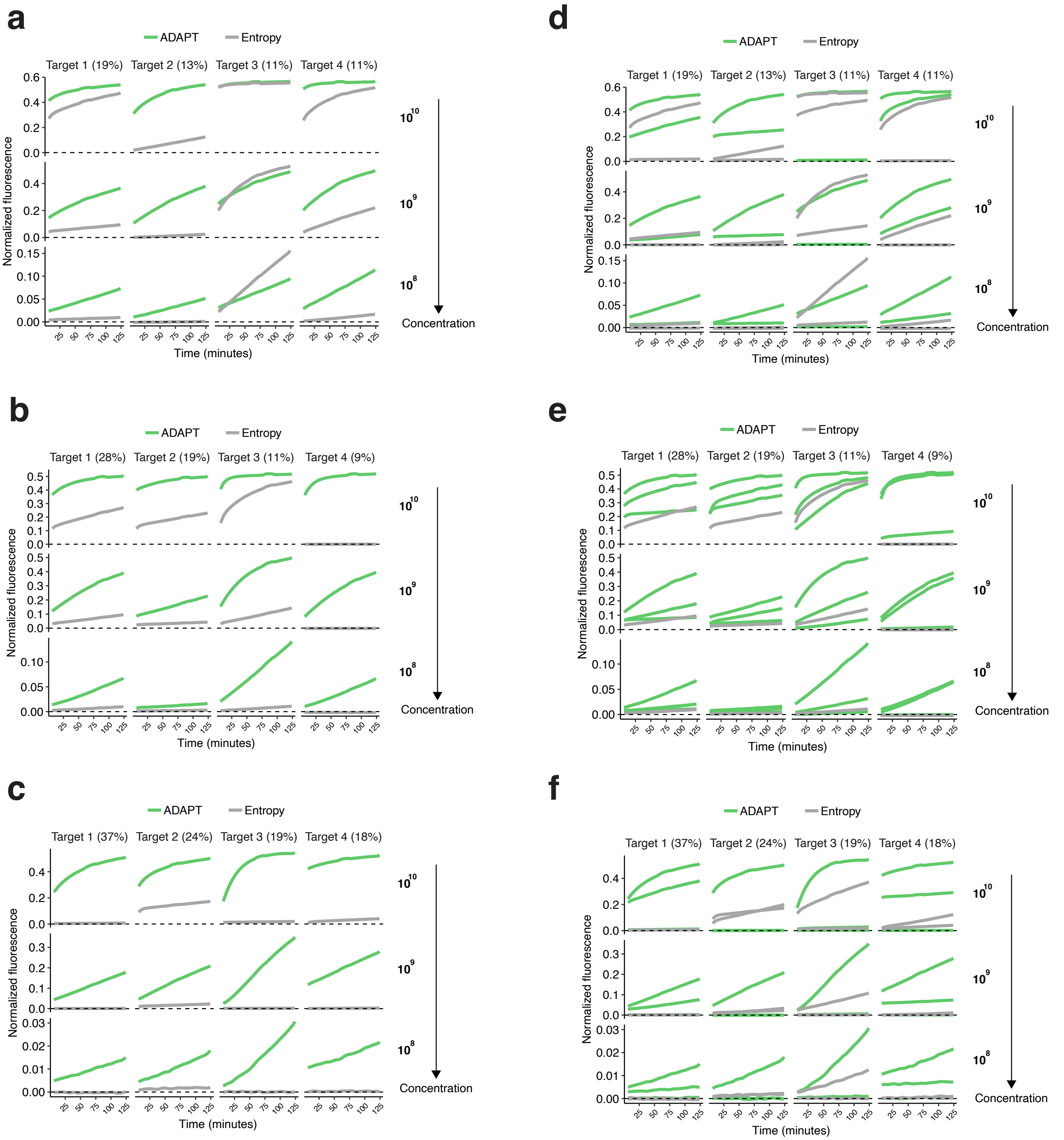

Supplementary Figure 25 — Kinetic curves of designs for detecting Enterovirus B. a–c, Fluorescence over time for ADAPT's designs in detecting EVB at varying target concentrations (right of each plot in cp/μL), for the 4 targets representing the largest fraction of EVB genomic diversity within the corresponding amplicon. **a**, Design #1 (highest ranked output design by predicted performance); **b**, Design #2; **c**, Design #3. Plots at the target concentration of 10^8 cp/μL are also shown in Fig. 5h. The Entropy guide (gray) targets the site from ADAPT's amplicon with an active PFS and minimal Shannon entropy. **d–f**, Same as **a–c** except with a separate line for each guide, when there are multiple guides in ADAPT's design or 2 guides tested for the entropy-based approach. **d**, Design #1; **e**, Design #2; **f**, Design #3. When there are two Entropy guides, they have an active PFS and the least and second-least Shannon entropy in the amplicon of ADAPT's design.

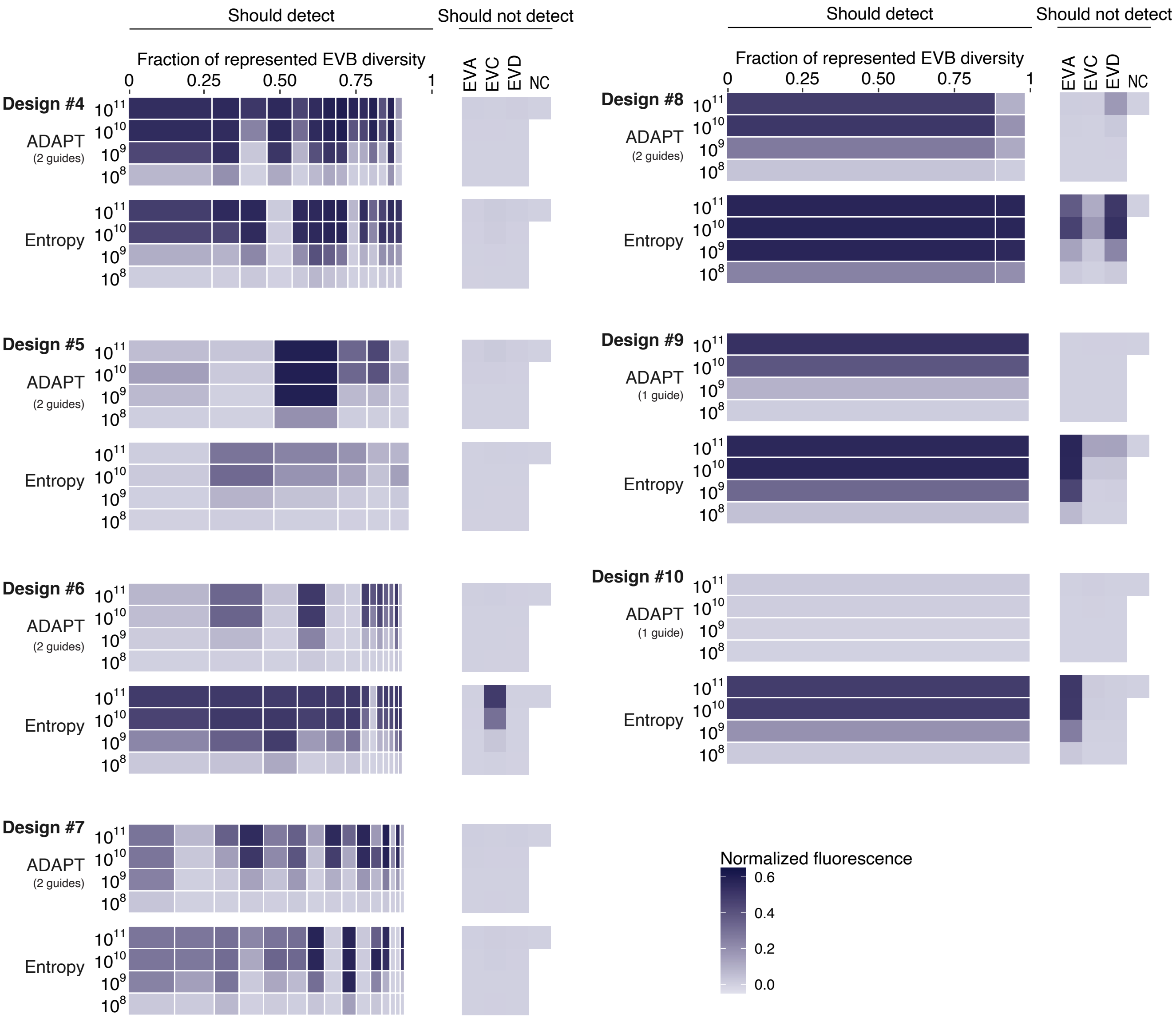

Supplementary Figure 26 — Sensitivity and specificity of additional designs for Enterovirus B. Fluorescence for ADAPT's Enterovirus B (EVB) designs in detecting EVB and representative targets for Enterovirus A/C/D (EVA/C/D). Assays ranked from 4 through 10 are shown; the top 3 are shown in Fig. 5g. Each band is an EVB target having width proportional to the fraction of EVB genomic diversity represented by the target, within the amplicon of ADAPT's design. Immediately under each ADAPT design is one baseline guide ("Entropy") from the site in the amplicon with an active PFS and minimal Shannon entropy. Values immediately to the left of the bands indicate target concentration (cp/μL), and parenthetical numbers are the number of Cas13 guides in ADAPT's design. NC, no template control.

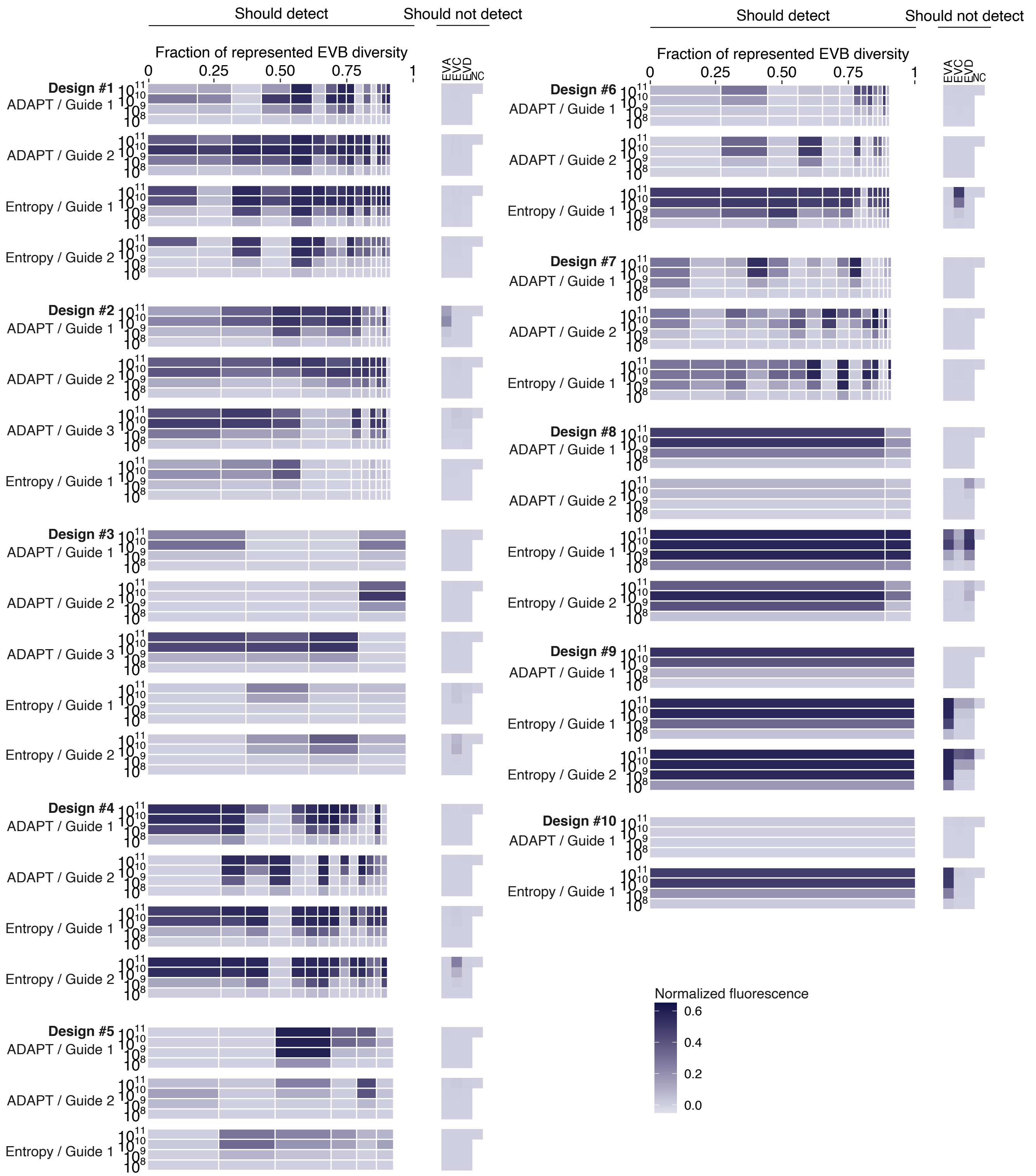

Supplementary Figure 27 — Separate guides in designs for detecting Enterovirus B. Fluorescence for ADAPT's Enterovirus B (EVB) designs in detecting EVB and representative targets for Enterovirus A/C/D (EVA/C/D), separated by guide. Each band is an EVB target having width proportional to the fraction of EVB genomic diversity represented by the target, within the amplicon of ADAPT's design. Values immediately to the left of the bands indicate target concentration (cp/μL). Immediately under each ADAPT design is one baseline guide ("Entropy") from the site in the amplicon with an active PFS and minimal Shannon entropy; when there are two Entropy guides, the second is from the site with an active PFS and the second-least entropy. In Fig. 5g and Supplementary Fig. 26, plotted value for ADAPT is the maximum across multiple guides. NC, no template control.

References

[1] Tambe, A., East-Seletsky, A., Knott, G. J., Doudna, J. A. & O'Connell, M. R. RNA binding and HEPN-Nuclease activation are decoupled in CRISPR-Cas13a. *Cell Reports* **24**, 1025–1036 (2018).

[2] East-Seletsky, A., O'Connell, M. R., Burstein, D., Knott, G. J. & Doudna, J. A. RNA targeting by functionally orthogonal type VI-A CRISPR-Cas enzymes. *Molecular Cell* **66**, 373–383.e3 (2017).

[3] Abudayyeh, O. O. *et al.* RNA targeting with CRISPR-Cas13. *Nature* **550**, 280–284 (2017).

[4] Linhart, C. & Shamir, R. The degenerate primer design problem. *Bioinformatics* **18 Suppl 1**, S172–81 (2002).

[5] Buchbinder, N., Feldman, M., Naor, J. s. & Schwartz, R. Submodular maximization with cardinality constraints. In *Proceedings of the twenty-fifth annual ACM-SIAM symposium on Discrete algorithms*, SODA '14, 1433–1452 (Society for Industrial and Applied Mathematics, USA, 2014).

[6] Nemhauser, G. L., Wolsey, L. A. & Fisher, M. L. An analysis of approximations for maximizing submodular set functions. *Mathematical Programming. A Publication of the Mathematical Programming Society* **14**, 265–294 (1978).

[7] Chvatal, V. A greedy heuristic for the Set-Covering problem. *Mathematics of Operations Research* **4**, 233–235 (1979).

[8] Johnson, D. S. Approximation algorithms for combinatorial problems. *Journal of Computer and System Sciences* **9**, 256–278 (1974).

[9] Pearson, W. R., Robins, G., Wrege, D. E. & Zhang, T. On the primer selection problem in polymerase chain reaction experiments. *Discrete Applied Mathematics* **71**, 231–246 (1996).

[10] Jabado, O. J. *et al.* Greene SCPrimer: a rapid comprehensive tool for designing degenerate primers from multiple sequence alignments. *Nucleic Acids Research* **34**, 6605–6611 (2006).

[11] Huang, Y.-C. *et al.* Integrated minimum-set primers and unique probe design algorithms for differential detection on symptom-related pathogens. *Bioinformatics* **21**, 4330–4337 (2005).

[12] Duitama, J. *et al.* PrimerHunter: a primer design tool for PCR-based virus subtype identification. *Nucleic Acids Research* **37**, 2483–2492 (2009).

[13] Kreer, C. *et al.* openPrimeR for multiplex amplification of highly diverse templates. *Journal of Immunological Methods* **480**, 112752 (2020).

[14] Feige, U. A threshold of ln n for approximating set cover. *Journal of the ACM* **45**, 634–652 (1998).

[15] Moshkovitz, D. The projection games conjecture and the NP-Hardness of ln n-Approximating Set-Cover. *Theory of Computing* **11**, 221–235 (2015).

[16] Har-Peled, S. & Jones, M. Few cuts meet many point sets. *arXiv* (2018). 1808.03260.

[17] Varani, G. & McClain, W. H. The G x U wobble base pair. a fundamental building block of RNA structure crucial to RNA function in diverse biological systems. *EMBO Reports* **1**, 18–23 (2000).

[18] Saxena, S., Jónsson, Z. O. & Dutta, A. Small RNAs with imperfect match to endogenous mRNA repress translation. implications for off-target activity of small inhibitory RNA in mammalian cells. *The Journal of Biological Chemistry* **278**, 44312–44319 (2003).

[19] Du, Q., Thonberg, H., Wang, J., Wahlestedt, C. & Liang, Z. A systematic analysis of the silencing effects of an active siRNA at all single-nucleotide mismatched target sites. *Nucleic Acids Research* **33**, 1671–1677 (2005).

[20] Snøve, O., Jr & Holen, T. Many commonly used siRNAs risk off-target activity. *Biochemical and biophysical research communications* **319**, 256–263 (2004).

[21] Naito, Y., Yamada, T., Ui-Tei, K., Morishita, S. & Saigo, K. sidirect: highly effective, target-specific siRNA design software for mammalian RNA interference. *Nucleic Acids Research* **32**, W124–9 (2004).

[22] Qiu, S., Adema, C. M. & Lane, T. A computational study of off-target effects of RNA interference. *Nucleic Acids Research* **33**, 1834–1847 (2005).

[23] Yamada, T. & Morishita, S. Accelerated off-target search algorithm for siRNA. *Bioinformatics* **21**, 1316–1324 (2005).

[24] Zhao, W. & Lane, T. siRNA off-target search: A hybrid q-gram based filtering approach. In *Proceedings of the 5th International Workshop on Bioinformatics*, BIOKDD '05, 54–60 (ACM, New York, NY, USA, 2005).

[25] Alkan, F. *et al.* RIsearch2: suffix array-based large-scale prediction of RNA-RNA interactions and siRNA off-targets. *Nucleic Acids Research* **45**, e60 (2017).

[26] Doench, J. G. & Sharp, P. A. Specificity of microRNA target selection in translational repression. *Genes & Development* **18**, 504–511 (2004).

[27] Andoni, A. & Indyk, P. Near-optimal hashing algorithms for approximate nearest neighbor in high dimensions. *Proceedings of the Symposium on Foundations of Computer Science* (2006).

[28] Břinda, K., Sykulski, M. & Kucherov, G. Spaced seeds improve k-mer-based metagenomic classification. *Bioinformatics* **31**, 3584–3592 (2015).

[29] Gootenberg, J. S. *et al.* Nucleic acid detection with CRISPR-Cas13a/C2c2. *Science* **356**, 438–442 (2017).

[30] Chen, J. S. *et al.* CRISPR-Cas12a target binding unleashes indiscriminate single-stranded DNase activity. *Science* **360**, 436–439 (2018).

[31] Daher, R. K., Stewart, G., Boissinot, M. & Bergeron, M. G. Recombinase polymerase amplification for diagnostic applications. *Clinical Chemistry* **62**, 947–958 (2016).

[32] Federhen, S. The NCBI taxonomy database. *Nucleic Acids Research* **40**, D136–43 (2012).

[33] Bao, Y. *et al.* The influenza virus resource at the National Center for Biotechnology Information. *Journal of Virology* **82**, 596–601 (2008).

[34] Ondov, B. D. *et al.* Mash: fast genome and metagenome distance estimation using MinHash. *Genome Biology* **17**, 132 (2016).

[35] Katoh, K. & Standley, D. M. MAFFT multiple sequence alignment software version 7: improvements in performance and usability. *Molecular Biology and Evolution* **30**, 772–780 (2013).

[36] Yang, Z. *Computational Molecular Evolution* (Oxford University Press, Oxford; New York, 2006).

[37] Brister, J. R., Ako-Adjei, D., Bao, Y. & Blinkova, O. NCBI viral genomes resource. *Nucleic Acids Research* **43**, D571–7 (2015).
