# Supplementary material for: Designing sensitive viral diagnostics with machine learning
Source: Nat Biotechnol. Author manuscript; Available in PMC 2022 Jul 21. (PMC9287178; doi:10.1038/s41587-022-01213-5)
Supplement: Reporting Summary [file NIHMS1820058-supplement-Reporting_Summary.pdf]

nature research

Corresponding author(s): Hayden C. Metsky

Last updated by author(s): December 22, 2021

# Reporting Summary

Nature Research wishes to improve the reproducibility of the work that we publish. This form provides structure for consistency and transparency in reporting. For further information on Nature Research policies, see our Editorial Policies and the Editorial Policy Checklist.

## Statistics

For all statistical analyses, confirm that the following items are present in the figure legend, table legend, main text, or Methods section.

| n/a | Confirmed | |
|---|---|---|
| ☐ | ☒ | The exact sample size (*n*) for each experimental group/condition, given as a discrete number and unit of measurement |
| ☐ | ☒ | A statement on whether measurements were taken from distinct samples or whether the same sample was measured repeatedly |
| ☐ | ☒ | The statistical test(s) used AND whether they are one- or two-sided<br>*Only common tests should be described solely by name; describe more complex techniques in the Methods section.* |
| ☒ | ☐ | A description of all covariates tested |
| ☒ | ☐ | A description of any assumptions or corrections, such as tests of normality and adjustment for multiple comparisons |
| ☐ | ☒ | A full description of the statistical parameters including central tendency (e.g. means) or other basic estimates (e.g. regression coefficient) AND variation (e.g. standard deviation) or associated estimates of uncertainty (e.g. confidence intervals) |
| ☐ | ☒ | For null hypothesis testing, the test statistic (e.g. *F*, *t*, *r*) with confidence intervals, effect sizes, degrees of freedom and *P* value noted<br>*Give P values as exact values whenever suitable.* |
| ☒ | ☐ | For Bayesian analysis, information on the choice of priors and Markov chain Monte Carlo settings |
| ☒ | ☐ | For hierarchical and complex designs, identification of the appropriate level for tests and full reporting of outcomes |
| ☐ | ☒ | Estimates of effect sizes (e.g. Cohen's *d*, Pearson's *r*), indicating how they were calculated |

*Our web collection on statistics for biologists contains articles on many of the points above.*

## Software and code

Policy information about availability of computer code

| | |
|---|---|
| Data collection | Viral genomes used as input for designs were downloaded using ADAPT v1.0.0. |
| Data analysis | Analyses were performed using custom code available on GitHub at:<br>* Predictive modeling: https://github.com/broadinstitute/adapt-seq-design<br>* Designs across the vertebrate-infecting viral species: https://github.com/broadinstitute/adapt-designs-continuous<br>* Other analyses: https://github.com/broadinstitute/adapt-analysis<br>Models were trained and evaluated using TensorFlow 2.1.0 and scikit-learn 0.22. |

For manuscripts utilizing custom algorithms or software that are central to the research but not yet described in published literature, software must be made available to editors and reviewers. We strongly encourage code deposition in a community repository (e.g. GitHub). See the Nature Research guidelines for submitting code & software for further information.

## Data

Policy information about availability of data

All manuscripts must include a data availability statement. This statement should provide the following information, where applicable:

- Accession codes, unique identifiers, or web links for publicly available datasets
- A list of figures that have associated raw data
- A description of any restrictions on data availability

Data generated in this study is available on GitHub at:
* CRISPR-Cas13a library and activity dataset: https://github.com/broadinstitute/adapt-seq-design/tree/main/data
* Serialized trained models: https://github.com/broadinstitute/adapt-seq-design/tree/main/models/cas13
* Experimentally tested designs and their measured data: https://github.com/broadinstitute/adapt-designs/tree/main/experimentally-tested

# Field-specific reporting

Please select the one below that is the best fit for your research. If you are not sure, read the appropriate sections before making your selection.

☒ Life sciences ☐ Behavioural & social sciences ☐ Ecological, evolutionary & environmental sciences

For a reference copy of the document with all sections, see nature.com/documents/nr-reporting-summary-flat.pdf

# Life sciences study design

All studies must disclose on these points even when the disclosure is negative.

| | |
|---|---|
| Sample size | The CRISPR-Cas13a data was generated using 19,209 unique guide-target pairs. Sample size calculations were not performed and were chosen to be compatible with the CARMEN detection system, and sample sizes are sufficient because a learning curve (Supplementary Fig. 11) shows additional data points would not improve model performance. ADAPT's designs were evaluated using 88 guides and 290 synthetic targets (excluding controls); predetermining samples size is not applicable as no statistical tests were performed on this data. |
| Data exclusions | No data were excluded from the analyses of ADAPT's designs. Two experimental Cas13a guides were excluded from our model training data owing to low concentrations in their synthesis that yielded low activity, which we experimentally confirmed (exclusion criteria was not pre-established). |
| Replication | Measurements with CARMEN include about 10-20 replicate activity values per guide-target pair (Extended Data Fig. 2a). Replicates show concordance (Extended Data Fig. 2b,d). |
| Randomization | No randomization was performed because samples were not placed into groups. During model cross-validation, data points were not split randomly, but rather according to their position along the RNA target to ensure that validation folds contain sets of cognate guide-target pairs unrelated to data in the training folds. |
| Blinding | Investigators were not blinded to the contents of the samples containing synthetic targets representing known viruses. |

# Reporting for specific materials, systems and methods

We require information from authors about some types of materials, experimental systems and methods used in many studies. Here, indicate whether each material, system or method listed is relevant to your study. If you are not sure if a list item applies to your research, read the appropriate section before selecting a response.

## Materials & experimental systems

| n/a | Involved in the study |
|---|---|
| ☒ | ☐ Antibodies |
| ☒ | ☐ Eukaryotic cell lines |
| ☒ | ☐ Palaeontology and archaeology |
| ☒ | ☐ Animals and other organisms |
| ☒ | ☐ Human research participants |
| ☒ | ☐ Clinical data |
| ☒ | ☐ Dual use research of concern |

## Methods

| n/a | Involved in the study |
|---|---|
| ☒ | ☐ ChIP-seq |
| ☒ | ☐ Flow cytometry |
| ☒ | ☐ MRI-based neuroimaging |
